# Electrophilic oligodeoxynucleotide synthesis using dM-Dmoc for amino protection

**DOI:** 10.3762/bjoc.15.108

**Published:** 2019-05-20

**Authors:** Shahien Shahsavari, Dhananjani N A M Eriyagama, Bhaskar Halami, Vagarshak Begoyan, Marina Tanasova, Jinsen Chen, Shiyue Fang

**Affiliations:** 1 Department of Chemistry, Michigan Technological University, 1400 Townsend Drive, Houghton, Michigan 49931, USA

**Keywords:** Dmoc, electrophilic, oligonucleotides, protecting group, solid-phase synthesis

## Abstract

Solid-phase synthesis of electrophilic oligodeoxynucleotides (ODNs) was achieved using dimethyl-Dmoc (dM-Dmoc) as amino protecting group. Due to the high steric hindrance of the 2-(propan-2-ylidene)-1,3-dithiane side product from deprotection, the use of excess nucleophilic scavengers such as aniline to prevent Michael addition of the side product to the deprotected ODN during ODN cleavage and deprotection was no longer needed. The improved technology was demonstrated by the synthesis and characterization of five ODNs including three modified ones. The modified ODNs contained the electrophilic groups ethyl ester, α-chloroamide, and thioester. Using the technology, the sensitive groups can be installed at any location within the ODN sequences without using any sequence- or functionality-specific conditions and procedures.

## Introduction

After over 60 years of intensive research, the challenges for chemical oligodeoxynucleotide (ODN) synthesis have been considered largely overcome [1–4]. However, this is only true for unmodified ODNs at limited synthesis scales. For modified ODNs that contain sensitive functionalities including those that are unstable under acidic, basic and strongly nucleophilic conditions, many formidable challenges remain [2]. The reason is that during ODN synthesis using traditional technologies, the 5'-hydroxy group of nucleoside monomers is protected with the 4,4'-dimethoxytrityl (DMTr) group, which has to be removed with an acid in each synthetic cycle. The *exo*-amino groups of nucleosides dA, dC and dG are protected with acyl groups, the nascent ODN is anchored to a solid support via a base- or nucleophile-cleavable linker, and in the most widely used phosphoramidite technology the phosphate groups are protected with the 2-cyanoethyl group. These protecting groups and the linker have to be cleaved under strongly basic and nucleophilic conditions. As a result, many sensitive groups including acetal, hemiacetal, vinyl ethers, enol ethers, aldehydes, esters, activated esters, thioesters, aziridines, epoxides, alkyl halides, α-halocarbonyls, vinylpurines, methides and maleimides cannot or are difficult to be incorporated into ODNs, or cannot be installed at the desired locations in the ODNs. For example, to synthesize oligos that contain the epigenetically modified 5-formylcytosine, the aldehyde group had to be protected as a cyclic acetal instead of the more labile acyclic acetal [5–6]. The maleimide group was incorporated into ODNs as a Diels–Alder adduct with dimethylfuran. Besides the need of an additional step for deprotection, only examples of 5'-end modification was given probably due to the instability of the adduct under acidic conditions during ODN synthesis [7].

In recent years, applications of ODNs have extended to emerging areas such as nanotechnology [8–9], antisense drug development [10–12], DNA damage and repair [13–14], DNA methylation and demethylation [15–18], DNA–protein interactions [19–20], CRISPR genome editing [21–23], DNA data storage [24–25], synthetic biology [26], bioconjugation [27] and others [28–30]. These applications frequently require modified ODNs that contain a wide variety of functional groups including those that cannot survive known ODN synthesis, cleavage and deprotection conditions. To meet these demands, some work on developing new technologies suitable for the synthesis of sensitive ODNs has been carried out [28,31]. A common method is to use more labile acyl functions such as the phenoxyacetyl group for amino protection and as linker to enable deprotection and cleavage under milder basic conditions [32]. The palladium-labile allyl groups were also used for amino protection [33–34]. The *o*-nitrobenzyl function was used as linker to enable photo cleavage [34]. However, these methods are still not ideal. The phenoxyacetyl group and linker still need nucleophilic cleavage. Palladium is expensive and difficult to remove from ODN. Photoirradiation can damage ODNs. The (*p*-nitrophenyl)ethyl (Npe) and (*p*-nitrophenyl)ethyloxycarbonyl (Npeoc) groups were also explored for sensitive ODN synthesis under non-nucleophilic conditions [35–38]. The requirement of the strong base DBU in aprotic solvents over long hours in the presence of a nucleophilic scavenger for their cleavage could limit their application. In addition, in some cases the sequences synthesized by the method were short and the yields of the ODNs were low [35–38]. In the literature, there are also examples using post-synthesis modifications to introduce sensitive groups to ODNs [12]. However, these methods are case-specific, and their procedures are usually complicated. The ODN synthesis method without nucleobase protection could be considered for the incorporation of sensitive functionalities into ODNs [39]. However, a linker that can be cleaved under mild conditions is suitable for the purpose has not been identified. More seriously, high selectivity of *O*-phosphitylation over *N*-phosphitylation, which is crucial for practical applications especially for the synthesis of ODNs approaching 20-mer or longer, may not be easy to achieve.

**Scheme 1:**
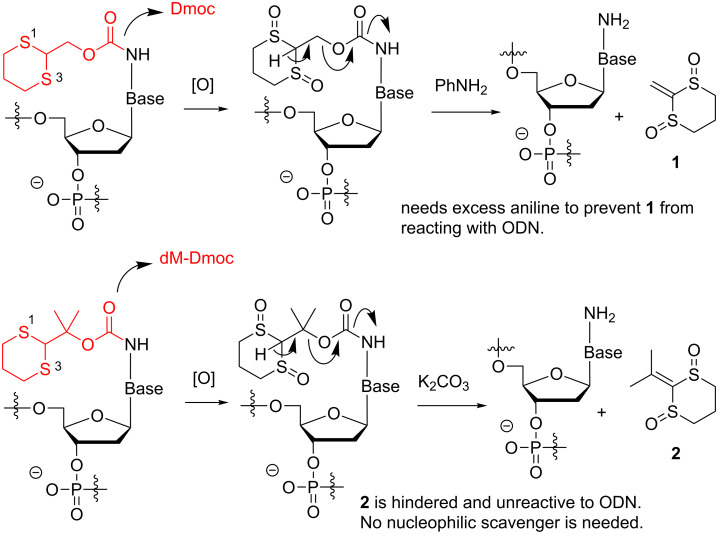
Comparison of Dmoc and dM-Dmoc as nucleobase protecting groups for ODN synthesis.

To develop a universal technology for the synthesis of ODNs that contain a wide variety of sensitive functionalities, we recently reported the use of 1,3-dithian-2-ylmethoxycabonyl (Dmoc) as protecting groups and linkers for ODN synthesis [40–41]. Due to the low acidity of H-2 (p*K*_a_ ≈31) in the Dmoc function, these groups and linkers were expected to be stable under ODN synthesis conditions. However, once the dithioketal in the group is oxidized, the acidity of H-2 (p*K*_a_ ≈12) is drastically increased [42–43]. Considering that the widely used Fmoc protecting group, of which the H-9 has a p*K*_a_ of ≈22 [42], can be readily removed with a weak base such as piperidine, we hypothesized that the oxidized Dmoc groups and linkers could be cleaved under weakly basic and non-nucleophilic conditions via β-elimination. Indeed, we found that the deprotection and cleavage could be achieved by oxidation with sodium periodate followed by treating with the mild base aniline at room temperature. Due to the mild deprotection and cleavage conditions, we concluded that the technology was suitable for the synthesis of sensitive ODNs that contain electrophilic groups. However, at the current state of art one drawback of the technology is that large excess aniline has to be used as a scavenger to prevent the deprotection side product **1** from reacting with the deprotected ODNs via Michael addition. Aniline is a weak nucleophile, but using large excess is not ideal for a technology aimed to be practically and universally useful. In this paper, we report the use of dimethyl-Dmoc (dM-Dmoc), which we previously studied for alkyl- and arylamine protections [44], in place of Dmoc for nucleobase protection for ODN synthesis (Scheme 1). Due to the steric hindrance of the side product **2** from deprotection, we found that a nucleophilic scavenger was no longer needed during deprotection, and the β-elimination step could be achieved using the non-nucleophilic weak base potassium carbonate.

## Results and Discussion

**Figure 1:**
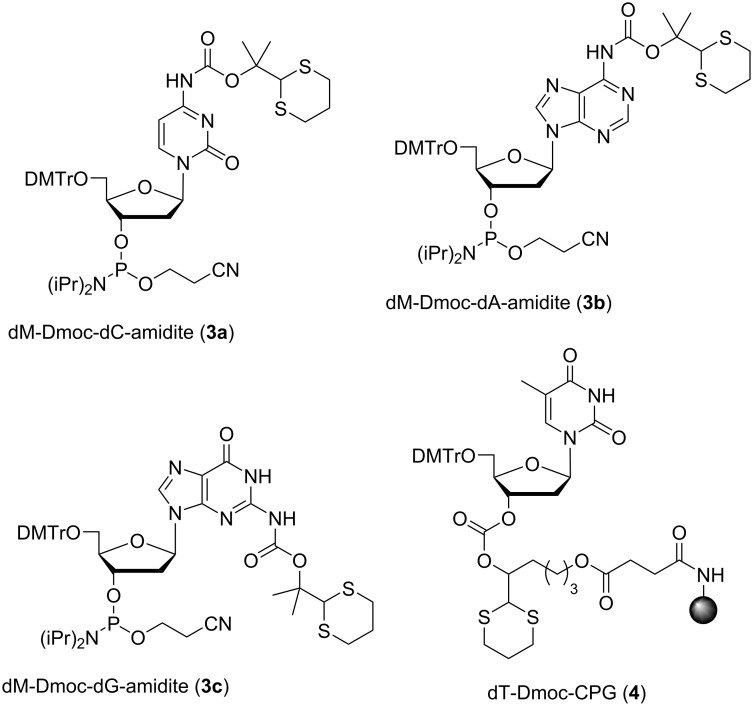
dM-Dmoc phosphoramidite monomers and CPG with Dmoc linker.

**Scheme 2:**
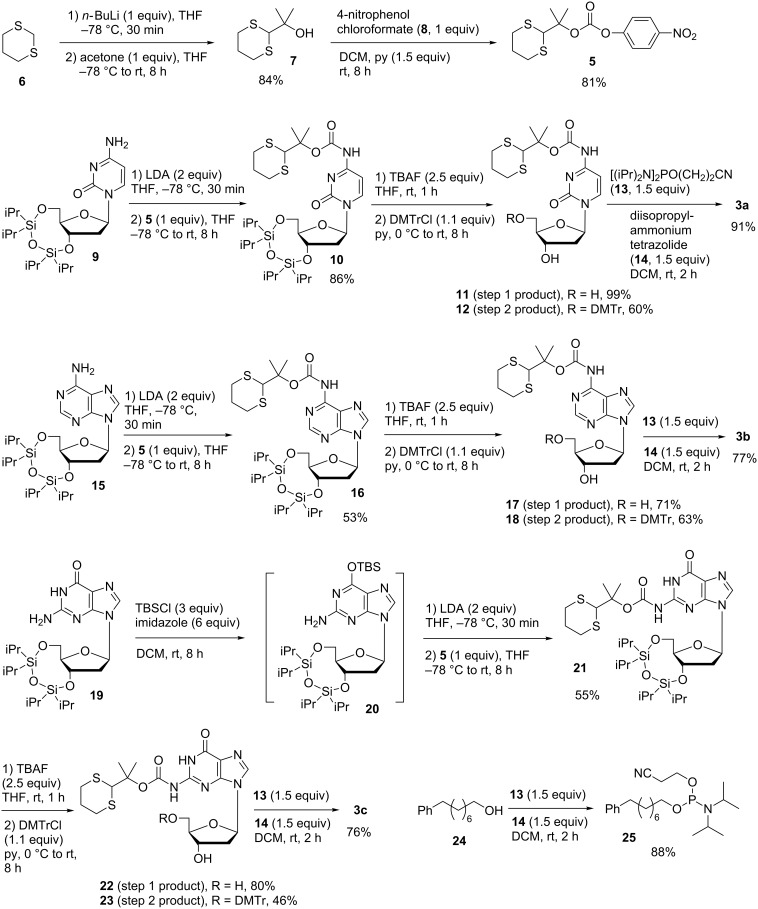
Synthesis of compound **5** [44], nucleoside phosphoramidite monomers **3a**–**c** and phosphoramidite capping agent **25**.

To develop the dM-Dmoc electrophilic ODN synthesis technology, the phosphoramidite monomers **3a**–**c** and the linker **4** (Figure 1) were needed. The construction of linker **4** was reported previously [40]. The synthesis of **3a**–**c** is shown in Scheme 2. The reagent **5** needed for protecting the *exo-*amino groups of nucleobases was prepared in two steps from 1,3-dithiane (**6**) according to a procedure we reported previously [44]. The dC phosphoramidite monomer **3a** was synthesized from compound **9** [45]. The formation of the hindered *O*-*tert*-alkyl *N*-arylcarbamate **10** was found highly challenging [44,46–47]. We tried many conditions and finally found that acceptable yields could be achieved under the highly reactive conditions involving two equivalents LDA and one equivalent **5**. The silyl protecting groups were then removed with TBAF giving compound **11** in 99% yield. Tritylation of **11** with DMTrCl gave **12**, which was phosphitylated with reagents **13** and **14** to give the target monomer **3a** (Scheme 2). The dA phosphoramidite monomer **3b** was synthesized similarly starting from **15** [48]. The amino group of **15** was carbamylated with **5** in the presence of two equivalents LDA to give **16**. The silyl groups were removed, and compound **17** was tritylated to give **18**, which was phosphitylated to give **3b**. The dG phosphoramidite monomer **3c** had to be synthesized using a slightly different procedure (Scheme 2). The amide function in the nucleobase in the silyl protected nucleoside **19** [45] was temporarily protected with TBSCl to give **20** [49]. This intermediate was not isolated and the *exo-*amino group was carbamylated directly with **5** in the presence of two equivalents LDA giving **21** in 55% yield. The silyl protecting groups were removed to give **22**, which was tritylated to give **23** and phosphitylated to give the target monomer **3c** (Scheme 2). As will be discussed later, we also needed the hydrophobic phosphoramidite **25** for developing the dM-Dmoc ODN synthesis technology. The compound was simply prepared from the commercially available **24** via phosphitylation using the reagents **13** and **14** (Scheme 2).

**Figure 2:**
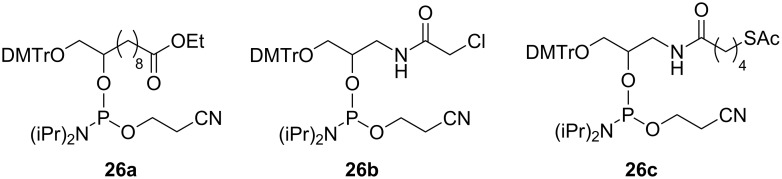
Structure of phosphoramidites containing electrophilic groups.

**Scheme 3:**
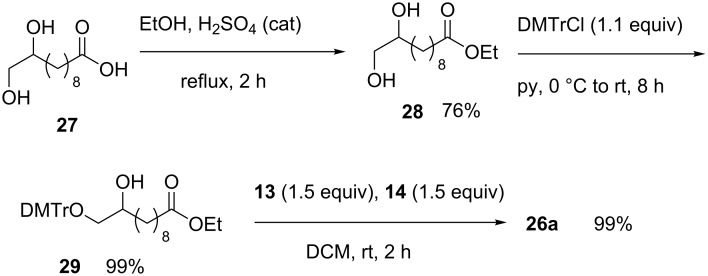
Synthesis of ester-containing phosphoramidite **26a**.

To demonstrate the capability of the dM-Dmoc ODN synthesis technology for incorporating electrophilic groups, we also needed phosphoramidite monomers **26a**–**c**, which contained the sensitive functionalities ester, α-chloroacetamide and thioester, respectively (Figure 2). The synthesis of **26b**,**c** has been reported [40]. Scheme 3 shows the synthesis of **26a**. The required 1,2-diol **28** was simply prepared from the commercially available **27** by esterification in ethanol. Cyclization or oligomerization of **27** was not an issue for the transformation. The primary alcohol of **28** was selectively tritylated with DMTrCl to give **29**, which was phosphitylated with **13** in the presence of **14** to give **26a**.

**Figure 3:**
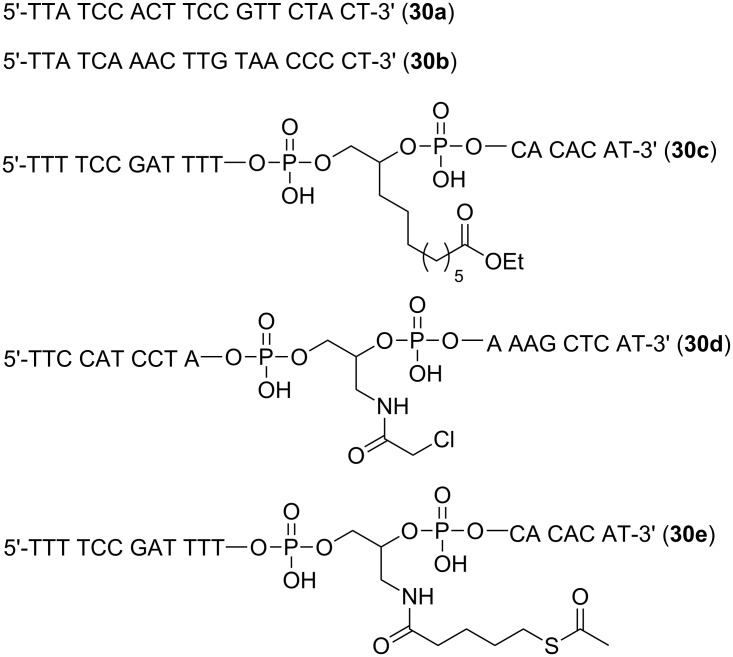
ODN sequences **30a–e**. Their 5'-tritylated versions are labeled as **30a-tr**, **30b-tr**, **30c-tr**, **30d-tr**, and **30e-tr**, respectively.

**Figure 4:**
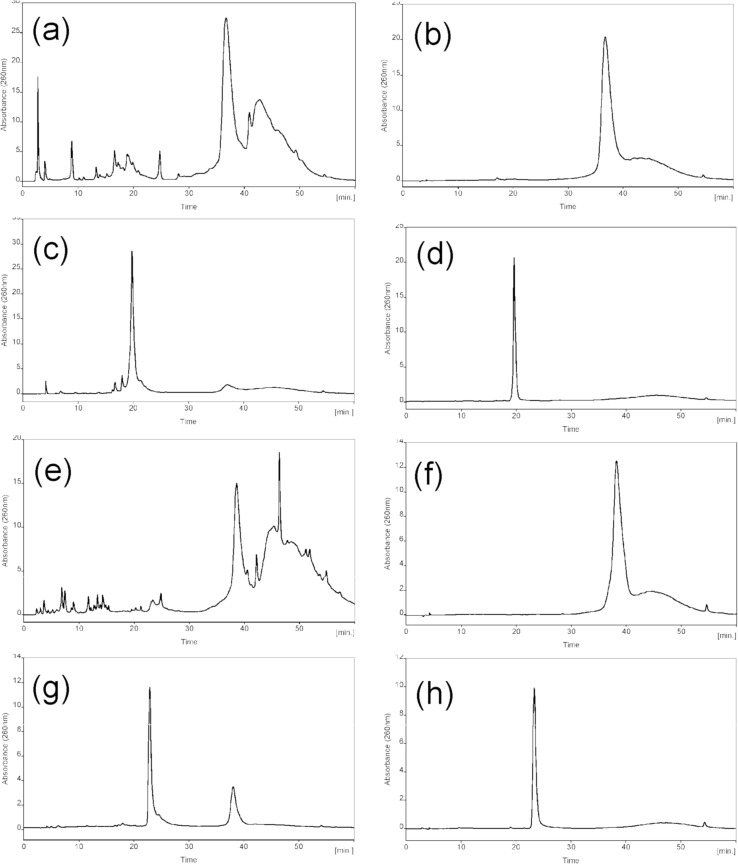
RP HPLC profiles of (a) crude **30a-tr**, (b) pure **30a-tr**, (c) crude **30a**, (d) pure **30a**, (e) crude **30c-tr**, (f) pure **30c-tr**, (g) crude **30c**, (h) pure **30c**. In profiles (a) and (e), the well-separated major peak before 40 minutes is the trityl-tagged full-length ODN. The peaks after 40 minutes are branched sequences with two or more trityl tags.

**Figure 5:**
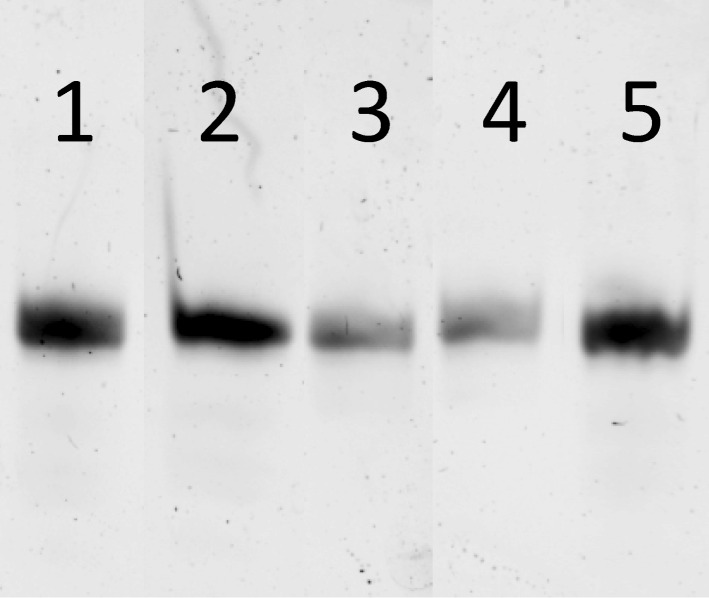
PAGE analyses of ODNs **30a**–**e**. Lanes 1–5 are ODNs **30a**–**e**, respectively.

**Figure 6:**
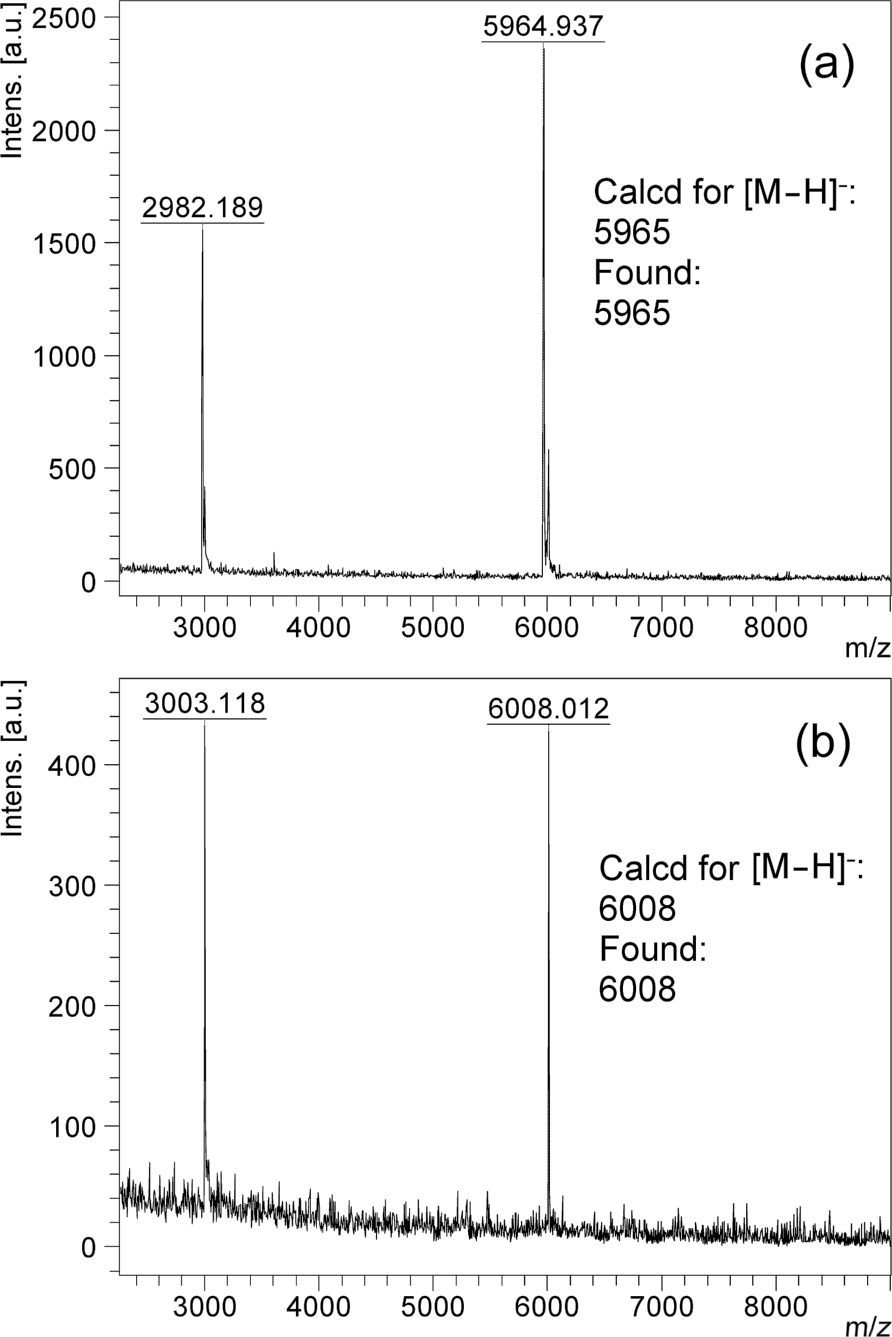
MALDI–TOF MS of (a) ODN **30a** and (b) **30c**.

**Scheme 4:**
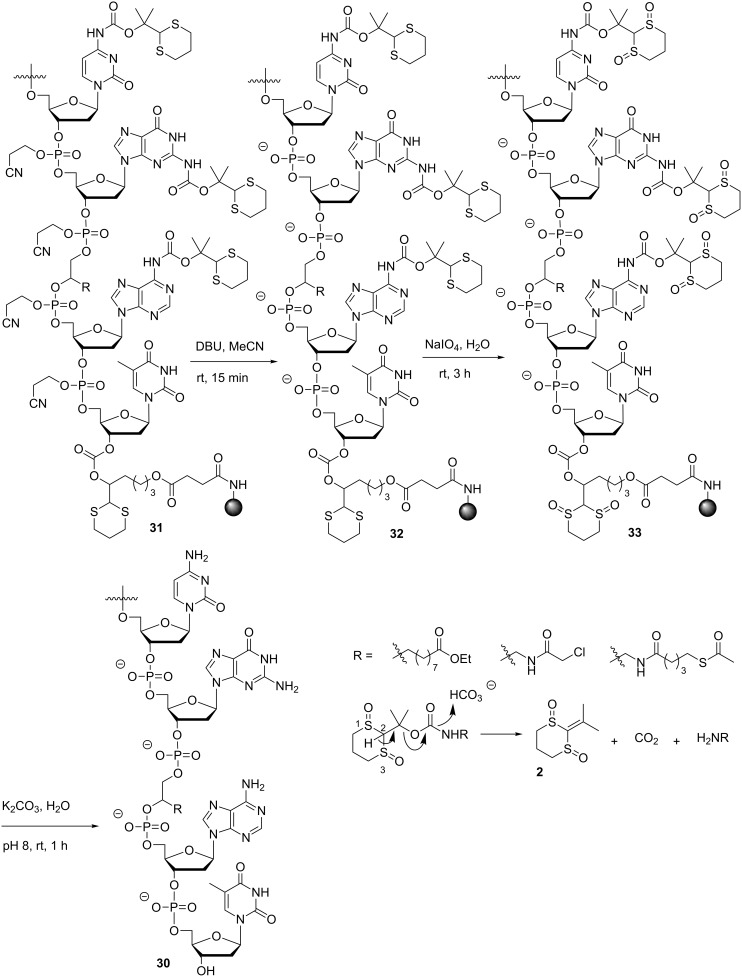
ODN deprotection and cleavage under non-nucleophilic conditions.

With the required phosphoramidite monomers **3a**–**c** and linker **4** in hand, we decided to identify suitable conditions for ODN synthesis, deprotection and cleavage under non-nucleophilic conditions by synthesizing the unmodified ODNs **30a**,**b** (Figure 3). The syntheses were conducted at a scale of 0.52 µmol on a MerMade 6 DNA/RNA synthesizer. The dT-Dmoc-CPG (**4**) was used as the solid support. Detritylation was carried out under standard conditions suggested by the synthesizer manufacturer for 1 µmol synthesis. The 0.1 M acetonitrile solutions of phosphoramidite monomers **3a**–**c** and the commercially available 5'-DMTr-β-cyanoethyl-dT phosphoramidite were used for incorporating dA, dC, dG and dT nucleotides, respectively. The coupling conditions were standard except that in some cases, coupling was increased from two to three times. Capping failure sequences was achieved using the phosphoramidite **25** with 5-(ethylthio)-1*H*-tetrazole as activator instead of the typically used acetic anhydride. Oxidation was performed under standard conditions. The last nucleotide at the 5'-end of ODN was incorporated with a 5'-trityl nucleoside phosphoramidite instead of a 5'-DMTr counterpart. At the end of the synthesis, the 5'-trityl group was not removed. More details about the synthesis are given in the Experimental section. For cleavage and deprotection under non-nucleophilic conditions, the ODN on CPG, which should appear as **31** (Scheme 4) with a 5'-trityl tag, was treated with a DBU solution in acetonitrile at room temperature briefly. This removed the β-cyanoethyl phosphate protecting groups to give **32**. HPLC analysis of the DBU solution did not found any ODN that was cleaved prematurely – an observation consistent with the slow rate of cleavage of succinyl-anchored ODNs from solid support under similar conditions [50]. The dithioketal groups in the dM-Dmoc and Dmoc functions of **32** were then oxidized with a solution of sodium periodate at room temperature to give **33**. The 5'-trityl tag survived the conditions. It should be pointed out that some sulfoxides might be further oxidized to sulfones, which should not affect the overall results of the deprotection and cleavage procedure. Removal of the oxidized dM-Dmoc protection groups and cleavage of the oxidized Dmoc linker were achieved with a solution of the weak non-nucleophilic base potassium carbonate at pH 8 at room temperature giving the fully deprotected 5'-trityl-tagged ODN **30** (Scheme 4). Purification of the ODN **30a** was achieved in two steps – trityl-on RP HPLC followed by trityl-off RP HPLC. For trityl-on HPLC (profile a, Figure 4), the desired full-length 5'-trityl-tagged ODN appeared at 36–39 minutes, and was well separated from other peaks. This peak was collected, and analyzed with RP HPLC (profile b). The purified 5'-trityl-tagged ODN was detritylated with 80% acetic acid. Even though it was reported that removal of trityl groups from a primary alcohol required two days at room temperature with 80% acetic acid [51], we found that our detritylation could reach completion or in some cases close to completion in three hours. After the acid was evaporated, the de-tritylated ODN was purified again with RP HPLC (profile c). The major peak at around 20 minutes was collected, the ODN from which showed a single sharp peak when analyzed with RP HPLC (profile d). The purified de-tritylated ODN was further analyzed with polyacrylamide gel electrophoresis (PAGE), a single band was observed (Lane 1, Figure 5). The HPLC purified ODN was also analyzed with MALDI–TOF MS, molecular mass corresponding to correct ODN structure was found (Figure 6). The unmodified ODN **30b** were synthesized, purified and analyzed under the same conditions. Its HPLC profiles and MS are given in Supporting Information File 1, and PAGE image is in Figure 5. All the analytical data indicate that the ODNs were pure and had correct identity.

In the RP HPLC profiles of the crude 5'-trityl-tagged ODNs such as that for **30a-tr** (profile a, Figure 4), besides failure sequences at around 20 minutes, there were multiple peaks after 40 minutes. We believe that those peaks were from branched ODNs generated from the premature deprotection of dM-Dmoc groups during ODN synthesis. The dM-Dmoc protections, which contained a tertiary butyl carbamate moiety, were not completely stable under the acidic conditions needed for de-tritylation in each synthetic cycle. Once the protection was lost, in the coupling step, incoming phosphoramidites would react with the free amino groups, and branched ODNs would be produced. Fortunately, these branched ODNs had two or more 5'-trityl groups, and therefore were significantly more hydrophobic than the desired ODN. During RP HPLC, they were eluted significantly later than the desired ODN and could be easily removed. We believe that the branching problem was not caused by premature oxidation of the dM-Dmoc groups by iodine in the oxidation step in ODN synthesis because the problem did not exist when Dmoc was used for ODN synthesis [41]. In addition, we also subjected 1,3-dithiane to the iodine oxidation conditions for over 24 hours, no oxidation could be detected.

Before using **25** for capping and a trityl group for 5'-tagging in ODN synthesis using **3a**–**c** and **4**, we tried to synthesize ODNs under standard conditions using acetic anhydride for capping and without tagging the 5'-end of ODNs. RP HPLC analyses showed that the peaks of the desired ODNs and branched sequences were very close and in some cases even overlapped, which made HPLC purification of the products difficult. A typical RP HPLC profile of ODNs synthesized in that manner is given in Supporting Information File 1. We therefore tried to keep the 5'-DMTr group at the end of solid phase synthesis to assist HPLC purification hypothesizing that the desired ODN with one DMTr group would be easy to be separated from any branched sequences that had two or more DMTr groups. This was indeed the case. A RP HPLC profile is given in Supporting Information File 1. However, the sodium periodate oxidation conditions used for ODN cleavage and deprotection were slightly acidic, and in most cases, we were not able to keep the DMTr groups. This problem made the method unreliable. We also tried to tag the ODN with the hydrophobic *tert*-butyldiphenylsilyl (TBDPS) group. In RP HPLC profiles, the desired TBDPS-tagged full-length sequence was also separated very well from the branched sequences (Supporting Information File 1). However, at this time we could not identify mild conditions to remove the tag after purification of the ODN. These experiments directed us to the use of the trityl tag to assist ODN purification as described above. The reason for us to use **25** instead of acetic anhydride for capping was based on two considerations. One was that if a branched sequence failed to react at one or more sites during coupling, capping with a hydrophobic agent would still make the branched sequence more hydrophobic than the desired full-length sequence. Another consideration was that with acetic anhydride for capping, chances existed for replacing the dM-Dmoc groups with acetyl group during capping due to the presence of acids such as pyridinium acetate and large excess of acetic anhydride. Once the capping exchange occurred, the ODN molecule with an acetyl group would not be useful because the acetyl group would not be deprotectable under the mild deprotection conditions. Using **25** for capping, such capping exchange would not occur.

After identifying suitable conditions for the synthesis of unmodified ODNs under non-nucleophilic conditions using the dM-Dmoc technology, studying the feasibility of the technology for the synthesis of modified ODNs containing ester, α-chloroacetamide and thioester groups was pursued. These groups are sensitive to nucleophiles and cannot survive the commonly used concentrated ammonium hydroxide deprotection and cleavage conditions. We have demonstrated that the so called UltraMild deprotection and cleavage conditions involving potassium carbonate in anhydrous methanol are incompatible with α-chloroacetamide and thioester [40]. These findings are easily understandable because the species responsible for the cleavage and deprotection under UltraMild conditions is potassium methoxide, which is a strong nucleophile. The ODNs **30c**–**e** were chosen as the targets for the current study. The synthesis, deprotection and cleavage conditions were the same as those for the unmodified ODNs. The electrophilic groups were introduced with **26a**–**c**, respectively. In all cases, we placed the groups in the middle of the sequences, which is significantly more challenging than placing them at the 5'-end. The fully deprotected crude ODNs **30c**–**e** were purified and analyzed as described for **30a**. The HPLC profiles of crude and pure **30c** are given in Figure 4. Its PAGE and MALDI–TOF MS images are in Figure 5 and Figure 6, respectively. All analytical data for **30d**,**e** are given in Supporting Information File 1. It is noted that aminolysis and hydrolysis of the sensitive groups in the ODNs, which were found to be a problem previously [41], were successfully avoided by using the dM-Dmoc protection strategy. For all the five ODNs (**30a**–**e**), their OD_260_ after HPLC purification were determined (Supporting Information File 1). They ranged from 2.32 to 6.68 for the 0.52 µmol syntheses. To have a direct comparison with standard ODN synthesis technology, we also synthesized **30a** using commercial phosphoramidites and 0.52 µmol **4** (Supporting Information File 1). After purification with RP HPLC, the OD_260_ of **30a** was determined to be 8.30. With these data, we were able to conclude that the dM-Dmoc phosphoramidites had similar coupling efficiency as commercial phosphoramidites and the overall yields of ODNs from the dM-Dmoc technology were at the same level of those from standard technologies.

The successful synthesis and HPLC purification of the above five ODNs demonstrated that dM-Dmoc is a viable choice for amino protection for electrophilic ODN synthesis. Compared with using Dmoc for ODN synthesis, the major advantage of using dM-Dmoc is that deprotection can be achieved without using any nucleophilic scavengers. Using Dmoc, during deprotection after sodium periodate oxidation, a large excess aniline is needed to induce the β-elimination (see Scheme 1) and to prevent the side product **1** from reacting with the deprotected ODN via Michael addition [40]. Aniline is a weak base and only mildly nucleophilic. Electrophiles that are compatible with ODNs but reactive toward it are rare. However, using a large excess of aniline could be a significant drawback. For example, many electrophiles could be considered unreactive to it, but in the presence of a large excess of it, problems might arise. In addition, its boiling point is high, alternative techniques other than simple evaporation has to be used for its removal. In order to accomplish our goal of developing a universally useful technology for electrophilic ODN synthesis, the dM-Dmoc technology is a logical extension of our previous effort [40]. Using dM-Dmoc, the side product of deprotection is **2**. We believe that **2** could not react with the nucleophiles on ODNs including hydroxy and amino groups. Even the reaction took place, a hindered four-substituted carbon center would be formed. Because the Michael addition reaction is reversible, the adducts would easily fall apart to give back un-modified ODNs. Indeed, due to the use of dM-Dmoc, we were able to induce β-elimination with potassium carbonate in the absence of any scavenger under mild conditions.

Besides the advantage of avoiding the use of excess aniline as a scavenger, in the new studies, we also found that the acetic acid used in our previous studies for sodium periodate oxidation could be omitted. In that report [40], for oxidation of the dithioketals in Dmoc, an acidic solution of sodium periodate adjusted to pH 2 with acetic acid was used. Under those conditions, β-elimination did not occur and the ODNs remained on the solid support during oxidation. This greatly facilitated the removal of excess sodium periodate and its reduced salts because they could be easily washed away with water. Otherwise, more expensive means such as size exclusion chromatography had to be used. In our new studies, we tested to perform the oxidation in the absence of acetic acid. We found that the pH of 0.4 M sodium periodate solution was around 4, and this solution did not cause premature β-elimination during oxidation. Therefore, the ODNs remained on the solid support under this significantly less acidic conditions. Because ODNs are inherently unstable under acetic conditions, avoiding the use of acetic acid and performing the cleavage and deprotection at nearly neutral pH could make the technology more useful. In addition, the scope of sensitive functionalities to be introduced to ODNs using the technology could be further extended. The finding of the stability of the Dmoc function in linker **4** after oxidation under nearly neutral conditions is also important for considering using the technology for oligoribonucleotides (ORNs) synthesis. One potential problem to use the technology for ORN synthesis is that during oxidation of the Dmoc and dM-Dmoc functions using sodium periodate, if the oxidized Dmoc in the linker were unstable, and the 2' and 3'-OH groups were exposed before sodium periodate were removed, the C–C bond between the 2' and 3' carbons could be cleaved. With the finding of the relatively high stability of the oxidized Dmoc function, we are more confident that the Dmoc associated technologies will be useful for ORN synthesis as well.

## Conclusion

In summary, we have extended the Dmoc-based electrophilic ODN synthesis technology to a new level, at which dM-Dmoc is used for protecting *exo*-amino groups of nucleobases. With this advancement, the previously used large excess aniline for scavenging the Michael acceptor side product during cleavage and deprotection was no longer needed. This makes the technology more convenient to use and could extend its scope on incorporating different sensitive functionalities into ODNs. In addition, we found that the sodium periodate oxidation step for cleavage and deprotection could be performed in the absence of acetic acid at nearly neutral conditions instead of previously used acidic conditions. Because ODNs and many functionalities are sensitive to acid, the significantly less acidic conditions will eliminate concerns of ODN damage and increase the scope of functionalities capable to be incorporated into ODNs. Using the technology, five ODNs including three modified ones containing the sensitive groups – ester, α-chloroamide and thioester – were successfully synthesized. We expect that the technology will become a useful tool for the synthesis of sensitive ODN analogs.

## Experimental

**ODN synthesis, deprotection, cleavage and characterization**: All ODNs were synthesized on dT-Dmoc-CPG [40] (**4**, 26 µmol/g loading, 20 mg, 0.52 µmol) using a MerMade 6 Synthesizer. dM-Dmoc phosphoramidites **3a**–**c** and the commercial 5'-DMTr-2-cyanoethyl-dT phosphoramidite were used as monomers. The conditions suggested by synthesizer manufacturer for 1 μmol synthesis were used except that coupling was optionally increased from 2 to 3 times and capping was achieved using **25** instead of acetic anhydride. Briefly, detritylation, DCA (3%, DCM), 90 s × 2; coupling, phosphoramidite (0.1 M, MeCN), 5-(ethylthio)-1*H*-tetrazole (0.25 M, MeCN), 60 s × 3 (or 2); capping, **25** (0.1 M, MeCN) and 5-(ethylthio)-1*H*-tetrazole (0.25 M, MeCN), 60 s × 3; oxidation, I_2_ (0.02 M, THF/pyridine/H_2_O, 70/20/10, v/v/v), 40 s. For incorporating the last nucleoside monomer, a 5'-trityl-2-cyanoethyldeoxynucleoside phosphoramidite instead of the 5'-DMTr counterpart (in the current cases, 2'-deoxy-5'-*O*-tritylthymidine-3'-*O*-*N*,*N*-diisopropylaminocyanoethylphosphoramidite [52] was used) was used. At the end of synthesis, the 5'-trityl group was kept. The CPG was divided into 10 equal portions. One portion was gently shaken in a solution of DBU/CH_3_CN (1:9, v/v, 1 mL) at rt for 15 min. The supernatant was removed with a pipette, and the CPG was washed with CH_3_CN (1 mL × 5). This removed the 2-cyanoethyl groups on the phosphate groups. To the CPG, aqueous NaIO_4_ (0.4 M, 1 mL) was added and the mixture was shaken at rt for 3 h. The supernatant was removed with a pipette, and the CPG was rinsed briefly with water (1 mL × 4). Alternatively, oxidation was achieved with 0.1 M NaIO_4_ (1 mL, rt, 1 h × 3). The CPG was then washed with H_2_O (1 mL × 4). This oxidized the dithioketals in the dM-Dmoc and Dmoc groups. HPLC analysis of the supernatant and washes indicated that the ODN was not cleaved from CPG at this time. To the CPG was added aqueous K_2_CO_3_ (0.05%, pH 8, 500 μL), and the mixture was shaken at rt for 30 min. The supernatant was transferred into a centrifugal tube. The process was repeated one time. The combined supernatant was concentrated to ≈100 μL and injected into RP HPLC to generate crude ODN trace [in some trials, before HPLC the combined supernatant (1 mL) was loaded on a polyacrylamide desalting column (10 mL) and eluted with H_2_O to remove the salts from the ODN]. Fractions of the major ODN peak at ≈39 min were collected, concentrated to ≈100 μL, and injected into HPLC to give the profile of purified trityl-tagged ODN. To the dried trityl-tagged ODN was added 1 mL of 80% AcOH, and the mixture was shaken gently at rt for 3 h. Volatiles were evaporated. The residue was dissolved in ≈100 μL water, and injected into RP HPLC. The major peak of de-tritylated ODN at ≈21 min was collected and concentrated to dryness. The residue was the pure de-tritylated ODN, which was dissolved in 100 μL water and injected into HPLC to generate the profile of pure de-tritylated ODN. The pure ODN was analyzed with PAGE and MALDI–TOF MS. Information about OD_260_ of the ODNs (**30a**–**e**) and a comparison of the synthesis yields of **30a** using the dM-Dmoc (OD_260_ of 0.52 µmol synthesis, 2.94) and standard (OD_260_ of 0.52 µmol synthesis, 8.30) ODN synthesis technologies are provided in the UV spectra section of the Supporting Information File 1.

## Supporting Information

File 1 Experimental details, compound characterization, and protocol for ODN cleavage and deprotection.

File 2 HPLC profiles, MALDI–TOF MS spectra, UV spectra, and OD_260_ values of ODNs, and NMR spectra of new compounds.

## References

[1] Ma S, Tang N, Tian J (2012). Curr Opin Chem Biol.

[2] Hogrefe R I, Midthune B, Lebedev A (2013). Isr J Chem.

[3] Kosuri S, Church G M (2014). Nat Methods.

[4] Abramova T (2013). Molecules.

[5] Schröder A S, Steinbacher J, Steigenberger B, Gnerlich F A, Schiesser S, Pfaffeneder T, Carell T (2014). Angew Chem, Int Ed.

[6] Schröder A S, Kotljarova O, Parsa E, Iwan K, Raddaoui N, Carell T (2016). Org Lett.

[7] Sánchez A, Pedroso E, Grandas A (2011). Org Lett.

[8] Yu H, Alexander D T L, Aschauer U, Häner R (2017). Angew Chem, Int Ed.

[9] Ohayon Y P, Sha R, Flint O, Liu W, Chakraborty B, Subramanian H K K, Zheng J, Chandrasekaran A R, Abdallah H O, Wang X (2015). ACS Nano.

[10] Carrette L L G, Gyssels E, Loncke J, Madder A (2014). Org Biomol Chem.

[11] Carrette L L G, Gyssels E, De Laet N, Madder A (2016). Chem Commun.

[12] Ali M M, Oishi M, Nagatsugi F, Mori K, Nagasaki Y, Kataoka K, Sasaki S (2006). Angew Chem, Int Ed.

[13] Cadet J, Davies K J A, Medeiros M H G, Di Mascio P, Wagner J R (2017). Free Radical Biol Med.

[14] Li H, Endutkin A V, Bergonzo C, Fu L, Grollman A, Zharkov D O, Simmerling C (2017). J Am Chem Soc.

[15] Li X, Yao B, Chen L, Kang Y, Li Y, Cheng Y, Li L, Lin L, Wang Z, Wang M (2017). Nat Commun.

[16] He Y-F, Li B-Z, Li Z, Liu P, Wang Y, Tang Q, Ding J, Jia Y, Chen Z, Li L (2011). Science.

[17] Kawasaki F, Murat P, Li Z, Santner T, Balasubramanian S (2017). Chem Commun.

[18] Carell T, Brandmayr C, Hienzsch A, Müller M, Pearson D, Reiter V, Thoma I, Thumbs P, Wagner M (2012). Angew Chem, Int Ed.

[19] Tinnefeld V, Sickmann A, Ahrends R (2014). Eur J Mass Spectrom.

[20] Schneeberger E-M, Breuker K (2017). Angew Chem, Int Ed.

[21] Klann T S, Black J B, Chellappan M, Safi A, Song L, Hilton I B, Crawford G E, Reddy T E, Gersbach C A (2017). Nat Biotechnol.

[22] Canver M C, Haeussler M, Bauer D E, Orkin S H, Sanjana N E, Shalem O, Yuan G-C, Zhang F, Concordet J-P, Pinello L (2018). Nat Protoc.

[23] Wang H-X, Li M, Lee C M, Chakraborty S, Kim H-W, Bao G, Leong K W (2017). Chem Rev.

[24] Organick L, Ang S D, Chen Y-J, Lopez R, Yekhanin S, Makarychev K, Racz M Z, Kamath G, Gopalan P, Nguyen B (2018). Nat Biotechnol.

[25] Scudellari M (2015). Proc Natl Acad Sci U S A.

[26] Ausländer S, Ausländer D, Fussenegger M (2017). Angew Chem, Int Ed.

[27] Olszewska A, Pohl R, Brázdová M, Fojta M, Hocek M (2016). Bioconjugate Chem.

[28] Meher G, Meher N K, Iyer R P (2017). Curr Protoc Nucleic Acid Chem.

[29] Zhang W, Tam C P, Wang J, Szostak J W (2016). ACS Cent Sci.

[30] Malvezzi S, Angelov T, Sturla S J (2017). Chem – Eur J.

[31] Virta P (2009). ARKIVOC.

[32] Johnsson R A, Bogojeski J J, Damha M J (2014). Bioorg Med Chem Lett.

[33] Hayakawa Y, Wakabayashi S, Kato H, Noyori R (1990). J Am Chem Soc.

[34] Matray T J, Greenberg M M (1994). J Am Chem Soc.

[35] García R G, Brank A S, Christman J K, Marquez V E, Eritja R (2001). Antisense Nucleic Acid Drug Dev.

[36] Avino A M, Eritja R (1994). Nucleosides Nucleotides.

[37] Aviñó A, Garcia R G, Marquez V E, Eritja R (1995). Bioorg Med Chem Lett.

[38] Eritja R, Robles J, Aviñó A, Alberico F, Pedroso E (1992). Tetrahedron.

[39] Ohkubo A, Ezawa Y, Seio K, Sekine M (2004). J Am Chem Soc.

[40] Lin X, Chen J, Shahsavari S, Green N, Goyal D, Fang S (2016). Org Lett.

[41] Halami B, Shahsavari S, Nelson Z, Prehoda L, Eriyagama D N A M, Fang S (2018). ChemistrySelect.

[42] Bordwell F G, Bares J E, Bartmess J E, Drucker G E, Gerhold J, McCollum G J, Van der Puy M, Vanier N R, Matthews W S (1977). J Org Chem.

[43] Bordwell F G, Drucker G E, Andersen N H, Denniston A D (1986). J Am Chem Soc.

[44] Shahsavari S, McNamara C, Sylvester M, Bromley E, Joslin S, Lu B-Y, Fang S (2018). Beilstein J Org Chem.

[45] Rodríguez-Muñiz G M, Marin M L, Lhiaubet-Vallet V, Miranda M A (2012). Chem – Eur J.

[46] Shahsavari S, Wigstrom T, Gooding J, McNamara C, Fang S (2018). Tetrahedron Lett.

[47] Shahsavari S, Gooding J, Wigstrom T, Fang S (2017). ChemistrySelect.

[48] Kim E-K, Switzer C (2014). Org Lett.

[49] Grøtli M, Douglas M, Beijer B, Eritja R, Sproat B (1997). Bioorg Med Chem Lett.

[50] Palom Y, Grandas A, Pedroso E (1998). Nucleosides Nucleotides.

[51] Gilham P T, Khorana H G (1958). J Am Chem Soc.

[52] Hotoda H, Momota K, Furukawa H, Nakamura T, Kaneko M, Kimura S, Shimada K (1994). Nucleosides Nucleotides.

