# Supplementary material for: Electrophilic oligodeoxynucleotide synthesis using dM-Dmoc for amino protection
Source: Beilstein J Org Chem. 2019 May 20;15:1116–28. doi: 10.3762/bjoc.15.108 (PMC6541367; doi:10.3762/bjoc.15.108)
Supplement: File 1 — Experimental details, compound characterization, and protocol for ODN cleavage and deprotection. [file Beilstein_J_Org_Chem-15-1116-s001.pdf]

BEILSTEIN JOURNAL OF ORGANIC CHEMISTRY

Supporting Information

for

Electrophilic oligodeoxynucleotide synthesis using dM-Dmoc for amino protection

Shahien Shahsavari, Dhananjani N. A. M. Eriyagama, Bhaskar Halami, Vagarshak Begoyan, Marina Tanasova, Jinsen Chen and Shiyue Fang

Beilstein J. Org. Chem. **2019,** *15,* 1116–1128. doi:10.3762/bjoc.15.108

Experimental details, compound characterization, and protocol for ODN cleavage and deprotection

License and Terms: This is a supporting information file under the terms of the Creative Commons Attribution License (http://creativecommons.org/licenses/by/4.0). Please note that the reuse, redistribution and reproduction in particular requires that the authors and source are credited.
The license is subject to the *Beilstein Journal of Organic Chemistry* terms and conditions: (https://www.beilstein-journals.org/bjoc)

Experimental details and compound characterization

General information: All reactions were performed in oven-dried glassware under argon using standard Schlenk techniques. Reagents and solvents available from commercial sources were used as received unless otherwise noted. Lcaa-CPG (pore size 497 Å) was purchased from Prime Synthesis, Inc. Polyacrylamide desalting column (5K MWCO, 10 mL) was purchased from Thermo Scientific. THF and CH_2Cl_2 was dried using an Innovative Technology Pure-Solv™ system. Pyridine, diisopropylamine and acetone were distilled over CaH_2 under nitrogen. Thin layer chromatography (TLC) was performed using Sigma-Aldrich TLC plates, silica gel 60F-254 over glass support, 250 µm thickness. Flash column chromatography was performed using SiliCycle silica gel, particle size 40–63 µm. ^{1}H, ^{13}C and ^{31}P NMR spectra were measured on a Varian UNITY INOVA spectrometer at 400, 100 and 162 MHz, respectively; chemical shifts (δ) were reported in reference to solvent peaks (residue $CHCl_3$ at δ 7.24 ppm for ^{1}H and $CDCl_3$ at δ 77.00 ppm for ^{13}C) and to H_3PO_4 (δ 0.00 ppm for ^{31}P). HRMS was obtained on a Thermo HR-Orbitrap Elite Mass Spectrometer. LRMS was obtained on a Thermo Finnigan LCQ Advantage Ion Trap Mass Spectrometer. MALDI–TOF MS were obtained on Bruker's microflex™ LRF MALDI–TOF System. ODNs were synthesized on a MerMade 6 solid phase synthesizer. RP HPLC was performed on a JASCO LC-2000Plus System: pump, PU-2089Plus Quaternary Gradient; detector UV-2075Plus. A C-18 reversed phase analytical column (5 µm diameter, 100 Å, 250 × 3.20 mm) was used. Solvent A: 0.1 M triethylammonium acetate, 5% acetonitrile. Solvent B: 90% acetonitrile. All profiles were generated by detecting absorbance at 260 nm using the linear gradient solvent system: solvent B (0%-45%) in solvent A over 60 min followed by solvent B (45%–100%) in solvent A over 20 min at a flow rate of 1.0 mL/min. PAGE of ODNs was run in a gel slide casted with a stock solution prepared using the recipe – 62.5 mL 40% acrylamide and bis-acrylamide (19:1), 52.55 g urea, 6.25 mL 10 × TBE (tris/borate/EDTA) buffer, and suitable amount of DI water for a total 100 mL solution. The gel slide was casted with 7 mL of the stock solution, 70 µL 10% $(NH_4)_2S_2O_4$, and 7 µL TMEDA (tetramethylethylenediamine). Electrophoresis was run in 10 × TBE buffer at 200 V by pre-run (without sample) for 30 min followed by actual run (with sample) for 90 min. The gel was stained with SYBR® Gold, and images were obtained with a BioRad Gel Doc™ XR+ Gel Documentation System.

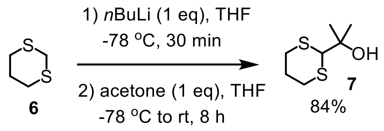

2-(1,3-Dithian-2-yl)propan-2-ol (7) [1]: To a solution of 1,3-dithiane (**6**, 5.0 g, 41.6 mmol) in dry THF (100 mL) was slowly added *n*-BuLi (2.5 M in pentane, 15.7 mL, 41.6 mmol) under argon at −78 °C. After stirring for 30 min, freshly distilled acetone (3.0 mL, 41.6 mmol) was added dropwise at −78 °C. The reaction was allowed to proceed for 8 h while warming to rt, and then quenched with saturated NH_4Cl (75 mL). The mixture was extracted with EtOAc (50 mL × 2). The extracts were combined and dried over anhydrous Na_2SO_4, filtered, and concentrated. The residue was purified with flash column chromatography (SiO_2, 4:1 hexanes/EtOAc) to afford **7** as a white solid (6.24 g, 84%) [1].

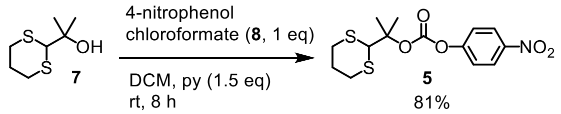

2-(1,3-Dithian-2-yl)propan-2-yl (4-nitrophenyl) carbonate (5) [1]: To a solution of **7** (6.4 g, 36 mmol) and pyridine (2.9 mL, 54 mmol) in DCM (100 mL) was added *p*-nitrophenyl chloroformate (**8**, 7.2 g, 36 mmol) at rt under argon. After stirring at rt for 8 h, the contents were poured into a separatory funnel and partitioned between EtOAc (40 mL) and H_2O (80 mL). The aqueous layer was extracted with DCM (50 mL × 2). The combined organic layer was dried over anhydrous Na_2SO_4, filtered and concentrated. Flash column chromatography (SiO_2, 9:1 hexanes/EtOAc) gave **5** as a white solid (10.0 g, 81%) [1].

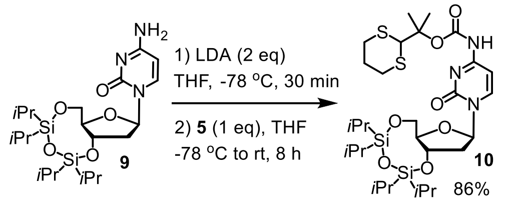

2'-Deoxy-*N*-(((2-(1,3-dithian-2-yl)propan-2-yl)oxy)carbonyl-3',5'-*O*-(1,1,3,3-tetrakis (1-methylethyl)-1,3-disiloxanediyl)cytidine (10): To a solution of diisopropylamine (1.2 mL, 8.5 mmol) in THF at −78 °C was added *n*-BuLi (2.5 M in pentane, 3.2 mL, 8.1 mmol) and stirred for 30 min. The freshly prepared LDA solution was added via a cannula to a solution of **9** (1.9 g, 4.05 mmol) in THF (50 mL) at −78 °C. After stirring for 30 min, compound **5** was added as a solid under positive nitrogen pressure at −78 °C. The mixture was stirred for 8 h while warming to rt. The contents were poured into a separatory funnel and partitioned between EtOAc (40 mL) and H_2O (40 mL). The aqueous layer was extracted with EtOAc (30 mL × 2). The combined organic layer was dried over anhydrous

Na_2SO_4, filtered and concentrated. Flash column chromatography (SiO_2, 1:1 hexanes/EtOAc) gave **10** as a white foam (2.33 g, 86%): R_f = 0.6 (1:2 hexanes/EtOAc); ^{1}H NMR (400 MHz, $CDCl_3$): δ 0.89-1.02 (m, 28H), 1.57 (s, 6H), 1.71-1.78 (m, 1H), 2.00-2.04 (m, 1H), 2.23-2.28 (m, 1H), 2.46-2.53 (m, 1H), 2.76-2.86 (m, 4H), 3.73 (d, J = 8.5 Hz, 1H), 3.93-3.97 (m, 1H), 4.09-4.12 (m, 1H), 4.27-4.33 (m, 1H), 4.92 (s, 1H), 5.98 (d, J = 6.5 Hz, 1H), 7.05 (d, J = 7.4 Hz, 1H), 8.10 (d, J = 7.4 Hz, 1H); ^{13}C NMR (100 MHz, $CDCl_3$): δ 12.5, 13.12, 13.16, 13.6, 16.9, 17.10, 17.12, 17.2, 17.5, 17.64, 17.67, 24.7, 25.9, 31.0, 39.9, 56.8, 60.0, 66.7, 85.2, 85.3, 85.7, 94.5, 143.9, 150.8, 154.9, 162.6; HRMS (ESI): *m/z* calcd for $C_{29}H_{52}N_3O_7S_2Si_2$ $[M + H]^+$ 674.2785, found 674.2783.

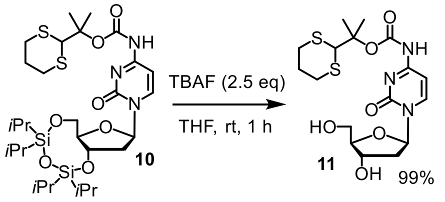

2'-Deoxy-*N*-(((2-(1,3-dithian-2-yl)propan-2-yl)oxy)carbonylcytidine (11): To the THF (10 mL) solution of **10** (800 mg, 1.19 mmol) at rt was added TBAF (1 M in THF, 3.0 mL, 3.0 mmol). The mixture was stirred for 1 h. THF was evaporated and the residue was loaded directly on a column. Flash column chromatography (SiO_2, 9.5:0.5 EtOAc/MeOH) gave **11** as a white foam (0.507 g, 99%): R_f = 0.3 (9.5:0.5 EtOAc/MeOH); ^{1}H NMR (400 MHz, CD_3OD): δ 1.60 (s, 6H), 1.70-1.77 (m, 1H), 2.04-2.10 (m, 1H), 2.12-2.18 (m, 1H), 2.43-2.49 (m, 1H), 2.82-2.94 (m, 4H), 3.71 (dd, J = 12.1, 3.8 Hz, 1H), 3.81 (dd, J = 12.1, 3.2 Hz, 1H), 3.96-3.99 (m, 1H), 4.33-4.37 (m, 1H), 4.81 (s, 2H), 5.07 (s, 1H), 6.19 (t, J = 6.2 Hz, 1H), 7.22 (d, J = 7.5 Hz, 1H), 8.40 (d, J = 7.5 Hz, 1H); ^{13}C NMR (100 MHz, CD_3OD): δ 24.0, 26.0, 30.7, 41.3, 56.8, 61.3, 70.4, 84.5, 87.3, 88.2, 95.5, 144.4, 151.6, 156.4, 163.5; HRMS (ESI): *m/z* calcd for $C_{17}H_{24}N_3O_6S_2$ $[M - H]^-$ 430.1107, found 430.1112.

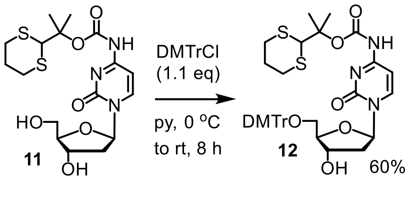

5'-*O*-[Bis(4-methoxyphenyl)phenylmethyl]-2'-deoxy-*N*-(((2-(1,3-dithian-2-yl)propan-2-yl)oxy)carbonylcytidine (12): To a solution of **11** (513 mg, 1.19 mmol) in pyridine (10 mL) at 0 °C was added DMTrCl (440 mg, 1.31 mmol) under positive nitrogen pressure. The mixture was stirred for 8 h while warming to rt. The volume of the mixture was reduced to about 2 mL under vacuum from an oil pump (small amount of pyridine was intentionally left to ensure basicity of the residue, which could help to avoid losing DMTr

from product). The residue was partitioned between 5% Na_2CO_3 (30 mL) and EtOAc (30 mL). The aqueous layer was extracted with EtOAc (15 mL × 2). The combined organic layer was dried over anhydrous Na_2SO_4, filtered and concentrated to dryness. Flash column chromatography (SiO_2, 9.5:0.5:0.5 EtOAc/MeOH/Et_3N) gave **12** as a white foam (523 mg, 60%): R_f = 0.5 (9.5:0.5:0.5 EtOAc/MeOH/Et_3N); ^1H NMR (400 MHz, $CDCl_3$): δ 1.57 (s, 6H), 1.68-1.79 (m, 1H), 1.99-2.03 (m, 1H), 2.14-2.21 (m, 1H), 2.67-2.73 (m, 1H), 2.80-2.83 (m, 4H), 3.31-3.34 (m, 1H), 3.41-3.44 (m, 1H), 3.73 (s, 6H), 4.15 (d, J = 3.3 Hz,1H), 4.49 (d, J = 4.3 Hz, 1H), 4.93 (s, 1H), 6.2 (t, J = 5.2 Hz, 1H), 6.79 (d, J = 8.3 Hz, 4H), 6.90 (d, J = 7.3 Hz, 1H), 7.16 (t, J = 7.0 Hz, 1H), 7.25 (d, J = 8.6 Hz, 4H), 7.36 (d, J = 7.6 Hz, 2H), 8.03 (bs, 1H), 8.23 (d, J = 7.4 Hz); ^{13}C NMR (100 MHz, $CDCl_3$): δ 24.8, 26.0, 31.1, 42.3, 55.4, 56.9, 62.9, 70.6, 85.1, 86.7, 86.9, 87.4, 95.2, 113.5, 127.2, 128.2, 128.4, 130.1, 130.2, 135.7, 135.9, 144.4, 150.9, 155.6, 158.7, 162.6; HRMS (ESI): *m/z* calcd for $C_{38}H_{44}N_3O_8S_2$ $[M + H]^+$ 734.2569, found 734.2565.

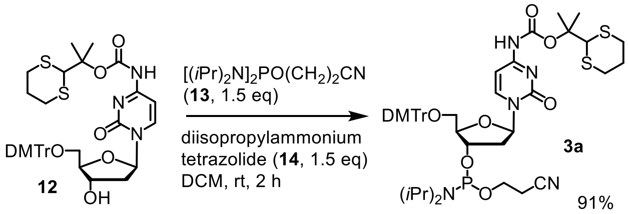

5'-*O*-(Bis(4-methoxyphenyl)phenylmethyl)-2'-deoxy-*N*-(((2-(1,3-dithian-2-yl)propan-2-yl)oxy)carbonylcytidine-3'-*O*-(*O*-(2-cyanoethyl)-*N*,*N*-diisopropylphosphoramidi-te) (3a): To a solution of **12** (500 mg, 0.682 mmol) and diisopropylammonium tetrazolide (**14**, 175 mg, 1.02 mmol) in DCM (10 mL) at rt was added 2-cyanoethyl *N*,*N*,*N'*,*N'*-tetraisopropylphosphorodiamidite (**13**, 325 µL, 1.02 mmol). After stirring at rt for 2 h, the reaction mixture was concentrated and loaded directly on a column. Flash column chromatography (SiO_2, 4:1:0.25 EtOAc/hexanes/Et_3N) gave **3a** as a white foam (580 mg, 91%): Mixture of two diastereoisomers; R_f = 0.3 and 0.4 (EtOAc); ^1H NMR (400 MHz, $CDCl_3$): δ 1.03 (d, J = 6.7 Hz, 2H), 1.11-1.20 (m, 12H), 1.23-1.30 (m, 3H), 1.61 (s, 6H), 2.02-2.08 (m, 1H), 1.73-1.83 (m, 1H), 2.02-2.08 (m, 1H), 2.18-2.29 (m, 2H), 2.40 (t, J = 6.4 Hz, 1H), 2.57 (t, J = 6.4 Hz, 1H), 2.65-2.75 (m, 2H), 2.81-2.89 (m, 4H), 3.29-3.36 (m, 1H), 3.45-3.60 (m, 5H), 3.78 (d, J = 3.5 Hz, 6H), 4.14-4.18 (m, 1H), 4.53-4.62 (m, 1H), 4.92 (s, 1H), 6.20-6.26 (m 1H), 6.82 (t, J = 7.9 Hz, 5H), 7.26 (t, J = 7.7 Hz, 6H), 7.37 (t, J = 7.2 Hz, 2H), 8.17 (d, J = 8.0 Hz, 1H), 8.26 (d, J = 7.7 Hz, 1H); ^{13}C NMR (100 MHz, $CDCl_3$): δ 20.3, 24.7, 26.0, 31.0, 41.0, 41.4, 43.4, 55.4, 56.8, 58.5, 62.1, 62.5, 85.2, 85.7, 87.0, 94.8, 113.4, 117.7, 128.1, 128.4, 128.5, 130.21, 130.26, 130.3, 135.6, 144.2, 150.8, 155.0, 158.8, 162.4; ^{31}P NMR (162 MHz, $CDCl_3$): δ 149.7, 150.4; HRMS (ESI): *m/z* calcd for $C_{47}H_{61}N_5O_9PS_2$ $[M + H]^+$ 934.3648, found 934.3652.

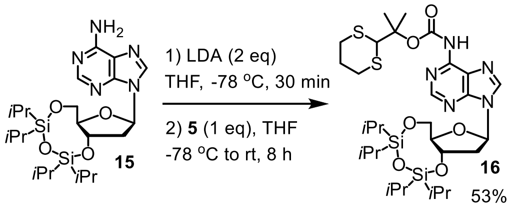

2'-Deoxy-*N*-(((2-(1,3-dithian-2-yl)propan-2-yl)oxy)carbonyl-3',5'-*O*-(1,1,3,3-tetrakis (1-methylethyl)-1,3-disiloxanediyl)adenosine (16): The procedure for synthesizing **10** was used. After flash column chromatography (SiO_2, 1:2 EtOAc/hexanes) **16** was afforded as a white foam in 53% yield: R_f = 0.5 (1:1 EtOAc/hexanes); ^{1}H NMR (400 MHz, $CDCl_3$): δ 0.97-1.05 (m, 28H), 1.61 (d, J = 7.5 Hz, 6H), 1.72-1.78 (m, 1H), 2.01-2.07 (m, 1H), 2.60-2.68 (m, 2H), 2.79-2.90 (m, 4H), 3.85-3.88 (m, 1H), 4.01 (t, J = 4.0 Hz, 2H), 4.91 (q, J = 7.6 Hz, 1H), 5.14 (s, 1H), 6.28-6.30 (m, 1H), 8.19 (s, 1H), 8.68 (s, 1H); ^{13}C NMR (100 MHz, $CDCl_3$): δ 12.7, 13.0, 13.2, 13.5, 17.0, 17.1, 17.2, 17.3, 17.5, 17.5, 17.6, 17.6, 40.2, 56.9, 61.8, 69.8, 83.6, 84.7, 85.4,122.5, 141.3, 149.4, 149.9, 150.2, 152.9; HRMS (ESI): *m/z* calcd for $C_{30}H_{52}N_5O_6S_2Si_2$ $[M + H]^+$ 698.2897, found 698.2896.

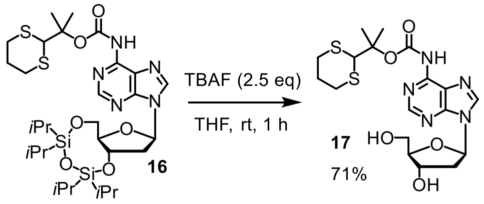

2'-Deoxy-*N*-(((2-(1,3-dithian-2-yl)propan-2-yl)oxy)carbonyladenosine (17): The procedure for synthesizing **11** was used. After flash column chromatography (SiO_2, 9:1 EtOAc/MeOH) **17** was afforded as a white foam in 71% yield: R_f = 0.3 (9:1 EtOAc/MeOH); ^{1}H NMR (400 MHz, $CDCl_3$): δ 1.67 (s, 6H), 1.69-1.83 (m, 1H), 2.03-2.09 (m, 1H), 2.34-2.37 (m, 2H), 2.84-2.90 (m, 4H), 2.93-3.08 (m, 1H), 3.81-3.97 (m, 2H), 4.24 (s, 1H), 4.82 (s, 1H), 5.17 (s, 1H), 5.84-5.87 (m, 1H), 6.36-6.40 (m, 1H), 8.02 (s, 1H), 8.42 (s, 1H), 8.73 (s, 1H); ^{13}C NMR (100 MHz, $CDCl_3$) δ 24.9, 26.1, 31.2, 41.1, 57.1, 63.5, 73.3, 85.0, 87.8, 89.7, 123.4, 142.4, 149.2, 150.1, 150.6, 152.5; HRMS (ESI): *m/z* calcd for $C_{18}H_{26}N_5O_5S_2$ $[M + H]^+$ 456.1375, found 456.1381.

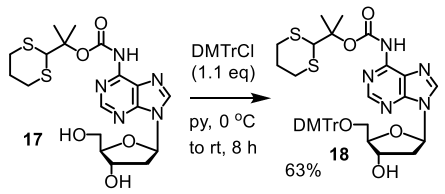

5'-*O*-[Bis(4-methoxyphenyl)phenylmethyl]-2'-deoxy-*N*-(((2-(1,3-dithian-2-yl)propan-2-yl)oxy)carbonyladenosine (18): The procedure for synthesizing **12** was used. After flash column chromatography (SiO_2, 9:0.5:0.5 EtOAc/MeOH/Et_3N) **18** was afforded as a white foam in 63% yield: R_f = 0.4 (9.5:0.5 EtOAc/MeOH); ^{1}H NMR (400 MHz, $CDCl_3$): δ 1.60 (s, 6H), 1.67-1.75 (m, 1H), 1.95-2.04 (m, 1H), 2.49-2.57 (m, 1H), 2.73-2.86 (m, 5H), 3.34 (d, J = 3.8 Hz, 2H), 3.67 (s, 6H), 4.16-4.21 (m, 1H), 4.66-4.70 (m, 1H), 5.10 (s, 1H), 5.21 (s, 1H), 6.44 (t, J = 6.3 Hz, 1H), 6.69 (d, J = 8.7 Hz, 4H), 7.07-7.21 (m, 7H), 7.30 (d, J = 8.0 Hz, 2H), 8.08 (s, 1H), 8.63 (s, 1H), 8.99 (bs, 1H); ^{13}C NMR (100 MHz, $CDCl_3$): δ 24.9, 26.0, 31.1, 40.6, 55.4, 57.0, 63.8, 72.3, 84.6, 84.9, 86.7, 113.3, 122.1, 127.1, 128.0, 128.2, 130.1, 141.3, 144.6, 149.5, 149.9, 150.7, 152.9, 158.6; HRMS (ESI): *m/z* calcd for $C_{39}H_{44}N_5O_7S_2$ [M + H]$^+$ 758.2682, found 758.2685.

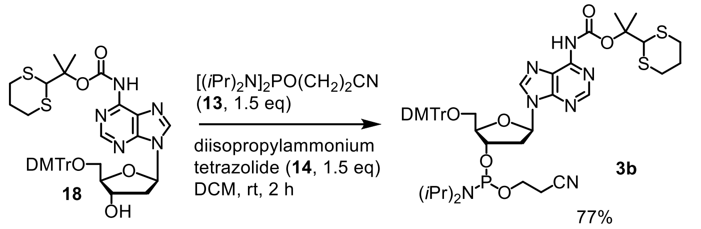

5'-*O*-(Bis(4-methoxyphenyl)phenylmethyl)-2'-deoxy-*N*-(((2-(1,3-dithian-2-yl)propan-2-yl)oxy)carbonyladenosine-3'-*O*-(*O*-(2-cyanoethyl)-*N*,*N*-diisopropylphosphor-amidite) (3b): The procedure for synthesizing **3a** was used. After flash column chromatography (SiO_2, 2:1:0.15 EtOAc/hexanes/Et_3N) **3b** was afforded as a white foam in 77% yield: Mixture of two diastereoisomers; R_f = 0.3 and 0.4 (2:1 EtOAc/hexanes); ^{1}H NMR (400 MHz, $CDCl_3$): δ 1.15-1.23 (m, 12H), 1.63 (s, 6H), 1.74-1.80 (m, 1H), 2.02-2.07 (m, 1H), 2.42 (t, J = 6.4 Hz, 1H), 2.57 (t, J = 6.4 Hz, 1H), 2.79-2.91 (m, 4H), 3.28-3.33 (m, 1H), 3.38-3.44 (m, 2H), 3.54-3.60 (m, 2H), 3.75 (s, 6H), 3.79-3.87 (m, 1H), 4.08-4.14 (m, 1H), 4.24-4.29 (m, 1H), 4.71-4.77 (m, 1H), 5.14 (s, 1H), 6.40-6.45 (m, 1H), 6.72-6.75 (m, 4H), 7.14-7.25 (m, 7H), 7.33-7.35 (m, 2H), 8.11 (d, J = 9.5 Hz, 1H), 8.38 (bs, 1H), 8.65 (s, 1H); ^{13}C NMR (100 MHz, $CDCl_3$): δ 20.1, 20.7, 22.5, 23.4, 24.8, 25.0, 26.2, 31.3, 39.7, 45.3, 45.5, 45.8, 55.4, 57.0, 58.5, 58.7, 63.6, 73.6, 74.4, 84.7, 84.7, 84.9, 86.0, 86.2, 86.7, 113.2, 117.6, 122.4, 127.0, 127.9, 128.2, 130.1, 135.7, 141.3, 144.5, 149.2, 149.7, 150.8, 152.8, 158.6; ^{31}P NMR (162 MHz, $CDCl_3$): δ 149.7, 149.9; HRMS (ESI)**:** *m/z* calcd for $C_{48}H_{60}N_7O_8PS_2H$ [M + H]$^+$ 958.3760, found 958.3769.

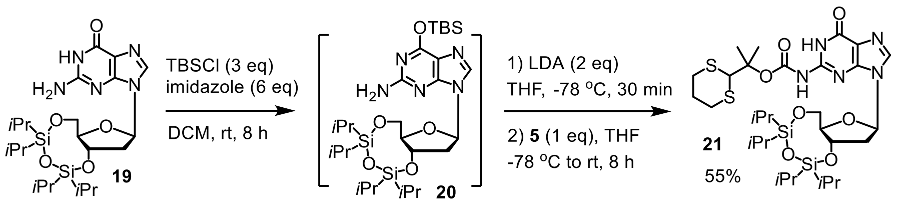

2'-Deoxy-*N*-(((2-(1,3-dithian-2-yl)propan-2-yl)oxy)carbonyl)-3',5'-*O*-(1,1,3,3-tetrakis (1-methylethyl)-1,3-disiloxanediyl)guanosine (21): The amide functionality in **19** was protected with a TBS group by reacting with TBSCl (3 equiv) in the presence of imidazole (6 equiv.) in DCM at rt for 8 h [2]. The crude intermediate **20** was partitioned between DCM and NaH_2PO_4/Na_2HPO_4 buffer (pH 7) and further washed with the buffer two times. The organic phase was dried over Na_2SO_4, filtered and concentrated to dryness. After the crude intermediate was dried under high vacuum over Drierite, **20** was converted to **21** following the procedure for synthesizing **10**. The TBS group probably fell off during partition between EtOAc and saturated NH_4Cl. After flash column chromatography (SiO_2, 1:1 EtOAc/hexanes) **21** was afforded as a brown foam in 55% yield: R_f = 0.3 (1:1 EtOAc/hexanes); ^1H NMR (400 MHz, $CDCl_3$): δ 0.96-1.08 (m, 28H), 1.63 (s, 6H), 1.77-1.85 (m, 1H), 2.08-2.15 (m, 1H), 2.52-2.55 (m, 2H), 2.85-2.92 (m, 4H), 3.81-3.85 (m, 1H), 3.94-4.04 (m, 2H), 4.71 (q, J = 7.4 Hz, 1H), 4.96 (s, 1H), 6.08 (t, J = 5.2 Hz, 1H), 7.82 (s, 1H); ^{13}C NMR (100 MHz, $CDCl_3$): δ 12.8, 13.3, 13.4, 13.7, 17.20, 17.28, 17.3, 17.45, 17.47, 17.5, 17.6, 17.7, 17.8, 24.9, 26.0, 31.4, 40.3, 57.0, 62.0, 70.2, 82.8, 85.4, 86.9, 121.5, 136.7, 146.7, 147.3, 151.8, 155.6; HRMS (ESI)**:** *m/z* calcd for $C_{30}H_{52}N_5O_7S_2Si_2$ [M + H]$^+$ 714.2847, found 714.2842.

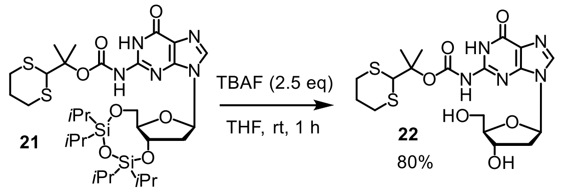

2'-Deoxy-*N*-(((2-(1,3-dithian-2-yl)propan-2-yl)oxy)carbonylguanosine (22): The procedure for synthesizing **11** was used. After flash column chromatography (SiO_2, 4:1 EtOAc/MeOH) **22** was afforded as a brown foam in 80% yield: R_f = 0.2 (9:1 EtOAc/MeOH); ^1H NMR (400 MHz, CD_3OD): δ 1.79 (s, 6H), 1.70-1.79 (m, 1H), 2.00-2.10 (m, 1H), 2.39-2.44 (m, 1H), 2.60-2.70 (m, 1H), 2.85-2.92 (m, 4H), 3.22 (s, 1H), 3.70-3.76 (m, 2H), 3.92-4.05 (m, 2H), 4.50-4.54 (m, 1H), 5.16 (s, 1H), 8.20 (s, 1H); ^{13}C NMR (100 MHz, CD_3OD): δ 23.6, 30.6, 40.4, 56.7, 61.8, 71.1, 84.3, 85.8, 88.0, 119.6, 138.2, 148.0, 153.7, 156.3; HRMS (ESI)**:** *m/z* calcd for $C_{18}H_{26}N_5O_6S_2$ [M + H]$^+$ 472.1324, found 472.1326.

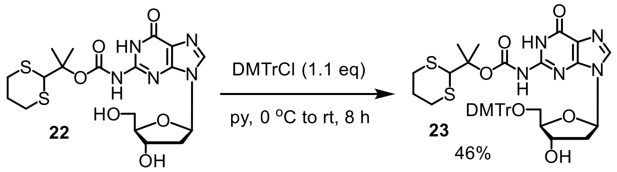

5'-*O*-[Bis(4-methoxyphenyl)phenylmethyl]-2'-deoxy-*N*-(((2-(1,3-dithian-2-yl)propan-2-yl)oxy)carbonylguanosine (23): The procedure for synthesizing **12** was used. After flash column chromatography (SiO_2, 9:0.5:0.5 EtOAc/MeOH/Et_3N) **23** was afforded as a brown foam in 46% yield: R_f = 0.4 (9.5:0.5 EtOAc/MeOH); ^{1}H NMR (400 MHz, $CDCl_3$): δ 1.58 (s, 6H), 1.68-1.77 (m, 1H), 1.97-2.03 (m, 1H), 2.50-2.60 (m, 2H), 2.77-2.85 (m, 4H), 3.25-3.29 (m, 2H), 3.67 (s, 6H), 4.14-4.22 (m, 1H), 4.69-4.76 (m, 1H), 4.93 (s, 1H), 6.23 (t, J = 6.2 Hz, 1H), 6.69 (d, J = 8.7 Hz, 4H), 7.07-7.13 (m, 2H), 7.21 (d, J = 8.6 Hz, 4H), 7.31 (d, J = 7.3 Hz, 2H), 7.75 (s, 1H); ^{13}C NMR (100 MHz, $CDCl_3$): δ 24.7, 25.9, 31.1, 40.6, 55.3, 60.6, 64.4, 72.0, 84.6, 86.5, 86.7, 113.2, 120.8, 127.9, 128.3, 130.1, 135.9, 137.7, 144.7, 147.1, 148.6, 152.7, 155.9, 158.6; HRMS (ESI)**:** *m/z* calcd for $C_{39}H_{44}N_5O_8S_2$ $[M + H]^+$ 774.2631, found 774.2629.

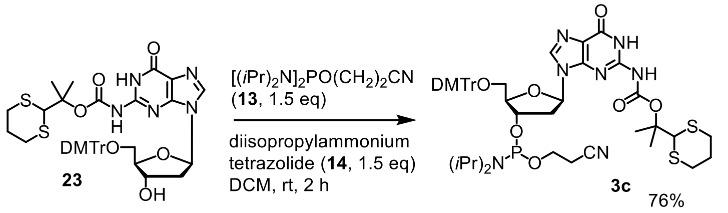

5'-*O*-(Bis(4-methoxyphenyl)phenylmethyl)-2'-deoxy-*N*-(((2-(1,3-dithian-2-yl)propan-2-yl)oxy)carbonylguanosine-3'-*O*-(*O*-(2-cyanoethyl)-*N*,*N*-diisopropylphosphor-amidite) (3c): The procedure for synthesizing **3a** was used. After flash column chromatography (SiO_2, 9.5:0.5 EtOAc/Et_3N) **3c** was afforded as a brown foam in 76% yield: Mixture of two diastereoisomers; R_f = 0.4 and 0.5 (EtOAc); ^{1}HNMR (400 MHz, $CDCl_3$): δ 1.08-1.25 (m, 12H), 1.59 (d, J = 7.9 Hz, 6H), 1.74-1.85 (m, 1H), 2.06-2.14 (m, 1H), 2.37-2.47 (m, 2H), 2.68-2.74 (m, 1H), 2.83-2.91 (m, 4H), 3.26-3.32 (m, 2H), 3.49-3.60 (m, 2H), 3.74 (s, 6H), 4.08-4.16 (m, 1H), 4.20-4.27 (s, 1H), 4.62-4.71 (m, 1H), 4.97 (s, 1H), 6.11-6.19 (m, 1H), 6.75 (d, J = 8.4 Hz, 4H), 7.15-7.28 (m, 7H), 7.36-7.38 (m, 2H), 7.72 (s, 1H); ^{13}C NMR (100 MHz, $CDCl_3$): δ 20.2, 20.6, 23.10, 23.18, 24.7, 24.8, 26.0, 31.2, 39.7, 43.3, 43.5, 45.5, 55.4, 56.8, 58.3, 63.9, 74.7, 84.9, 86.5, 86.6, 113.3, 117.6, 121.6, 127.1, 128.0, 130.1, 135.8, 137.2, 137.5, 144.6, 144.7, 148.3, 152.1, 155.7, 158.7; ^{31}P NMR (162 MHz, $CDCl_3$) δ 149.5, 149.7; HRMS (ESI)**:** *m/z* calcd for $C_{48}H_{61}N_7O_9PS_2$ $[M + H]^+$ 974.3709, found 774.3715.

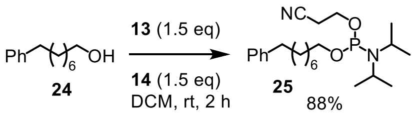

2-Cyanoethyl-8-phenyloctyl-*N*,*N*-diisopropylphosphoramidite (25): The procedure for the synthesis of **3a** was used. After flash column chromatography (SiO_2, 4:1:0.25 hexanes/EtOAc/Et_3N) **25** was afforded as a colorless oil in 88% yield: R_f = 0.3 (4:1 hexanes/EtOAc); ^1H NMR (400 MHz, $CDCl_3$) δ 1.14-1.18 (m, 12H), 1.27-1.33 (m, 6H), 1.55-1.61 (m, 4H), 2.56-2.63 (m, 4H), 3.53-3.63 (m, 4H), 3.74-3.86 (m, 4H), 7.14-7.16 (m, 3H), 7.23-7.25 (m, 2H); ^{13}C NMR (100 MHz, $CDCl_3$) δ 20.6 (d, J_{C-P} = 6.74 Hz), 24.9, 26.2, 29.6 (d, J_{C-P} = 18.72 Hz), 29.5, 29.7, 31.4, 31.5, 31.7, 36.2, 43.2 (d, J_{C-P} = 12.35 Hz), 58.5 (d, J_{C-P} = 18.95 Hz), 63.9 (d, J_{C-P} = 16.97 Hz), 117.8, 125.7, 128.3, 128.5, 143.0; ^{31}P NMR (162 MHz, $CDCl_3$) δ 148.5; HRMS (ESI): *m/z* calcd for $C_{23}H_{40}N_2O_2P$ $[M+H]^+$ 407.2827, found 407.2812.

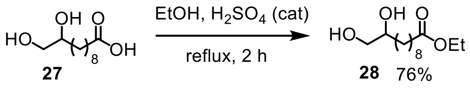

Ethyl 10,11-dihydroxyundecanoate (28): A solution of **27** (2.0 g, 9.17 mmol) and conc. H_2SO_4 (1 mL) in ethanol (100 mL) was stirred at reflux for 2 h. After cooling to rt, the reaction was quenched with 5% Na_2CO_3 (20 mL) and ethanol was evaporated. The remaining material was partitioned between EtOAc (100 mL) and 5% Na_2CO_3 (50 mL). The organic phase was washed with 5% Na_2CO_3 (50 mL × 2), dried over anhydrous Na_2SO_4, filtered and concentrated. Flash column chromatography (SiO_2, 1:1 hexanes/EtOAc) gave **28** as a colorless oil (1.72 g, 76%): R_f = 0.2 (1:1 hexanes/EtOAc); ^1H NMR (400 MHz, CD_3OD): δ 1.22 (t, J = 7.1 Hz, 3H), 1.30 (s, 10H), 1.42-1.50 (m, 2H), 1.54-1.60 (m, 2H), 2.27 (t, J = 7.4 Hz, 2H), 3.28 (bs, 1H), 3.36-3.46 (m, 2H), 3.53 (bs, 1H), 4.08 (q, J = 7.1 Hz, 2H), 4.80 (s, 2H); ^{13}C NMR (100 MHz, CD_3OD): δ 13.3, 24.8, 25.4, 28.9, 29.1, 29.3, 29.5, 33.2, 33.9, 60.1, 66.2, 72.0, 174.3; HRMS (ESI): *m/z* calcd for $C_{13}H_{27}O_4$ $[M + H]^+$ 247.1909, found 247.1907.

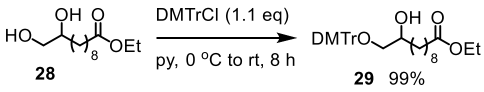

Ethyl 11-(bis(4-methoxyphenyl)(phenyl)methoxy)-10-hydroxyundecanoate (29): The procedure for synthesizing **12** was used. After flash column chromatography (SiO_2, 3:2:0.25 hexanes/EtOAc/Et_3N) **29** was afforded as a yellow oil in 99% yield: R_f = 0.8 (1:1 hexanes/EtOAc); ^1H NMR (400 MHz, $CDCl_3$): δ 1.20-1.28 (m, 10H), 1.35-1.45 (m, 2H), 1.53-1.63 (m, 2H), 2.26 (t, J = 7.3 Hz, 2H), 2.47 (bs, 1H), 2.98-3.04 (m, 1H), 3.13-3.17 (m, 1H), 3.74 (s, 6H), 4.10 (q, J = 7.1 Hz, 2H), 6.81 (d, J = 8.8 Hz, 4H), 7.14-7.19 (m, 2H),

7.26 (t, J = 7.8 Hz, 2H), 7.31 (d, J = 8.8 Hz, 4H), 7.43 (d, J = 5.8 Hz, 2H); ^{13}C NMR (100 MHz, $CDCl_3$): δ 14.4, 25.1, 25.6, 29.31, 29.39, 29.5, 29.7, 33.6, 34.5, 55.3, 60.3, 67.8, 71.1, 86.2, 113.3, 126.9, 128.0, 128.3, 130.2, 136.3, 145.1, 158.6, 174.0; HRMS (ESI): m/z calcd for $C_{34}H_{44}O_6Na$ $[M + Na]^+$ 571.3035, found 571.3031.

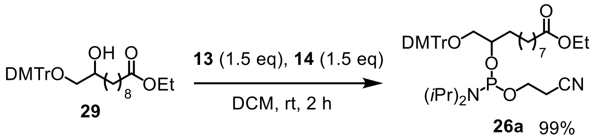

Ethyl 11-(bis(4-methoxyphenyl)(phenyl)methoxy)-10-(((2-cyanoethoxy)(diisopropylamino)phosphanyl)oxy)undecanoate (26a): The procedure for synthesizing **3a** was used. After flash column chromatography (SiO_2, 2:1:0.15 hexanes/EtOAc/Et_3N) **26a** was afforded as a colorless oil in 99% yield: Mixture of diastereoisomers; R_f = 0.6 and 0.7 (1:1 hexanes/EtOAc); ^{1}H NMR (400 MHz, $CDCl_3$): δ 1.05 (d, J = 6.7 Hz, 4H), 1.14-1.26 (m, 22H), 1.54-1.63 (m, 2H), 2.24-2.29 (m, 2H), 2.35-2.39 (m, 1H), 2.59 (t, J = 6.5 Hz, 2H), 2.92-2.99 (m, 1H), 3.09-3.18 (m, 2H), 3.50-3.65 (m, 3H), 3.76 (s, 6H), 3.91-3.99 (m, 1H), 4.11 (q, J = 7.1 Hz, 2H), 6.80 (t, J = 8.7 Hz, 4H), 7.15-7.20 (m, 2H), 7.23-7.27 (m, 2H), 7.32 (d, J = 8.9 Hz, 4H), 7.45 (d, J = 7.0 Hz, 2H); ^{13}C NMR (100 MHz, $CDCl_3$): δ 14.4, 20.3, 20.6, 24.6, 24.7, 24.8, 24.9, 25.0, 25.2, 25.3, 29.3, 29.41, 29.44, 29.5, 29.7, 29.8, 33.6, 33.8, 34.5, 43.1, 43.3, 43.4, 55.3, 58.3, 58.6, 60.3, 66.2, 66.5, 73.6, 73.7, 74.4, 74.6, 86.0, 86.1, 117.9, 126.81, 126.87, 127.8, 128.4, 128.5, 130.2, 130.32, 130.36, 136.5, 145.3, 158.6, 174.0 ppm. ^{31}P NMR (162 MHz, $CDCl_3$): δ 149.1, 149.7; HRMS (ESI): m/z calcd for $C_{43}H_{61}N_2O_7PNa$ $[M + Na]^+$ 771.4114, found 771.4108.

References

[1] Shahsavari, S.; McNamara, C.; Sylvester, M.; Bromley, E.; Joslin, S.; Lu, B. Y.; Fang, S. Y. *Beilstein J. Org. Chem.* **2018**, *14*, 1750-1757. doi:10.3762/bjoc.14.149

[2] Grotli, M.; Douglas, M.; Beijer, B.; Eritja, R.; Sproat, B. *Bioorg. Med. Chem. Lett.* **1997**, *7*, 425-428. doi:10.1016/S0960-894X(97)00024-3

Protocol for ODN cleavage and deprotection

ODN synthesis should be carried out under standard conditions except that capping failure sequences in each synthesis cycle should be achieved using **25** instead of acetic anhydride, and the last nucleotide should be incorporated with 5'-trityl (instead of 5'-DMTr) protected nucleoside phosphoramidite. The deprotection and cleavage of 0.52 µmol crude 20-mer ODN is used for the description of the protocol.

1. Place the CPG containing ODN in a 1.5 mL centrifuge tube. Add 1 mL DBU solution (1:9, v/v, DBU/CH_3CN). Shake gently at rt for 15 min.
2. Spin the tube gently and briefly to bring down the CPG and liquids.
3. Remove the supernatant with a pipette.
4. Wash the CPG with CH_3CN (1 mL × 5).
5. Add $NaIO_4$ solution (0.4 M in 1 mL H_2O). Shake gently at rt for 3 h.
6. Spin the tube and remove the supernatant with a pipette.
7. Wash the CPG with H_2O (1 mL × 4).
8. Add aqueous K_2CO_3 (0.05%, pH 8, 500 µL) and shake at rt for 30 min.
9. Transfer the supernatant to another centrifuge tube.
10. Repeat steps 8–9
11. Concentrate the combined supernatant to ≈100 µL and inject into RP HPLC.
12. Collect the peak of trityl-tagged ODN (≈39 min) and concentrate to dryness.
13. Add 1 mL of 80% AcOH solution and shake gently at rt for 3 h.
14. Concentrate to dryness. Make sure to minimize the presence of residue AcOH.
15. Add ≈100 µL H_2O and inject the solution into RP HPLC.
16. Collect the peak of de-tritylated ODN (≈21 min).
17. Concentrate the fractions to dryness. The residue is pure de-tritylated ODN.
