# Supplementary material for: Electrophilic oligodeoxynucleotide synthesis using dM-Dmoc for amino protection
Source: Beilstein J Org Chem. 2019 May 20;15:1116–28. doi: 10.3762/bjoc.15.108 (PMC6541367; doi:10.3762/bjoc.15.108)
Supplement: File 2 — HPLC profiles, MALDI–TOF MS spectra, UV spectra, and OD260 values of ODNs, and NMR spectra of new compounds. [file Beilstein_J_Org_Chem-15-1116-s002.pdf]

BEILSTEIN JOURNAL OF ORGANIC CHEMISTRY

Supporting Information

for

Electrophilic oligodeoxynucleotide synthesis using dM-Dmoc for amino protection

Shahien Shahsavari, Dhananjani N. A. M. Eriyagama, Bhaskar Halami, Vagarshak Begoyan, Marina Tanasova, Jinsen Chen and Shiyue Fang

Beilstein J. Org. Chem. **2019,** *15,* 1116–1128. doi:10.3762/bjoc.15.108

HPLC profiles, MALDI–TOF MS spectra, UV spectra, and OD_{260} values of ODNs, and NMR spectra of new compounds

License and Terms: This is a supporting information file under the terms of the Creative Commons Attribution License (http://creativecommons.org/licenses/by/4.0). Please note that the reuse, redistribution and reproduction in particular requires that the authors and source are credited.
The license is subject to the *Beilstein Journal of Organic Chemistry* terms and conditions: (https://www.beilstein-journals.org/bjoc)

Table of contents

HPLC profiles of ODNs ………………………………………………………………….... S3–S14

MALDI–TOF MS spectra of ODNs ……………………………………………………………S15–S19

UV spectra and OD_{260} values of ODNs ……………………………………………………S20–S24

NMR spectra ……………………………………………………………………………… S25–S43

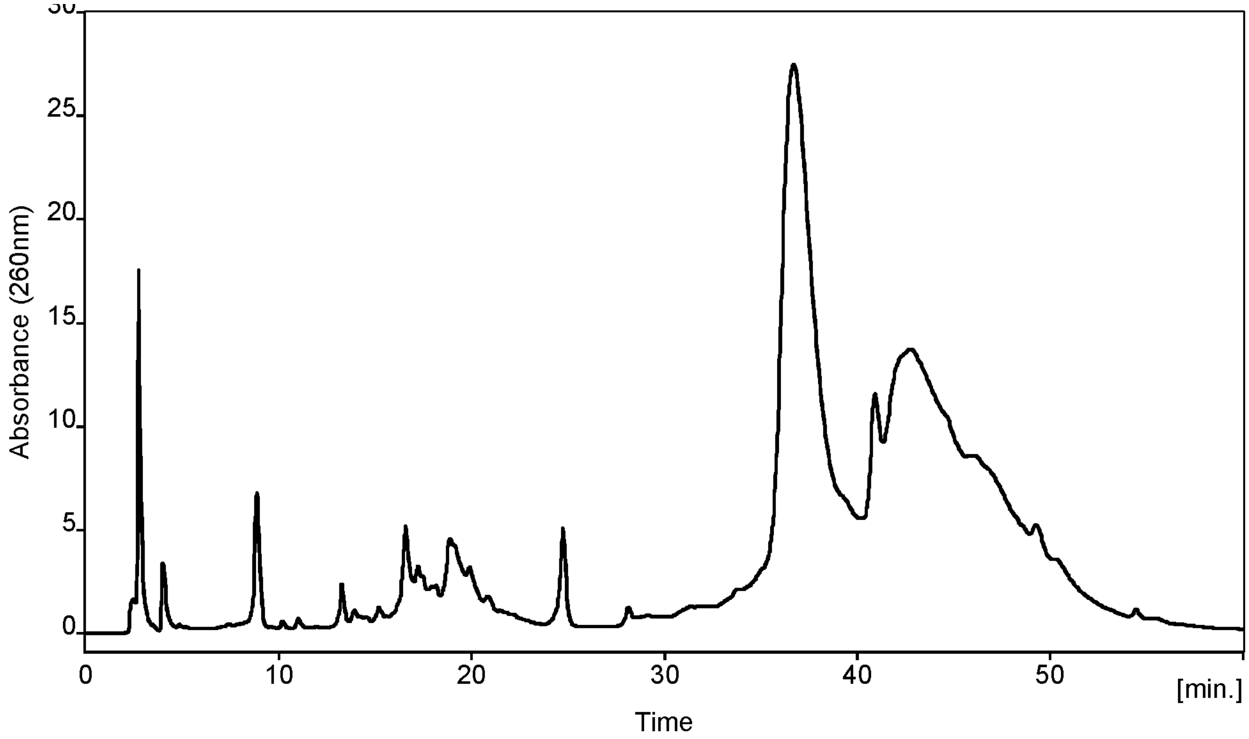

RP HPLC profile of crude trityl-tagged ODN 5'-TTA TCC ACT TCC GTT CTA CT-3' (**30a-tr**). The peak at 35-39 min corresponds to the trityl-tagged ODN. The peaks after 40 min correspond to branched sequences.

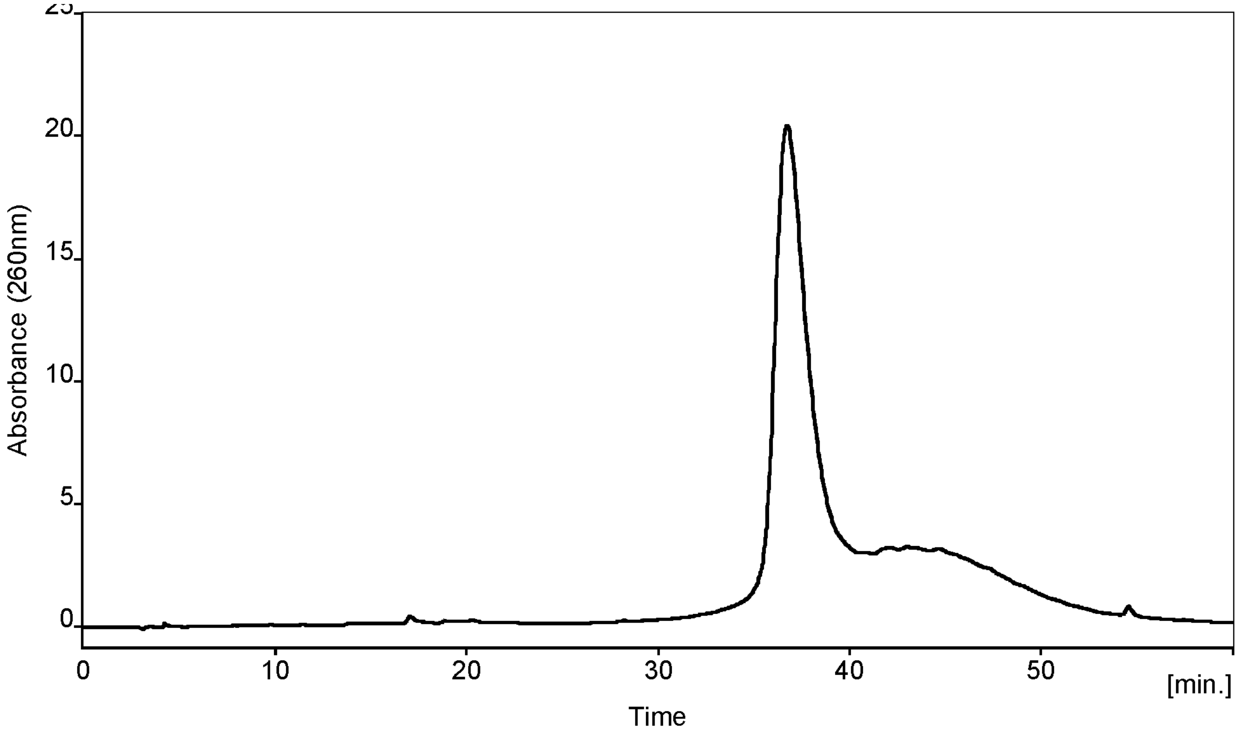

RP HPLC profile of purified trityl-tagged ODN 5'-TTA TCC ACT TCC GTT CTA CT-3' (**30a-tr**).

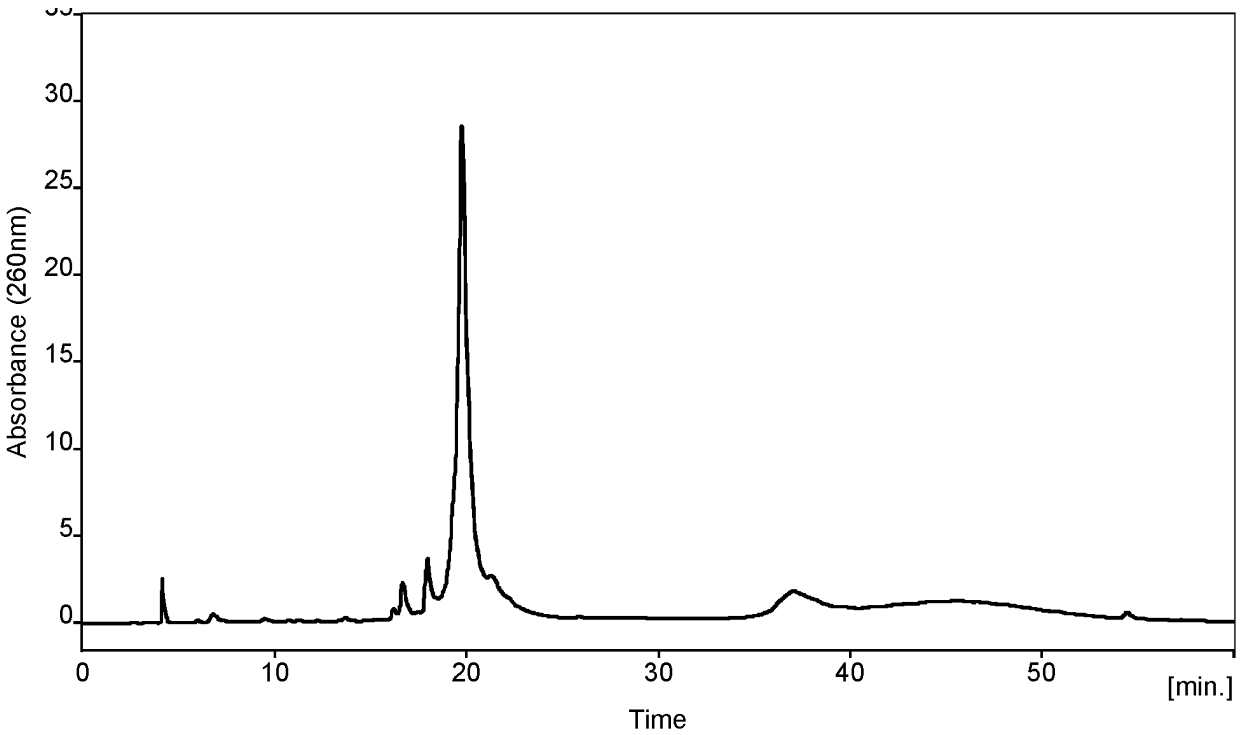

RP HPLC profile of de-tritylated ODN 5'-TTA TCC ACT TCC GTT CTA CT-3' (**30a**).

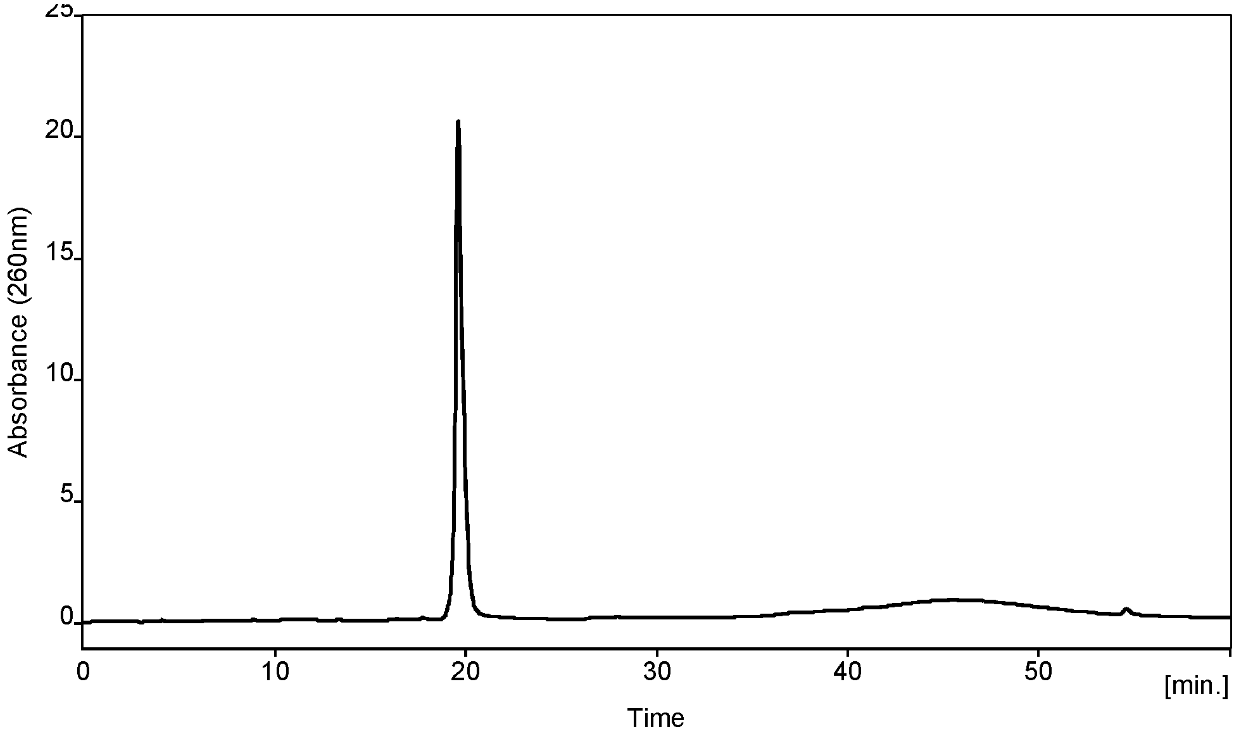

RP HPLC profile of pure de-tritylated ODN 5'-TTA TCC ACT TCC GTT CTA CT-3' (**30a**).

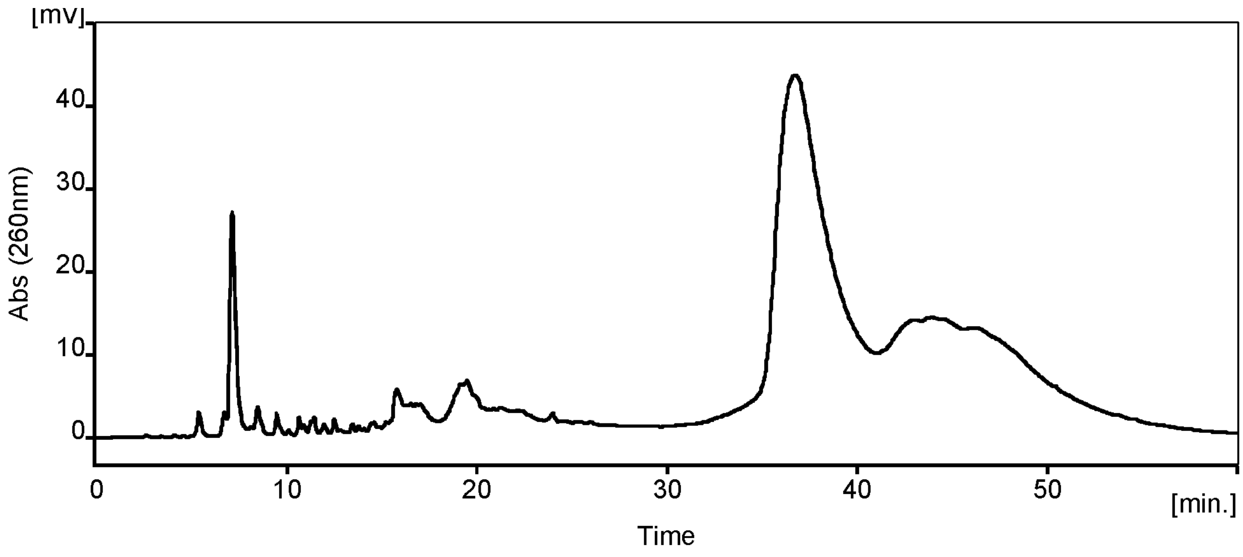

RP HPLC profile of crude trityl-tagged ODN 5'-TTA TCA AAC TTG TAA CCC CT-3' (**30b-tr**). The peak at 35–39 min corresponds to the trityl-tagged ODN. The peaks after 40 min correspond to branched sequences.

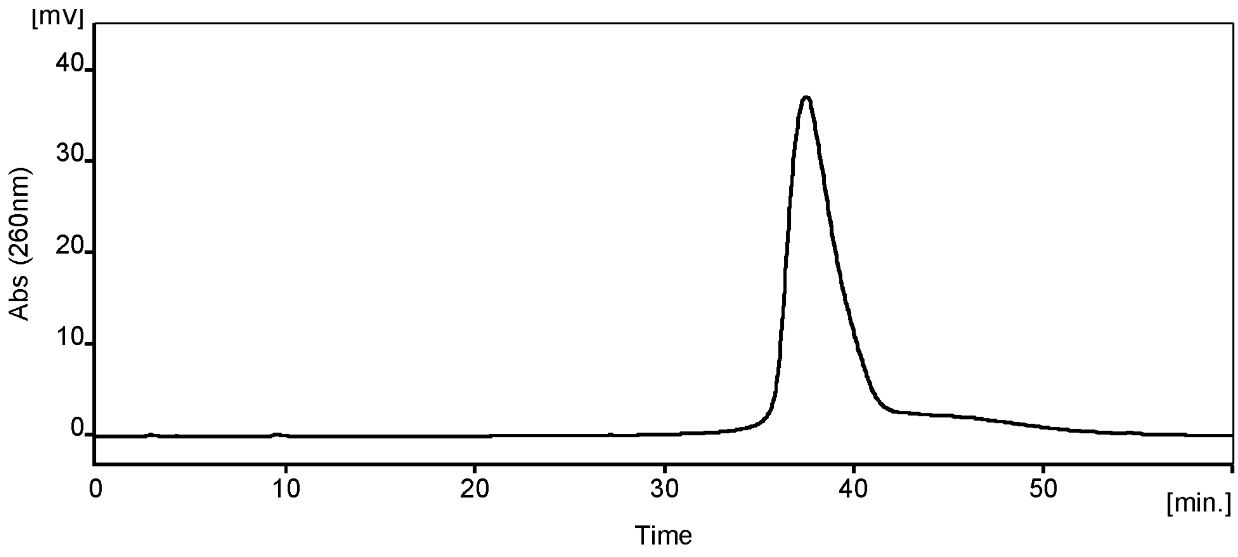

RP HPLC profile of purified trityl-tagged ODN 5'-TTA TCA AAC TTG TAA CCC CT-3' (**30b-tr**).

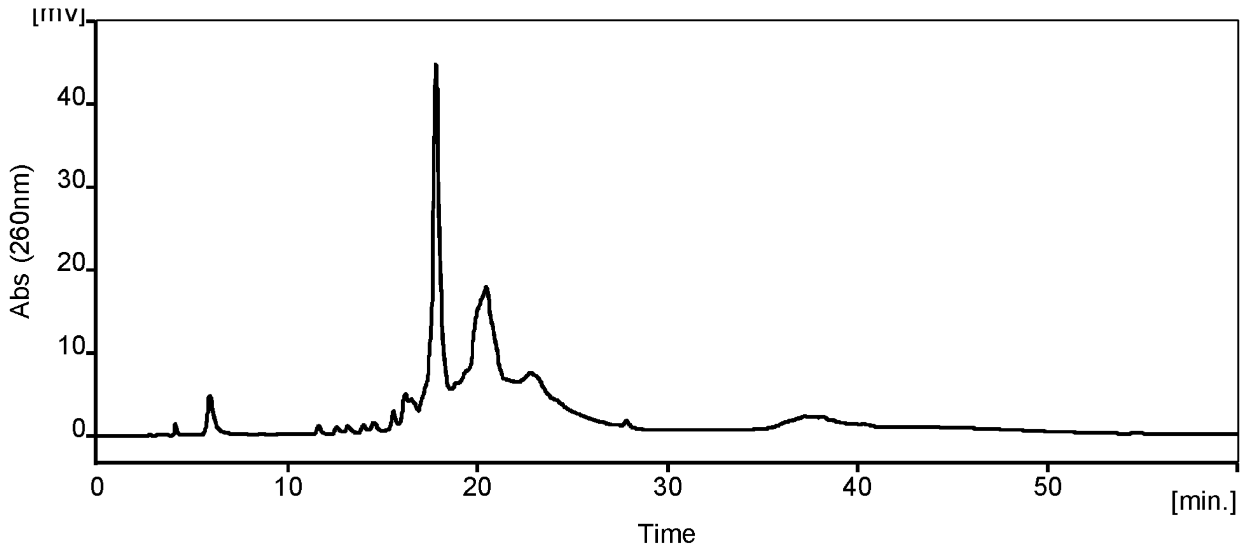

RP HPLC profile of de-tritylated ODN 5'-TTA TCA AAC TTG TAA CCC CT-3' (**30b**).

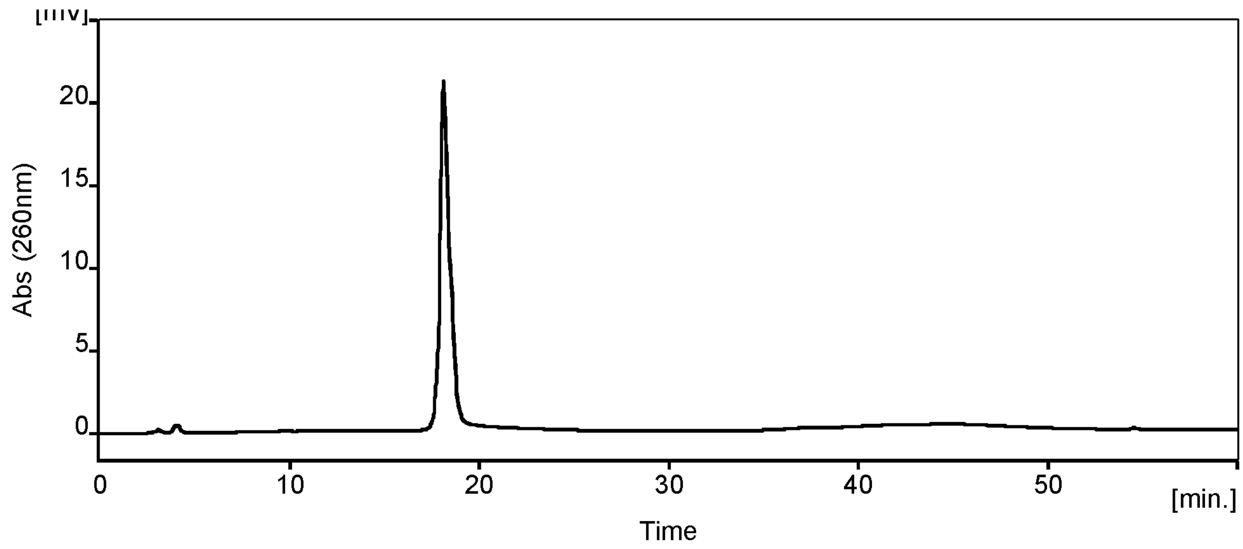

RP HPLC profile of pure de-tritylated ODN 5'-TTA TCA AAC TTG TAA CCC CT-3' (**30b**).

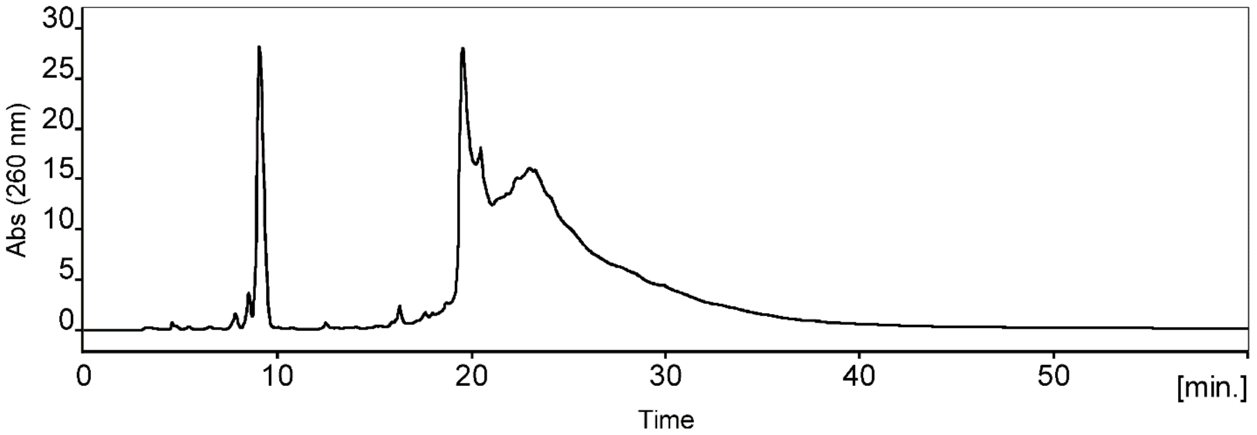

A typical RP HPLC profile of crude ODN (5'-CTA GAT AAC TCA TAG TAC TT-3') synthesized using **3a**–**c** and **4** under standard conditions using acetic anhydride for capping and without 5'-tagging with hydrophobic groups such as trityl and DMTr groups. The peak between 19 and 21 min corresponds to the ODN. The peaks after 21 min correspond to branched sequences. Because the desired ODN and branched sequences were very close, ODN purification was difficult.

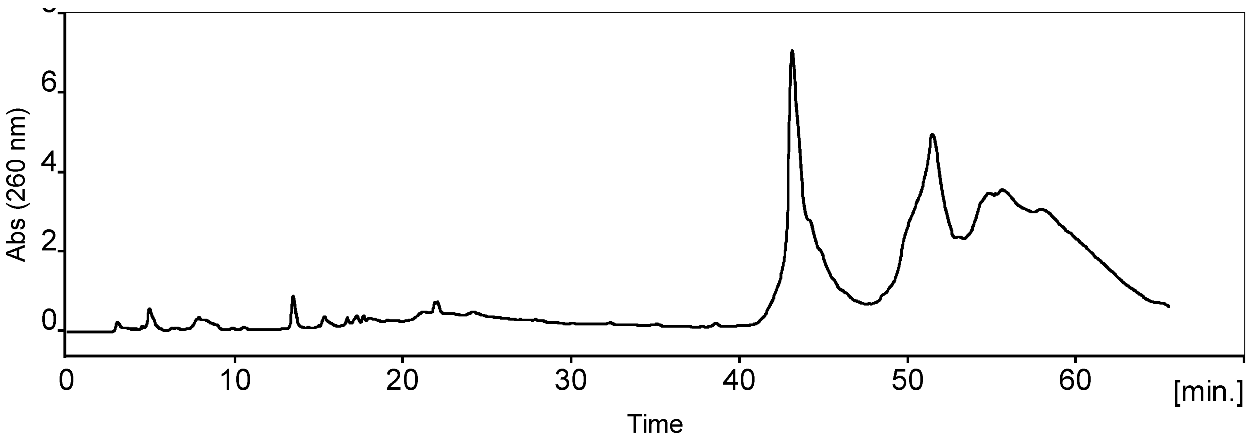

RP HPLC profile of the crude ODN 5'-DMTr-O-TTC CAT CCT AGA AAG CTC AT-3' synthesized using **3a**–**c** and **4** under standard conditions using acetic anhydride for capping. At the end of synthesis, the DMTr group was not removed. Although not always possible, in this case, the DMTr protection survived the cleavage and deprotection conditions involving sodium periodate. The peak in the profile between 43 and 45 min corresponds to the DMTr-tagged ODN. The peaks after 47 min correspond to branched sequences. The branched sequences have longer retention times because they have two or more 5'-ends and thus have two or more DMTr groups.

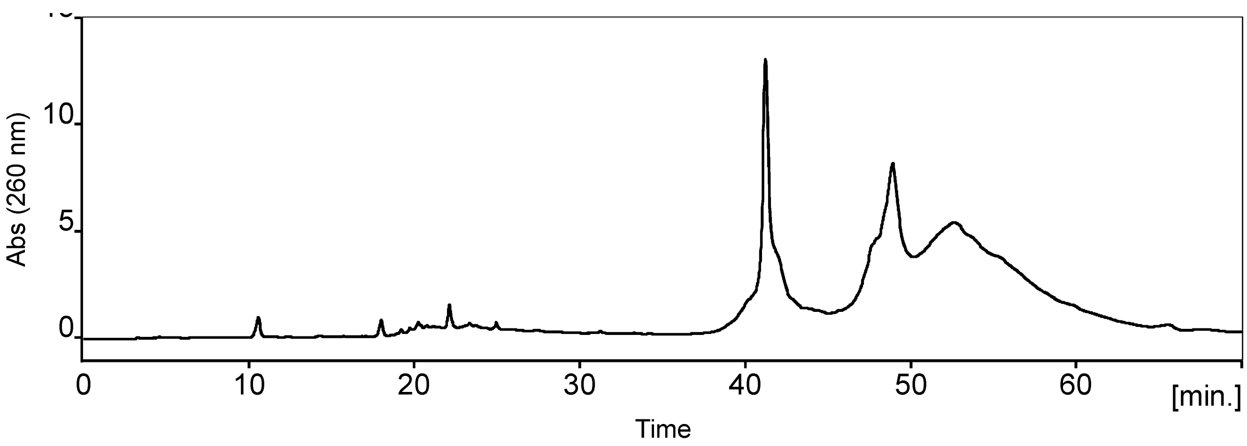

A typical RP HPLC profile of crude ODN (5'-TBDPS-O-CTA GAT AAC TCA TAG TAC TT-3') synthesized using **3a**-**c** and **4** under standard conditions using acetic anhydride for capping and tagged with a TBDPS group at the 5'-end. The TBDPS, which is the *t*-Bu(Ph_2)Si- group, was introduced after solid phase synthesis (5'-DMTr group removed) and before cleavage and deprotection by soaking the CPG in 0.1 M *t*-Bu(Ph_2)SiCl and 0.1 M imidazole in DMF (rt, 12 h). Cleavage and deprotection were then carried out as described in the article. The peak between 41 and 42 min corresponds to the tagged ODN. The peaks after 43 min correspond to branched sequences. The branched sequences have longer retention times because they have two or more 5'-ends and thus have two or more TBDPS groups. The approach separated the desired ODN from the branched sequences very well, but at this stage, we cannot identify a mild condition that is compatible with sensitive modifications on ODNs to remove the TBDPS group after the ODN is purified.

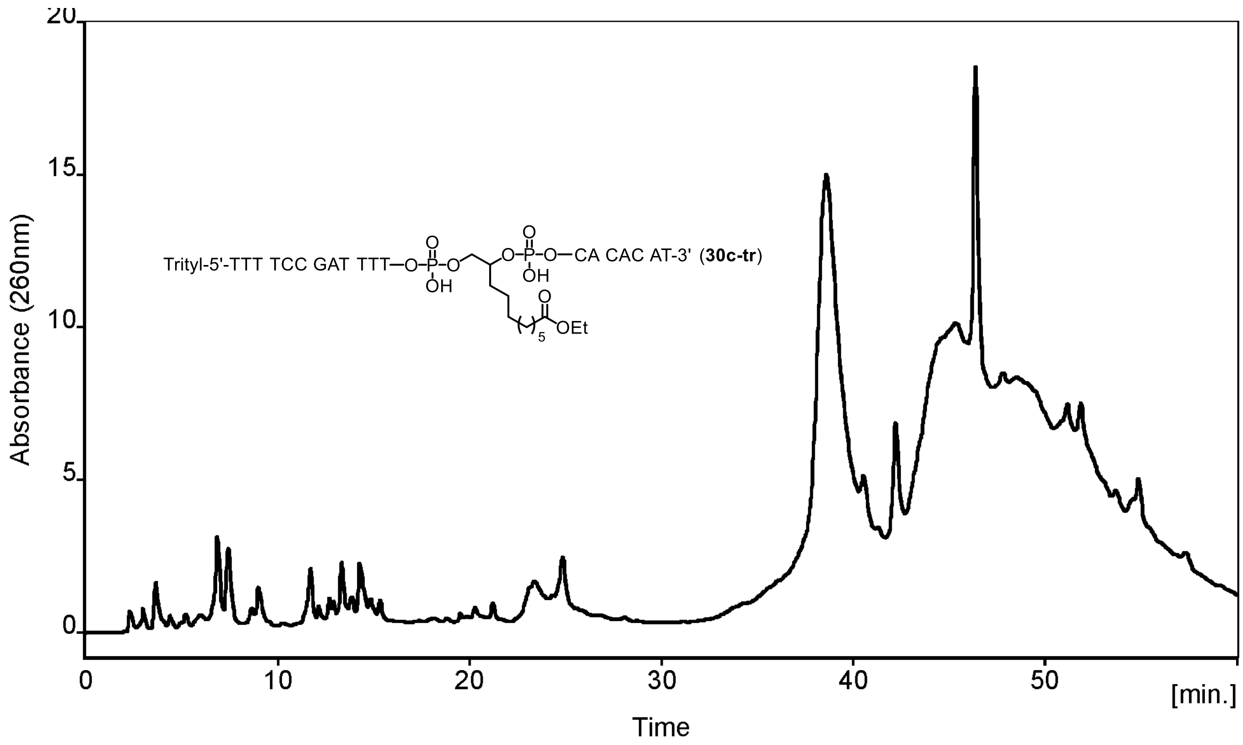

RP HPLC profile of crude trityl-tagged ODN **30c-tr**. The peak at 37–40 min corresponds to the trityl-tagged ODN. The peaks after 40 min correspond to branched sequences.

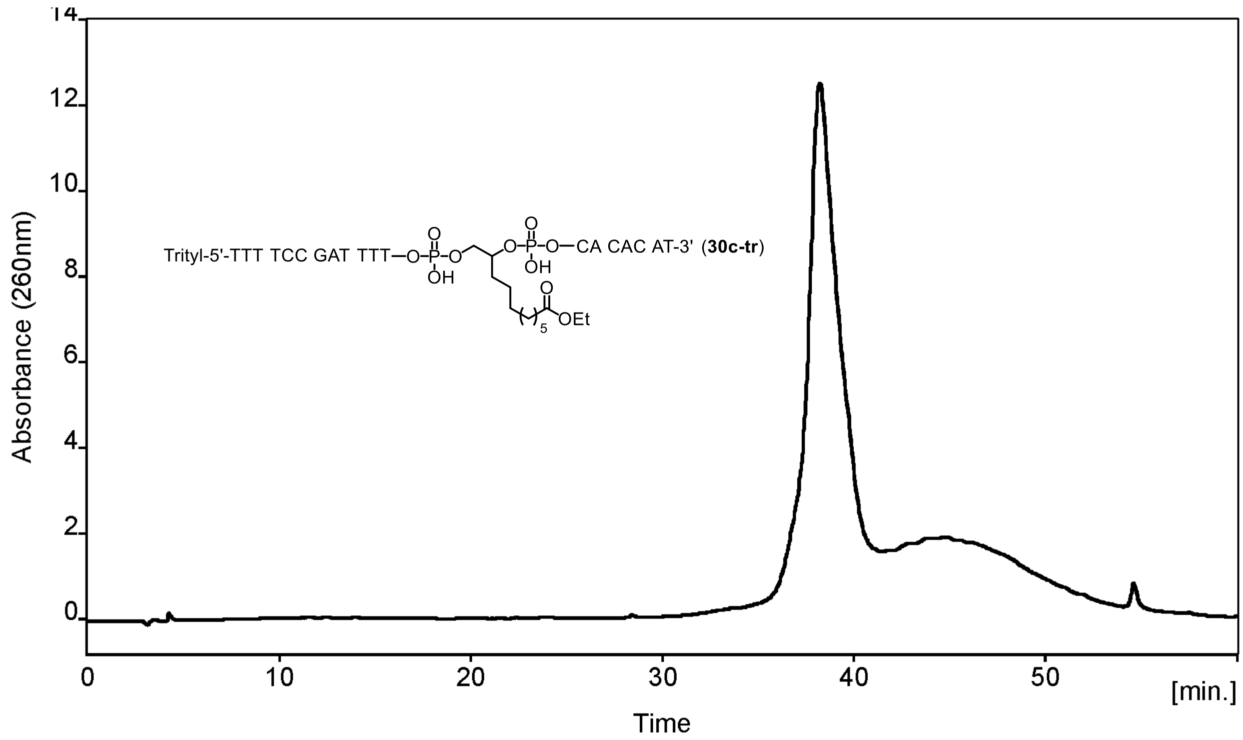

RP HPLC profile of purified trityl-tagged ODN **30c-tr**.

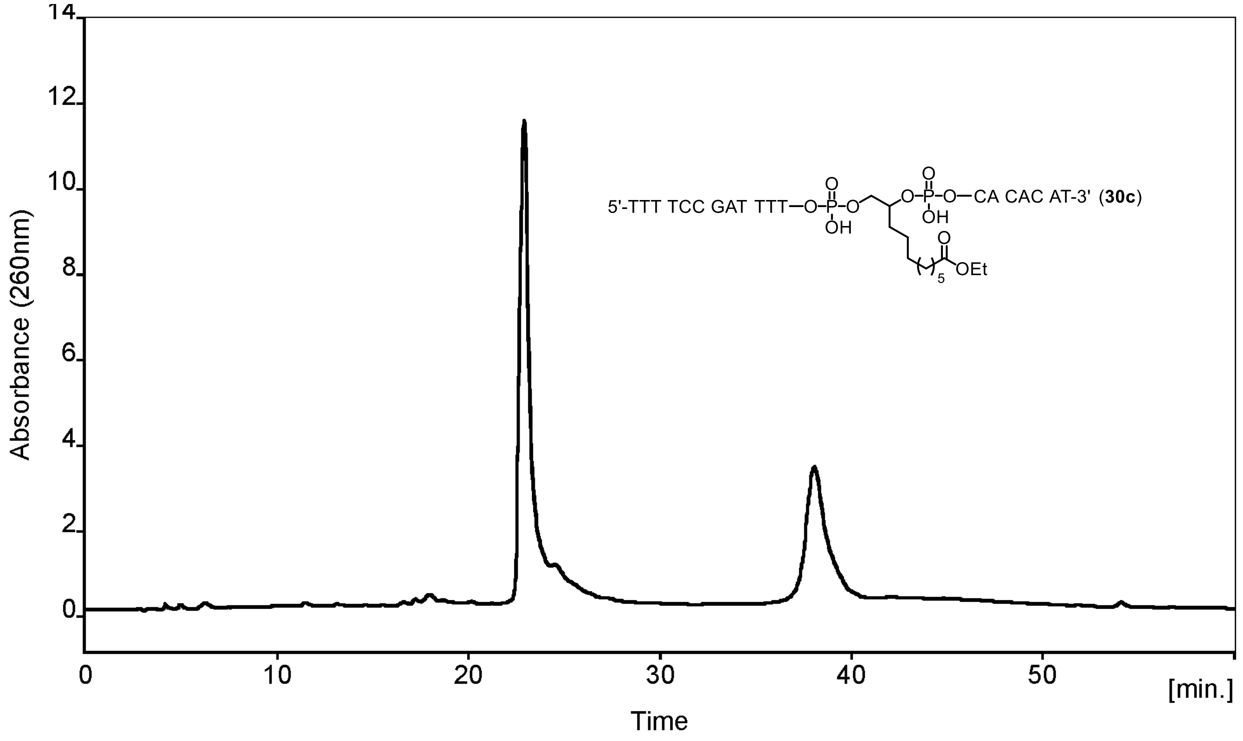

RP HPLC profile of de-tritylated ODN **30c**.

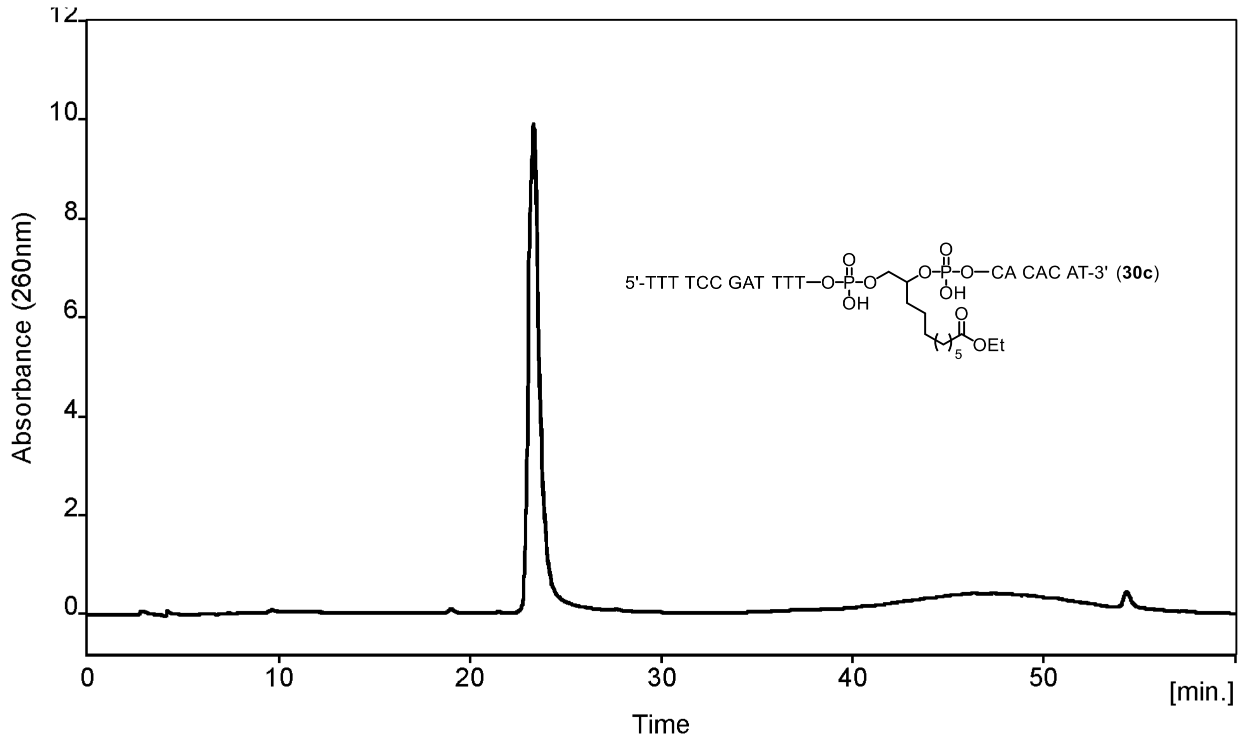

RP HPLC profile of purified de-tritylated ODN **30c**.

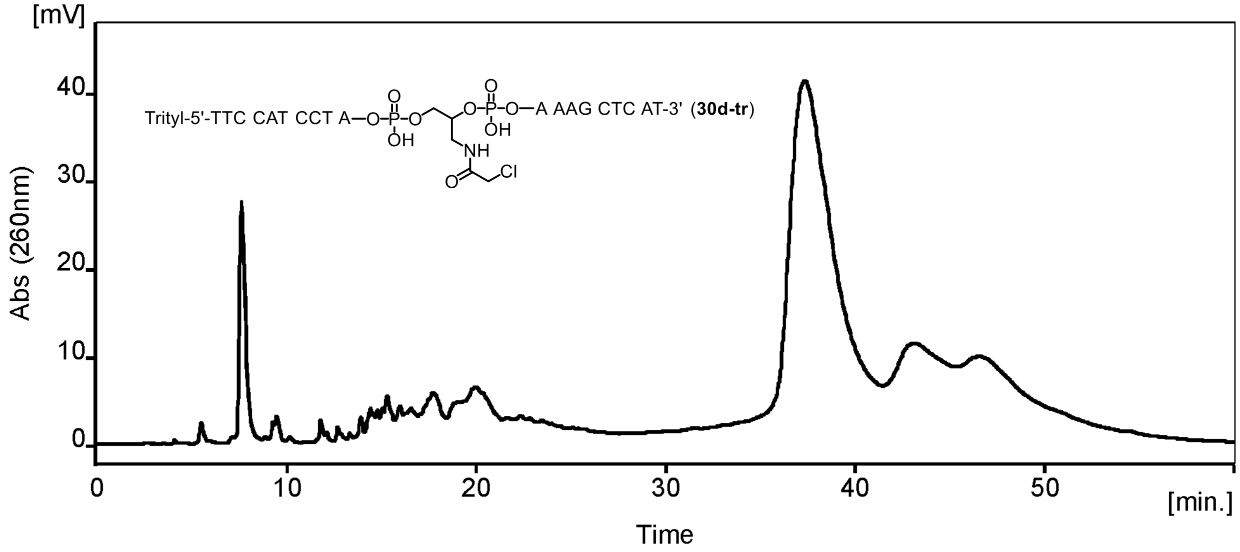

RP HPLC profile of crude trityl-tagged ODN **30d-tr**. The peak at 37–40 min corresponds to the trityl-tagged ODN. The peaks after 40 min correspond to branched sequences.

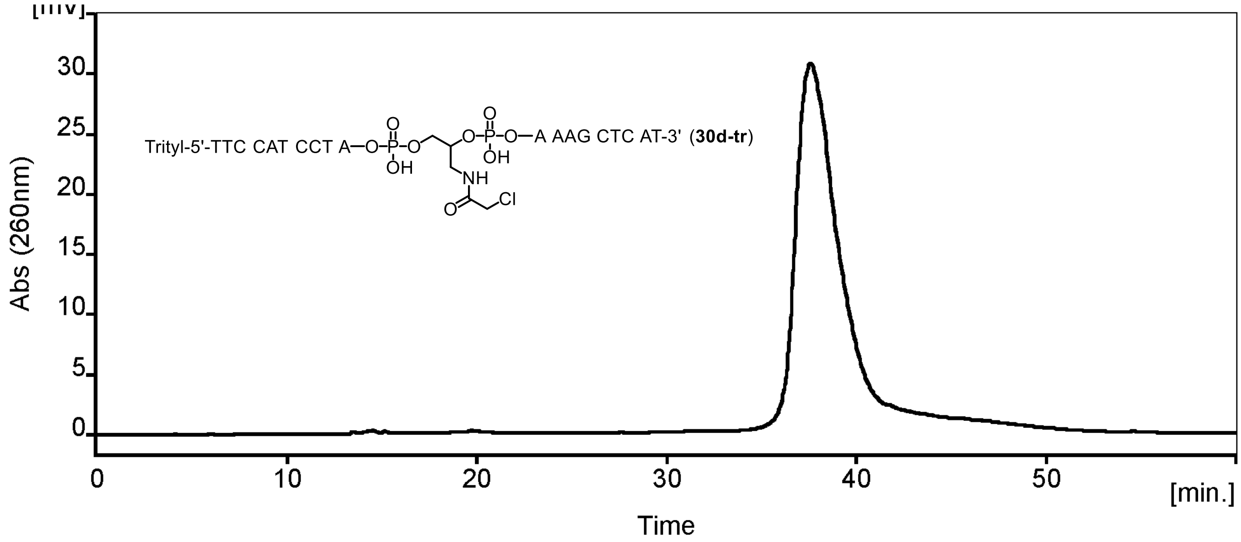

RP HPLC profile of purified trityl-tagged ODN **30d-tr**.

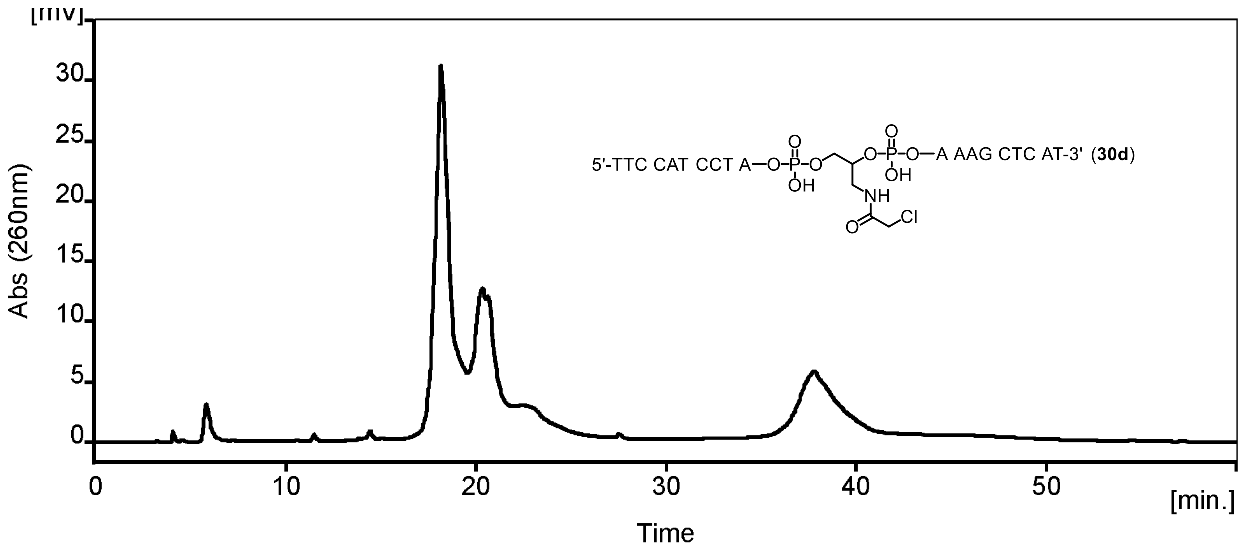

RP HPLC profile of de-tritylated ODN **30d**.

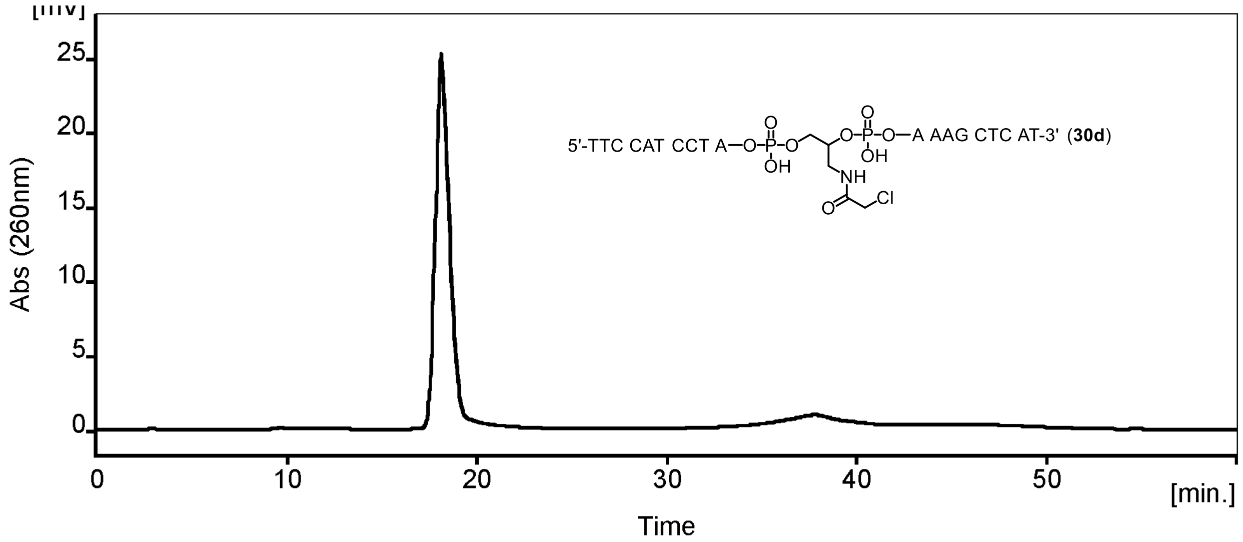

RP HPLC profile of purified de-tritylated ODN **30d**.

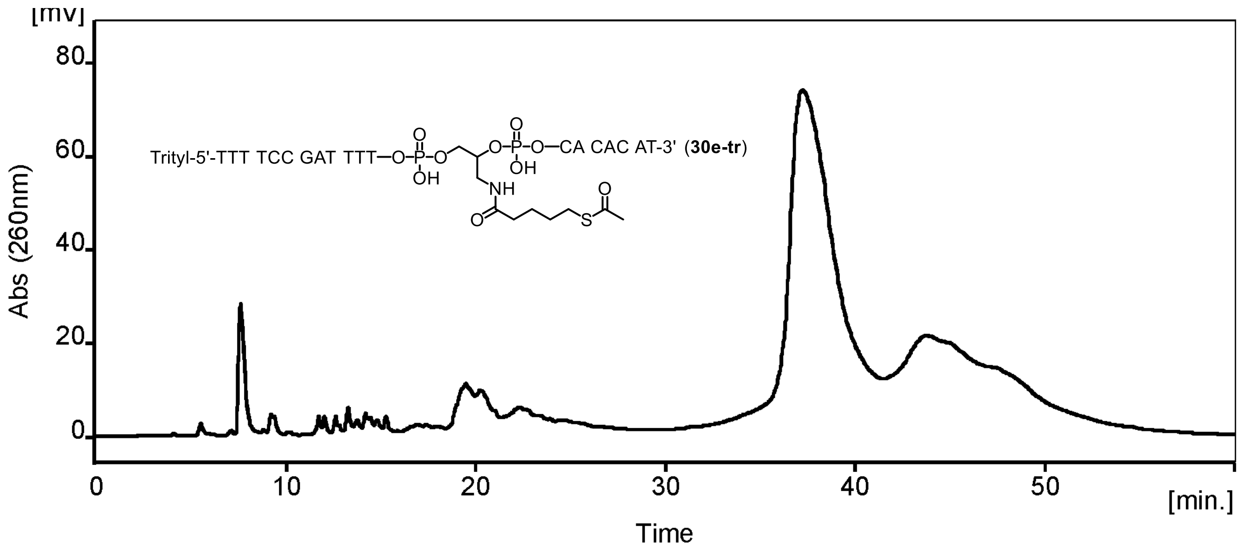

RP HPLC profile of crude trityl-tagged ODN **30e-tr**. The peak at 37–40 min corresponds to the trityl-tagged ODN. The peaks after 40 min correspond to branched sequences.

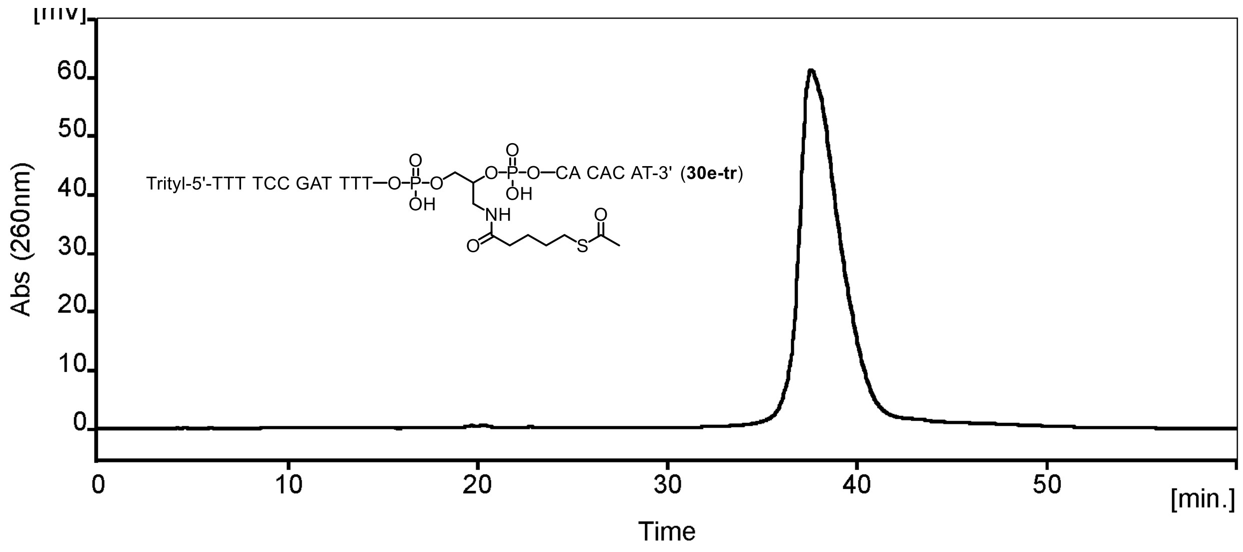

RP HPLC profile of purified trityl-tagged ODN **30e-tr**.

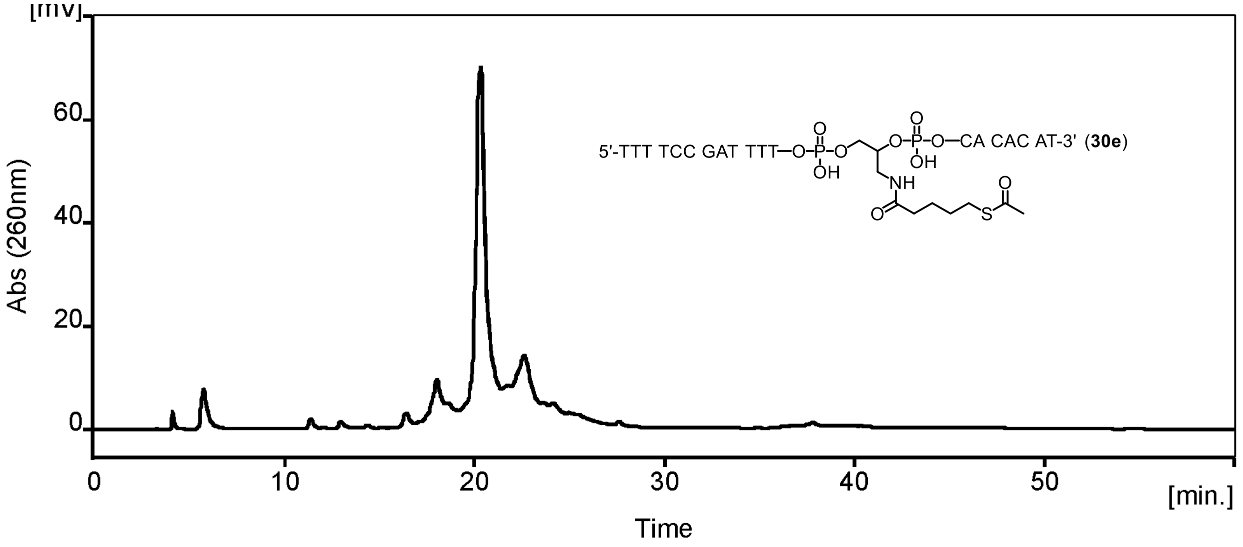

RP HPLC profile of de-tritylated ODN **30e**.

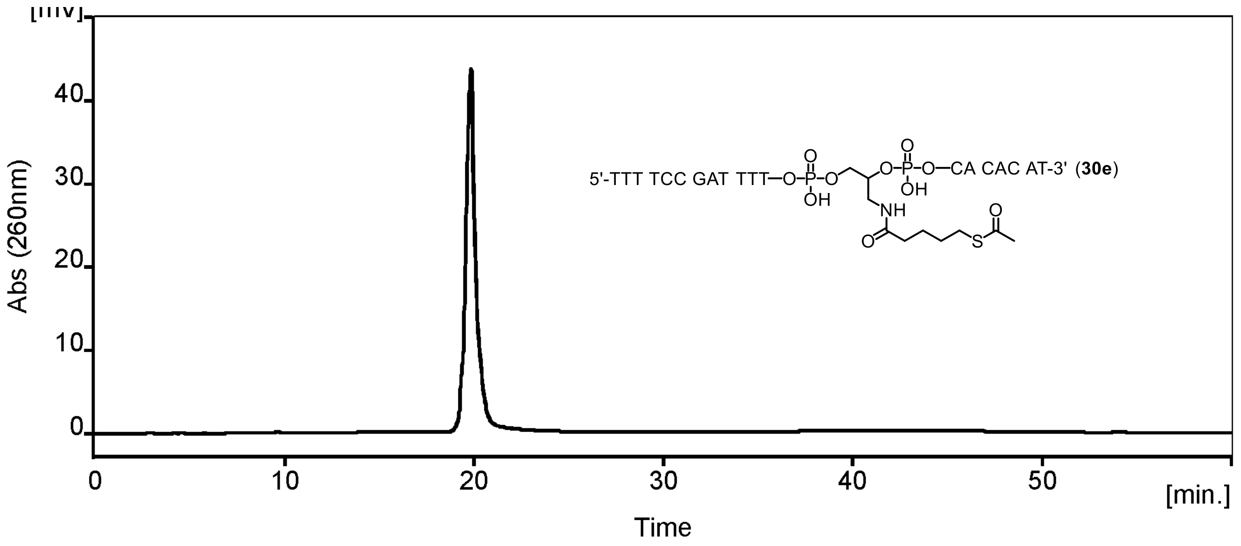

RP HPLC profile of purified de-tritylated ODN **30e**.

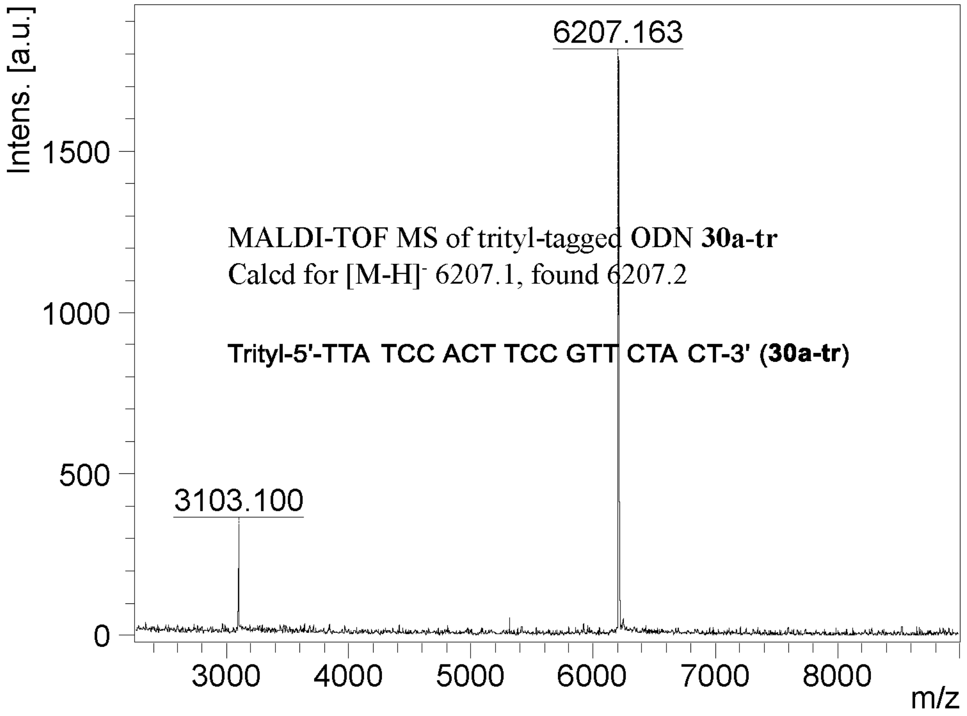

Intens. [a.u.]
6207.163
3103.100
MALDI-TOF MS of trityl-tagged ODN 30a-tr
Calcd for [M-H]- 6207.1, found 6207.2
Trityl-5'-TTA TCC ACT TCC GTT CTA CT-3' (30a-tr)
m/z

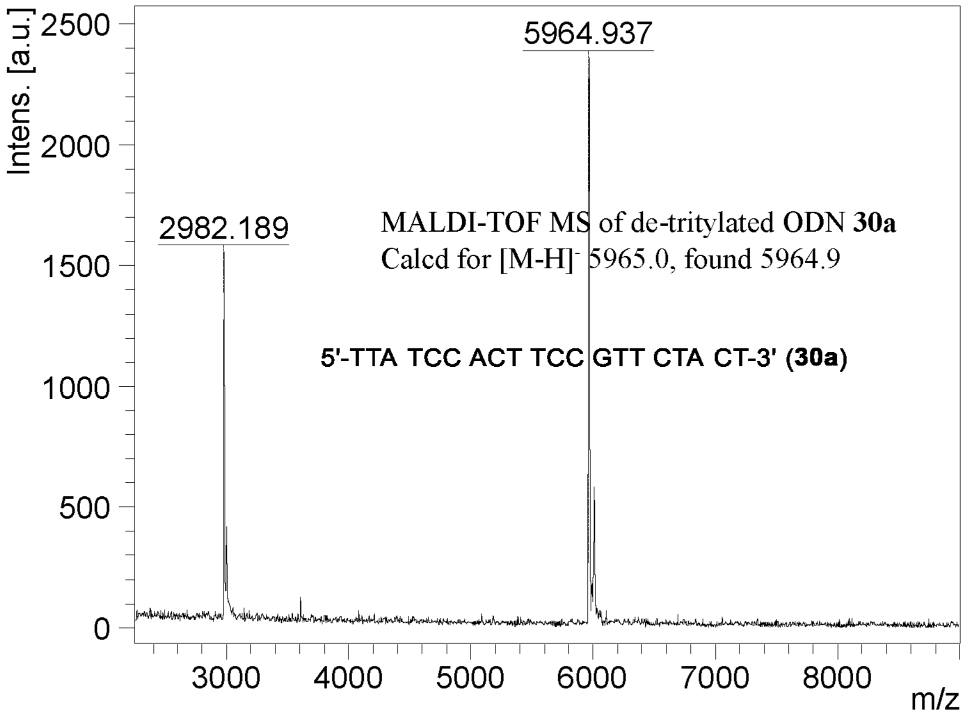

Intens. [a.u.]
5964.937
2982.189
MALDI-TOF MS of de-tritylated ODN 30a
Calcd for [M-H]- 5965.0, found 5964.9
5'-TTA TCC ACT TCC GTT CTA CT-3' (30a)
m/z

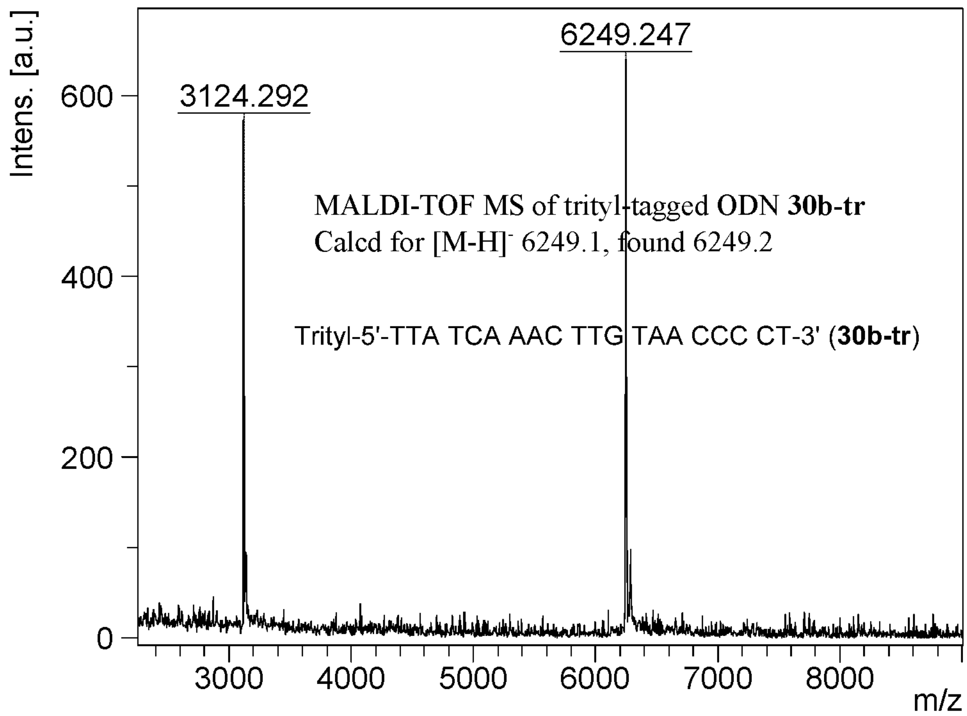
Intens. [a.u.]
600
400
200
0
3124.292
6249.247
MALDI-TOF MS of trityl-tagged ODN **30b-tr**
Calcd for [M-H]$^-$ 6249.1, found 6249.2
Trityl-5'-TTA TCA AAC TTG TAA CCC CT-3' (**30b-tr**)
3000
4000
5000
6000
7000
8000
m/z

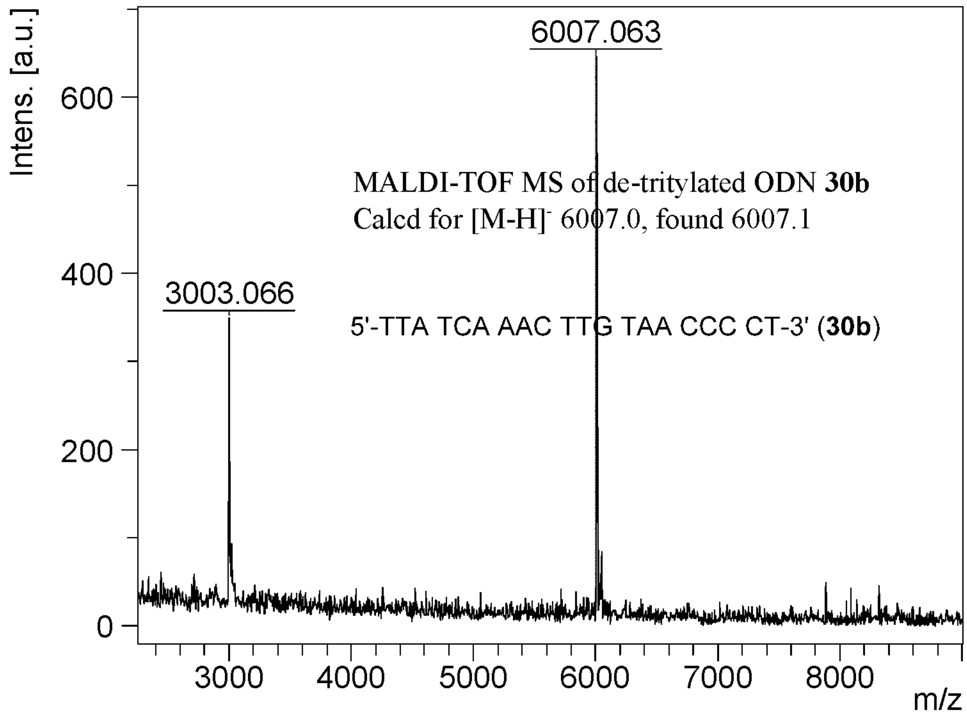
Intens. [a.u.]
600
400
200
0
6007.063
MALDI-TOF MS of de-tritylated ODN **30b**
Calcd for [M-H]$^-$ 6007.0, found 6007.1
3003.066
5'-TTA TCA AAC TTG TAA CCC CT-3' (**30b**)
3000
4000
5000
6000
7000
8000
m/z

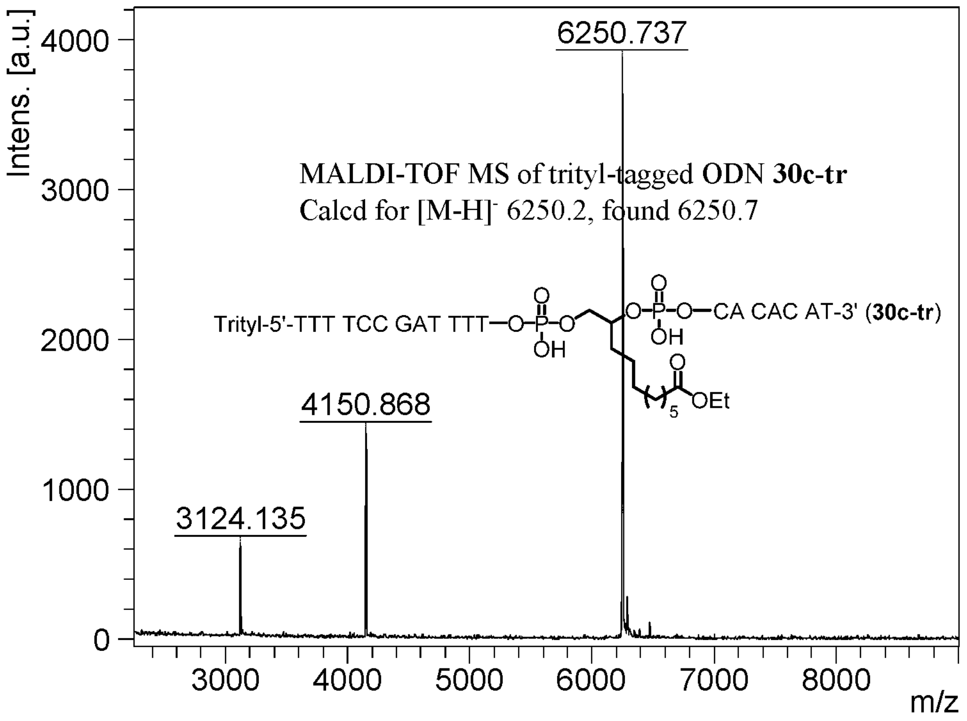
6250.737
4150.868
3124.135
MALDI-TOF MS of trityl-tagged ODN 30c-tr
Calcd for [M-H]- 6250.2, found 6250.7
Trityl-5'-TTT TCC GAT TTT—O-P-O
O-P-O—CA CAC AT-3' (30c-tr)
OH
OEt
Intens. [a.u.]
m/z

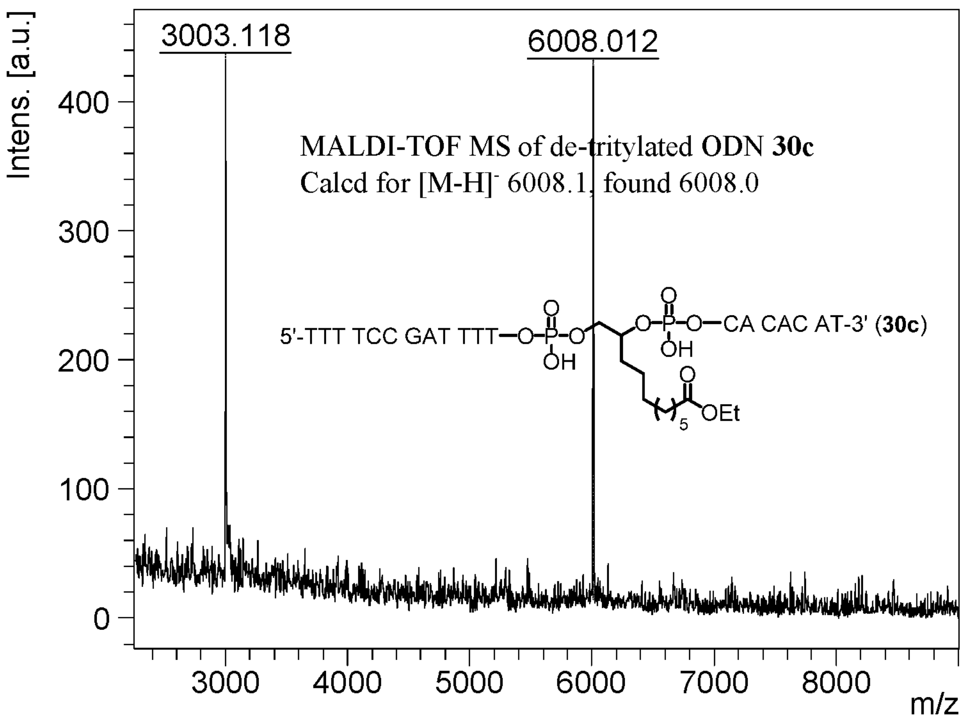
3003.118
6008.012
MALDI-TOF MS of de-tritylated ODN 30c
Calcd for [M-H]- 6008.1, found 6008.0
5'-TTT TCC GAT TTT—O-P-O
O-P-O—CA CAC AT-3' (30c)
OH
OEt
Intens. [a.u.]
m/z

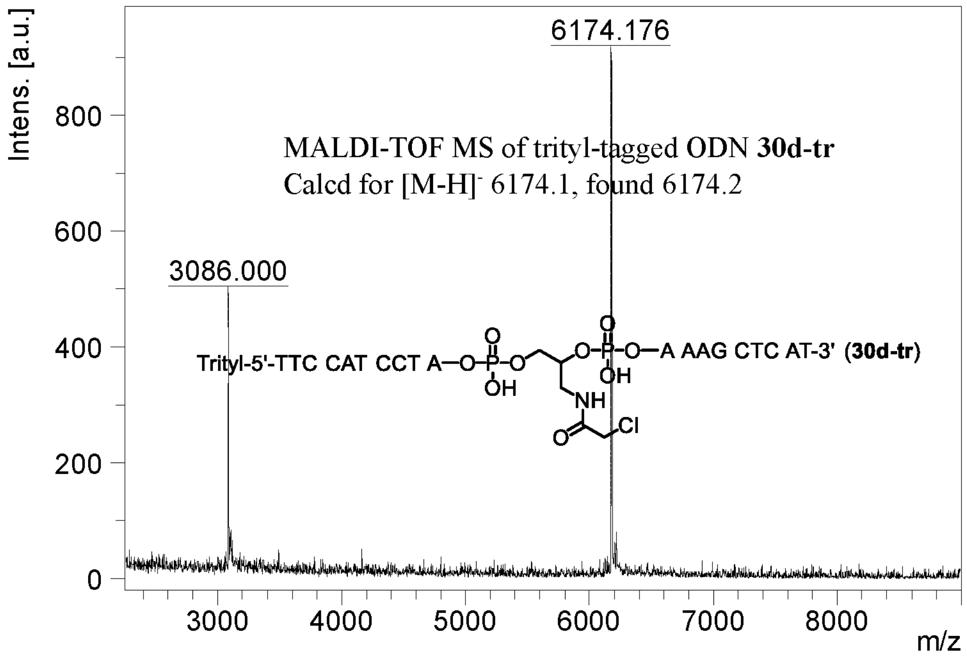

Intens. [a.u.]
6174.176
MALDI-TOF MS of trityl-tagged ODN 30d-tr
Calcd for [M-H]- 6174.1, found 6174.2
3086.000
Trityl-5'-TTC CAT CCT A—O-P-O ... O-P-O—A AAG CTC AT-3' (30d-tr)
OH
NH
Cl
800
600
400
200
0
3000
4000
5000
6000
7000
8000
m/z

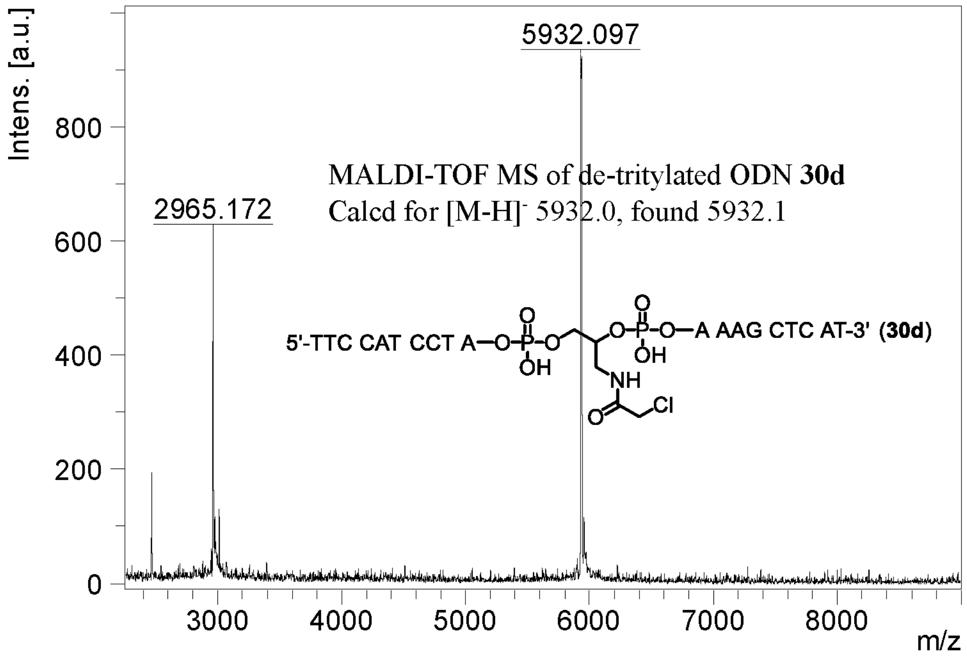

Intens. [a.u.]
5932.097
MALDI-TOF MS of de-tritylated ODN 30d
Calcd for [M-H]- 5932.0, found 5932.1
2965.172
5'-TTC CAT CCT A—O-P-O ... O-P-O—A AAG CTC AT-3' (30d)
OH
NH
Cl
800
600
400
200
0
3000
4000
5000
6000
7000
8000
m/z

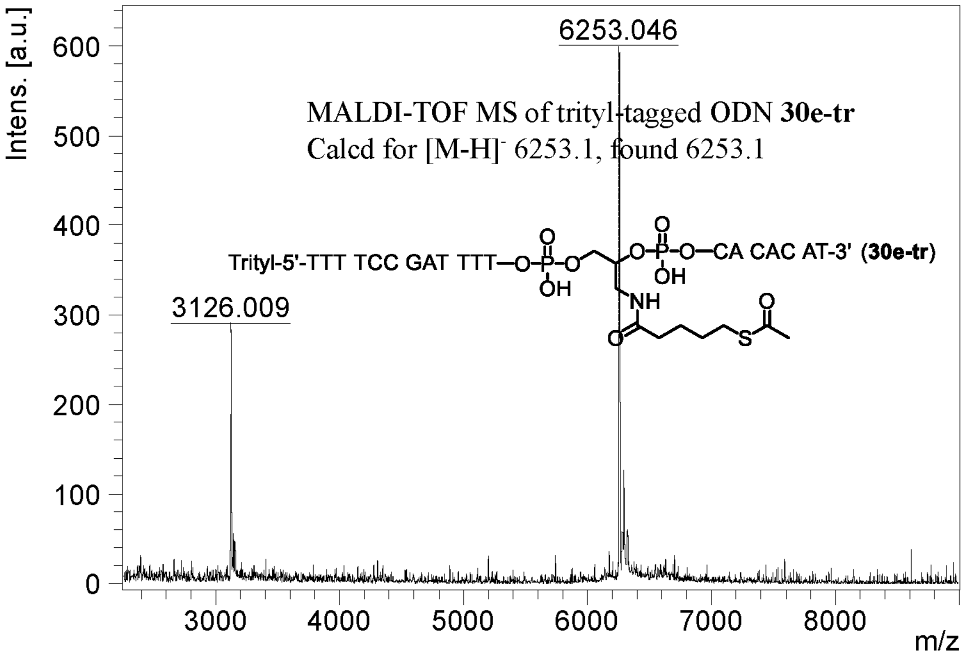
6253.046
MALDI-TOF MS of trityl-tagged ODN 30e-tr
Calcd for [M-H]- 6253.1, found 6253.1
Trityl-5'-TTT TCC GAT TTT—O-P-O
CA CAC AT-3' (30e-tr)
3126.009
Intens. [a.u.]
m/z

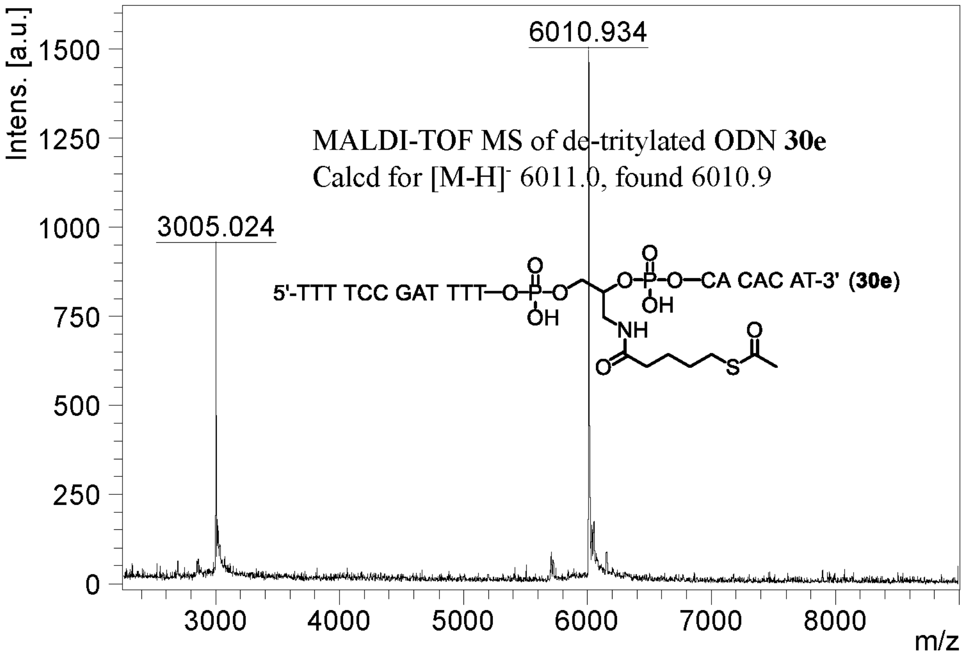
6010.934
MALDI-TOF MS of de-tritylated ODN 30e
Calcd for [M-H]- 6011.0, found 6010.9
3005.024
5'-TTT TCC GAT TTT—O-P-O
CA CAC AT-3' (30e)
Intens. [a.u.]
m/z

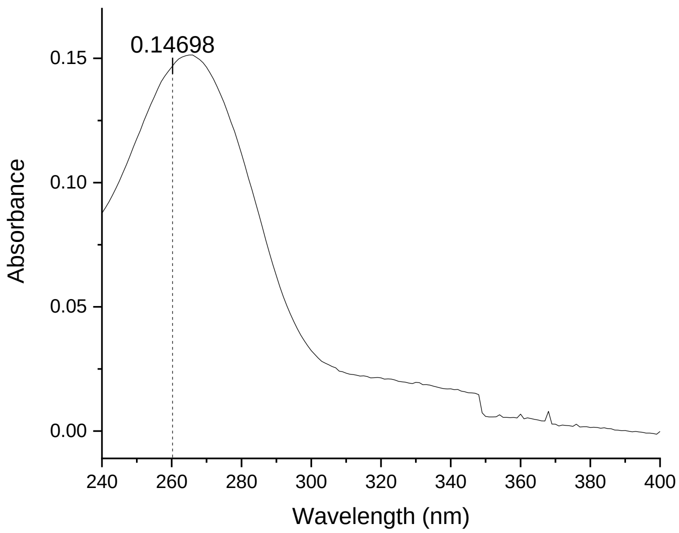

UV of ODN **30a**.

CPG (**4**, loading 26 µmol/g, 20 mg) of 0.52 µmol synthesis was divided into 10 portions. One portion was deprotected and cleaved under non-nucleophilic conditions as described in the experimental section. After HPLC purification, the ODN was dissolved in 2 mL water and the above UV spectrum was measured. Thus, the OD_{260} of the ODN obtained from the 0.52 µmol synthesis is 2.94 (0.147 × 20), which corresponds to a 3.1% overall yield.

For comparing with standard technology, **30a** was synthesized on the same amount of CPG **4** (loading 26 µmol/g, 20 mg) using commercial phosphoramidites under synthesizer manufacturer recommended conditions. The CPG was divided into 10 portions, one portion was deprotected and cleaved with concentrated NH_4OH (55℃, 12 h). After HPLC purification, the OD_{260} was measured the same way and found to be 8.30 (0.415 × 20), which corresponds to a 8.8% overall yield. As can be seen, even though significant amount branched sequences were observed in the HPLC profile of crude **30a** synthesized with the dM-Dmoc technology, the yield of pure target ODN was not significantly lower than that obtained with standard technology. In addition, it is important to note that the standard technology cannot be used to synthesize electrophilic ODNs such as **30c**–**e**.

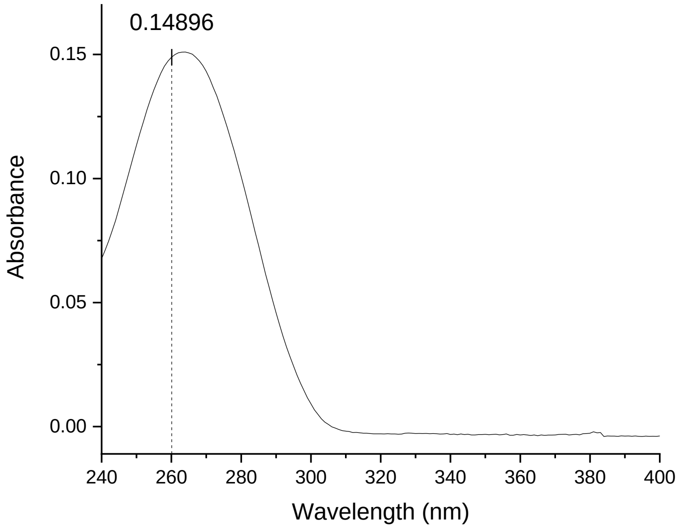

UV of ODN **30b**.

CPG (**4**, loading 26 µmol/g, 20 mg) of 0.52 µmol synthesis was divided into 10 portions. One portion was deprotected and cleaved under non-nucleophilic conditions as described in the experimental section. After HPLC purification, the ODN was dissolved in 2 mL water and the above UV spectrum was measured. Thus, the OD_{260} of the ODN obtained from the 0.52 µmol synthesis is 2.98 (0.149 × 20), which corresponds to a 2.8% overall yield.

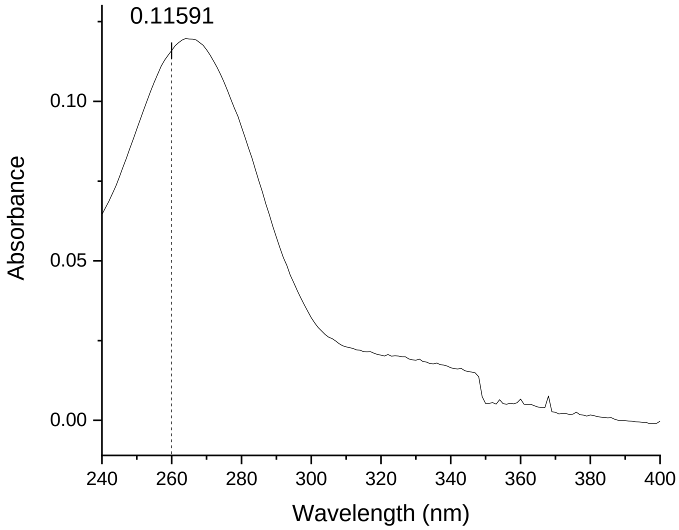

UV of ODN **30c**.

CPG (**4**, loading 26 µmol/g, 20 mg) of 0.52 µmol synthesis was divided into 10 portions. One portion was deprotected and cleaved under non-nucleophilic conditions as described in the experimental section. After HPLC purification, the ODN was dissolved in 2 mL water and the above UV spectrum was measured. Thus, the OD_{260} of the ODN obtained from the 0.52 µmol synthesis is 2.32 (0.116 × 20), which corresponds to a 2.4% overall yield (UV absorption of the unnatural portion of the ODN is not included in the calculation).

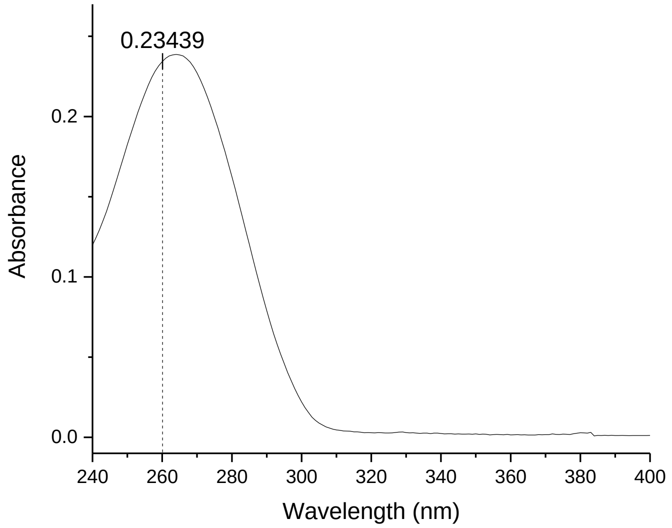

UV of ODN **30d**.

CPG (**4**, loading 26 µmol/g, 20 mg) of 0.52 µmol synthesis was divided into 10 portions. One portion was deprotected and cleaved under non-nucleophilic conditions as described in the experimental section. After HPLC purification, the ODN was dissolved in 2 mL water and the above UV spectrum was measured. Thus, the OD_{260} of the ODN obtained from the 0.52 µmol synthesis is 4.68 (0.234 × 20), which corresponds to a 4.6% overall yield (UV absorption of the unnatural portion of the ODN is not included in the calculation).

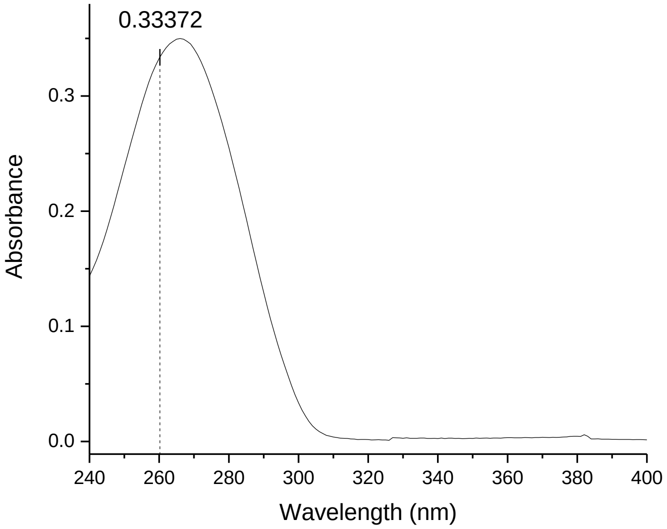

UV of ODN **30e**.

CPG (**4**, loading 26 µmol/g, 20 mg) of 0.52 µmol synthesis was divided into 10 portions. One portion was deprotected and cleaved under non-nucleophilic conditions as described in the experimental section. After HPLC purification, the ODN was dissolved in 2 mL water and the above UV spectrum was measured. Thus, the OD_{260} of the ODN obtained from the 0.52 µmol synthesis is 6.68 (0.334 × 20), which corresponds to a 7.0% overall yield (UV absorption of the unnatural portion of the ODN is not included in the calculation).

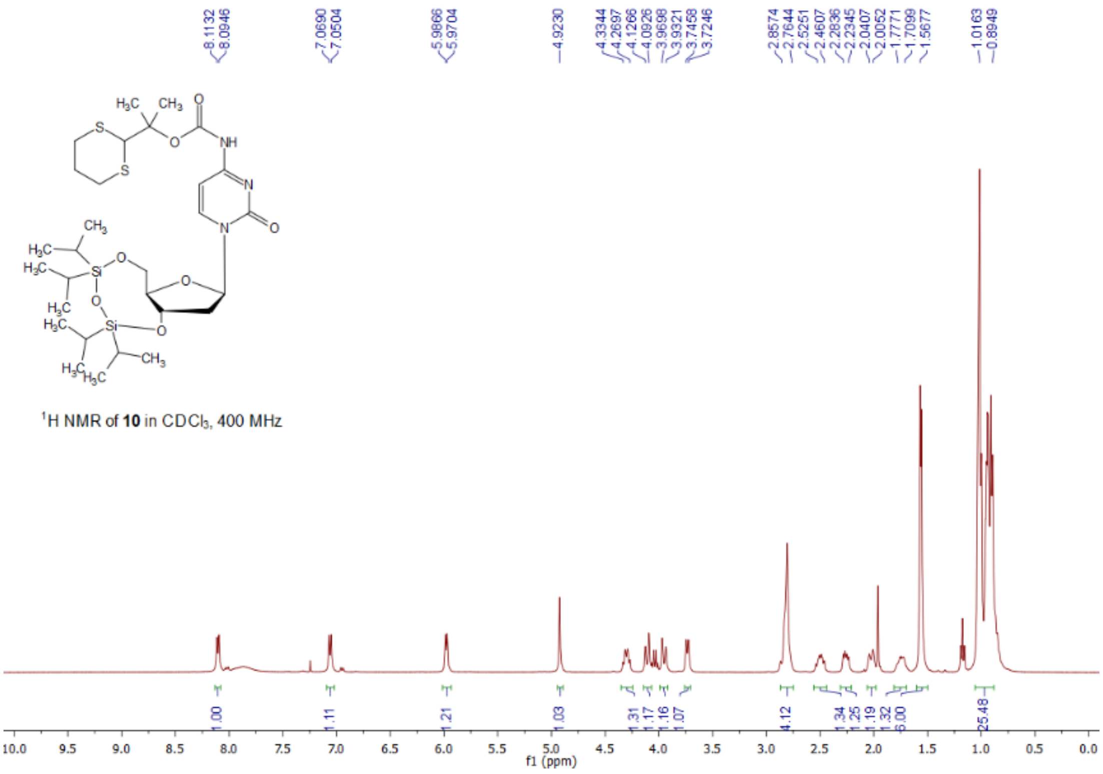

^{1}H NMR of **10** in $CDCl_3$, 400 MHz

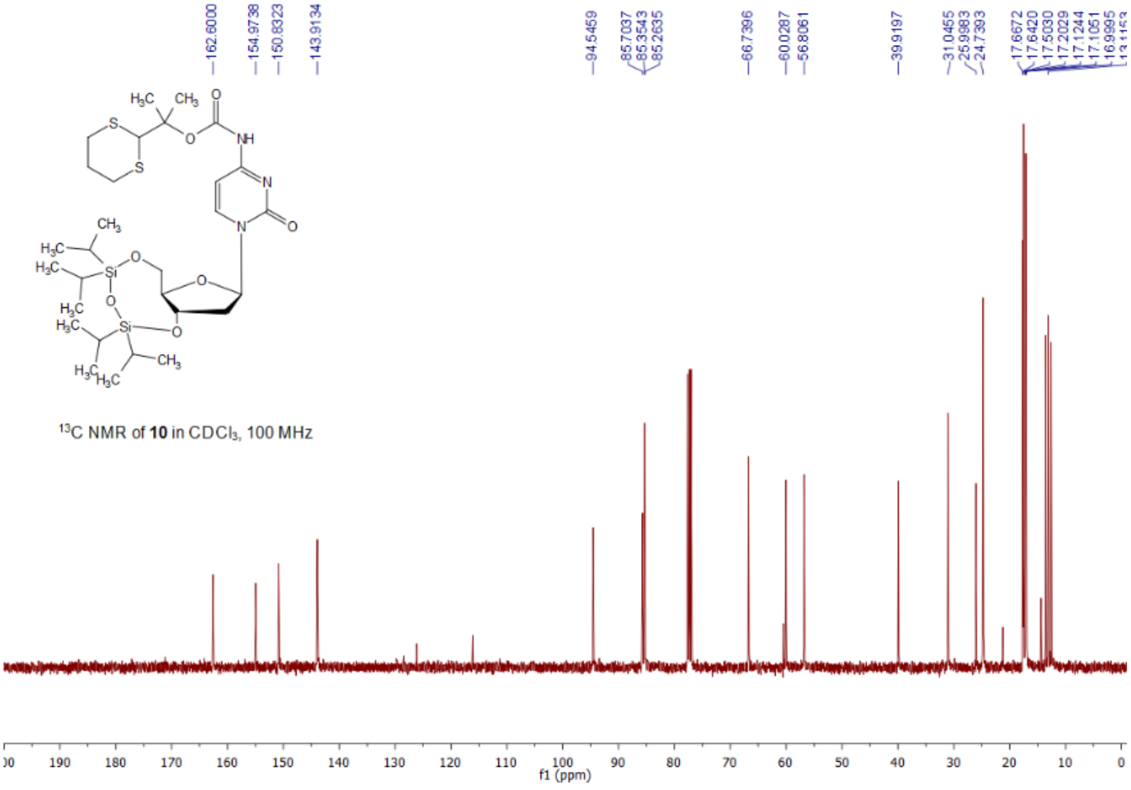

^{13}C NMR of **10** in $CDCl_3$, 100 MHz

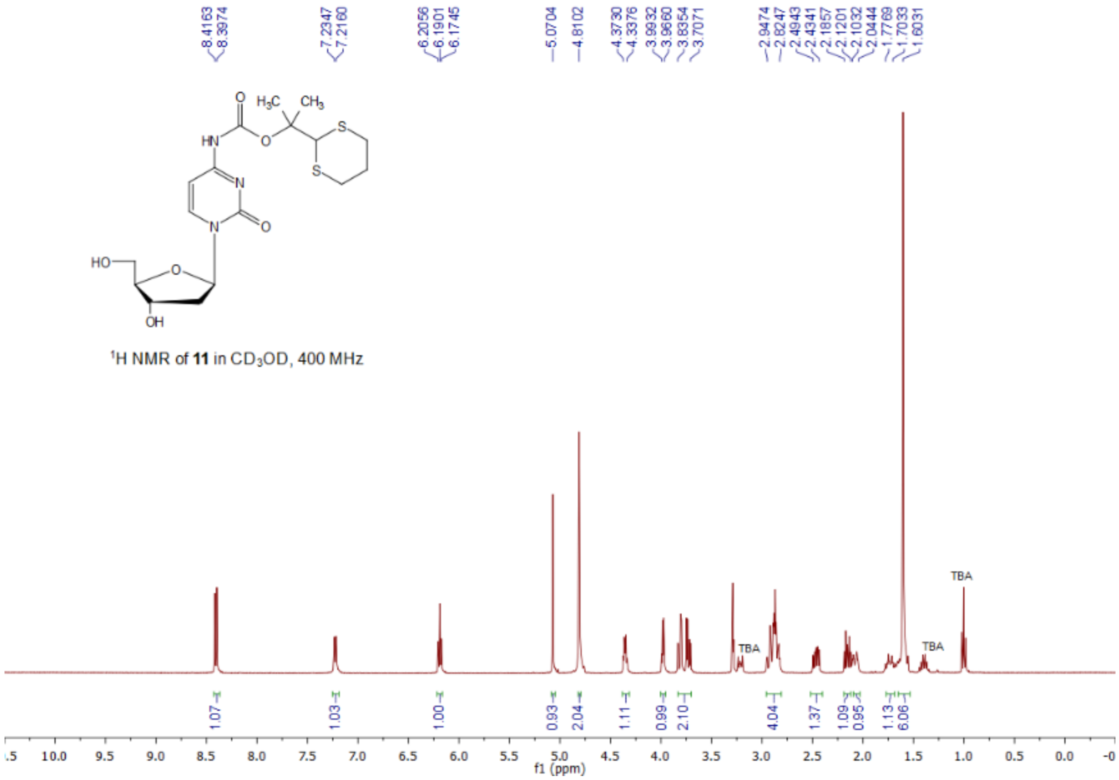

^{1}H NMR of **11** in CD_3OD, 400 MHz

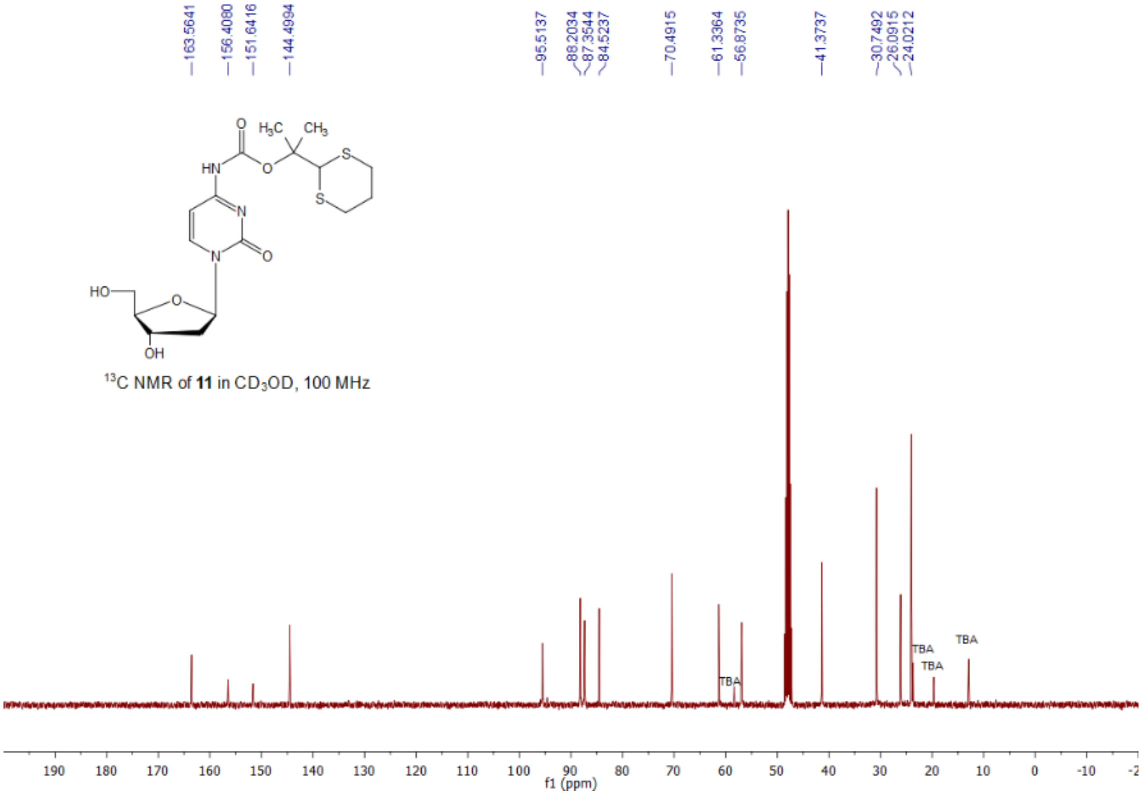

^{13}C NMR of **11** in CD_3OD, 100 MHz

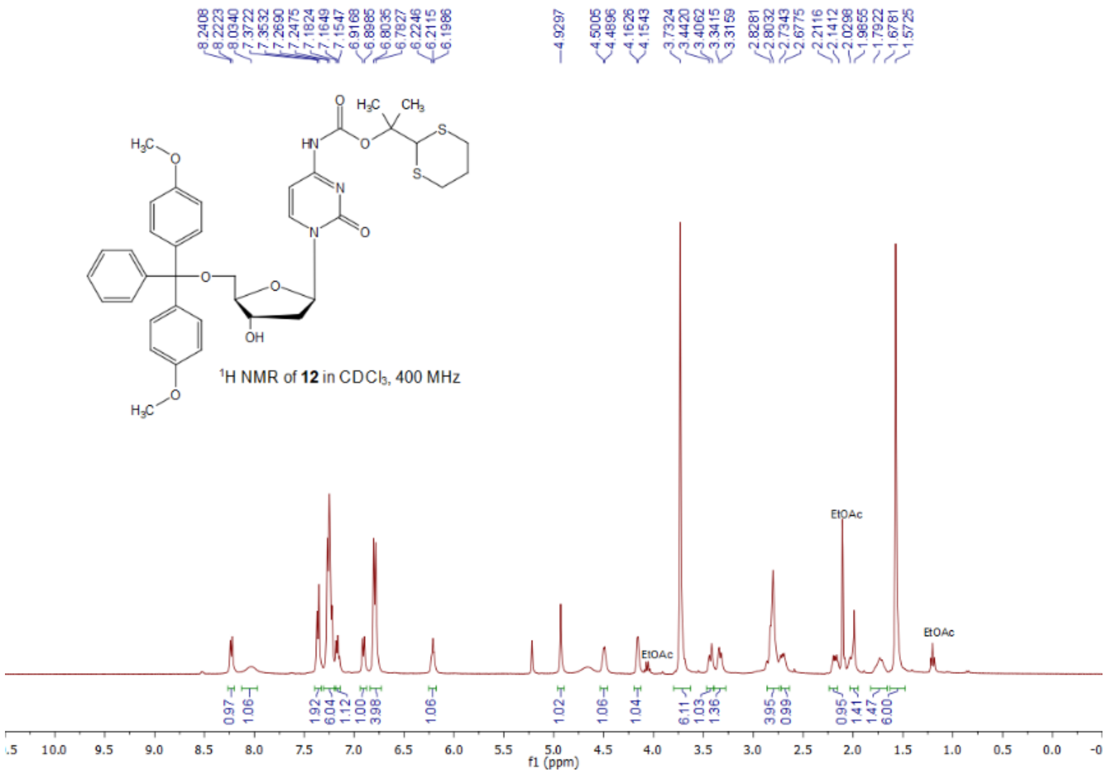

^{1}H NMR of **12** in CDCl3, 400 MHz
EtOAc
f1 (ppm)

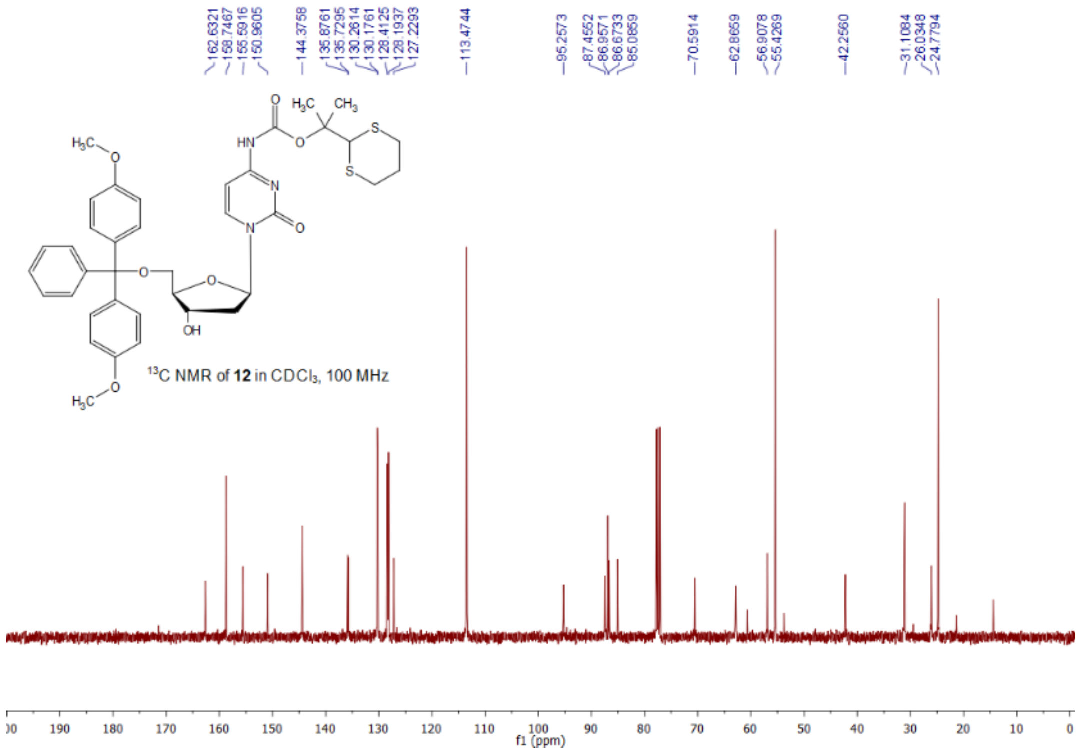

^{13}C NMR of **12** in CDCl3, 100 MHz
f1 (ppm)

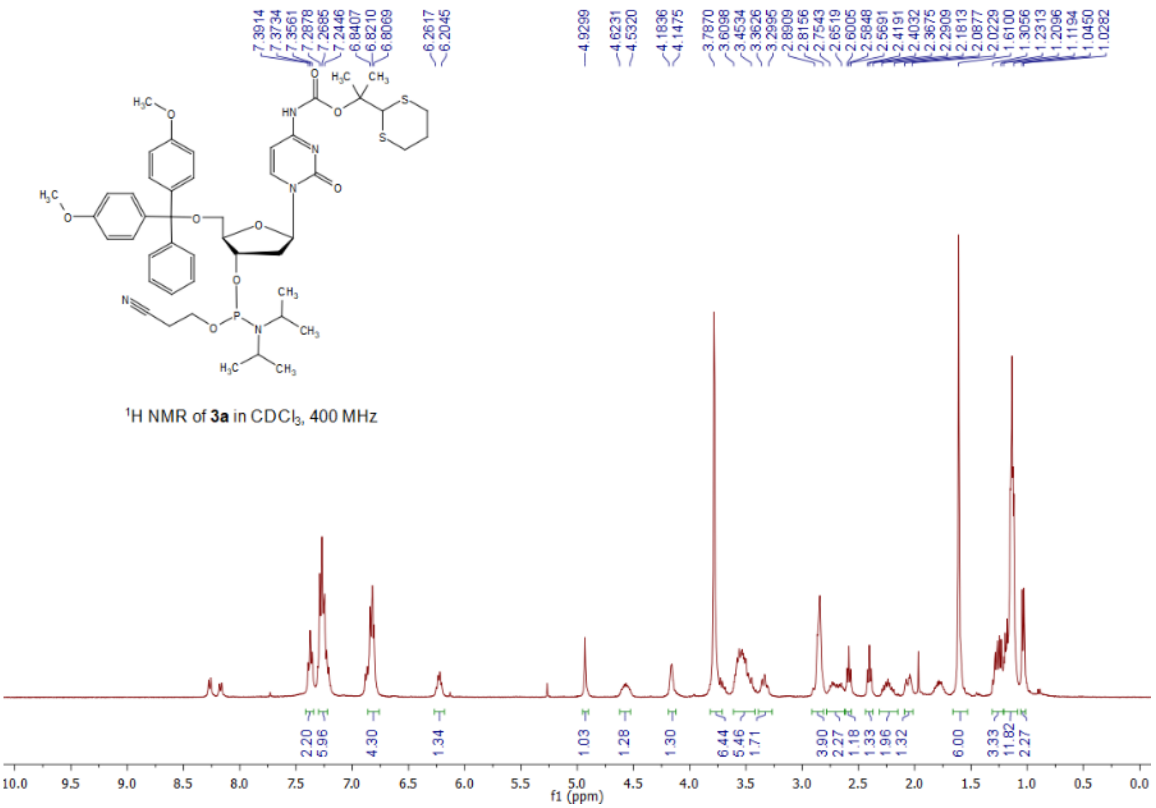

^{1}H NMR of **3a** in $CDCl_3$, 400 MHz

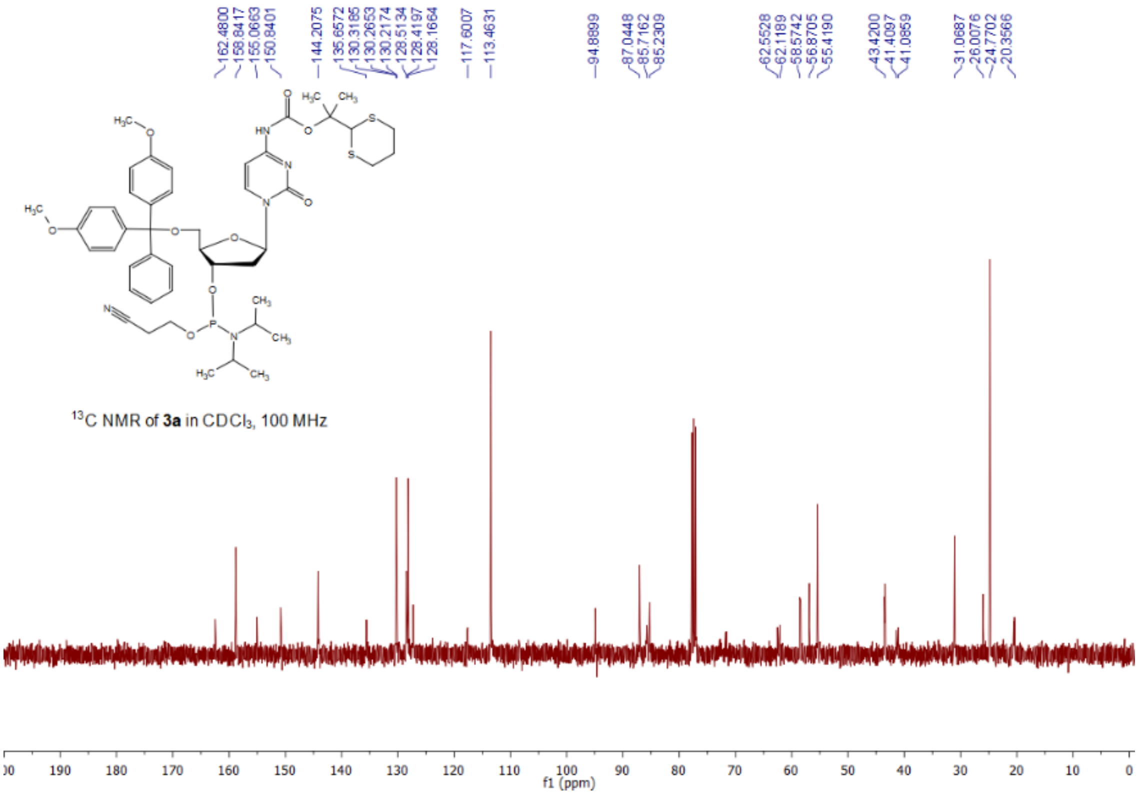

^{13}C NMR of **3a** in $CDCl_3$, 100 MHz

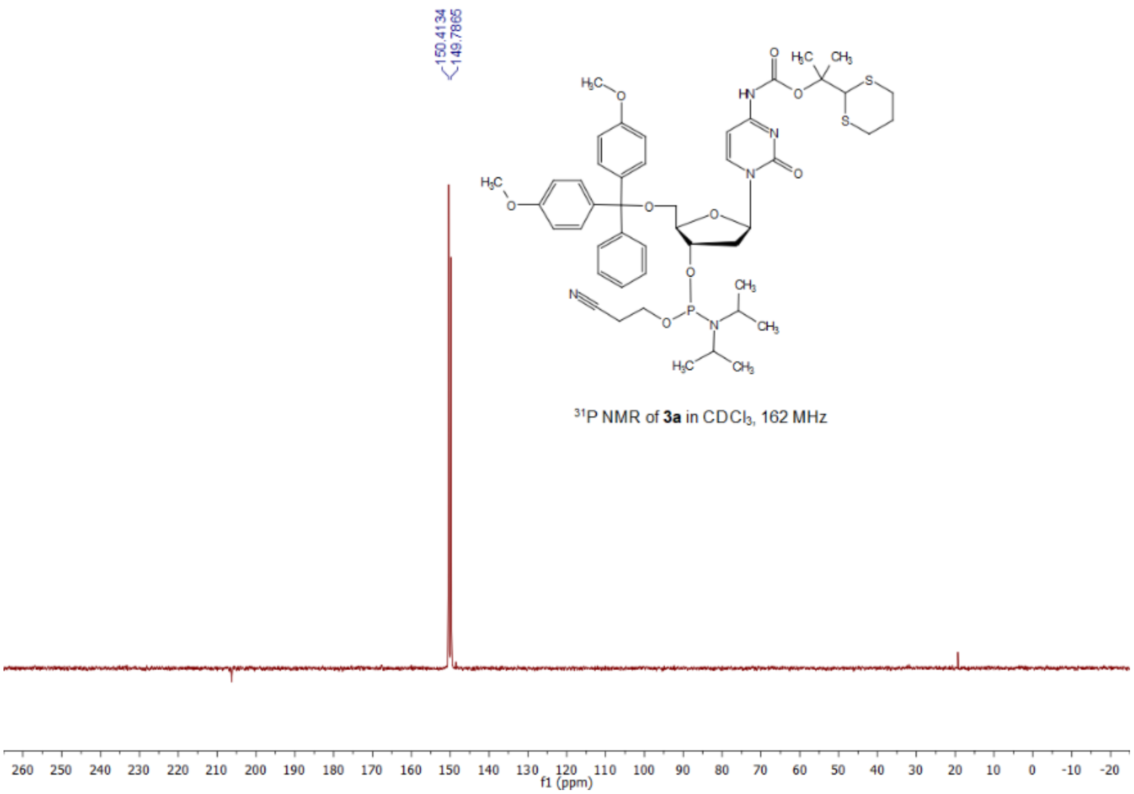

^{31}P NMR of **3a** in $CDCl_3$, 162 MHz

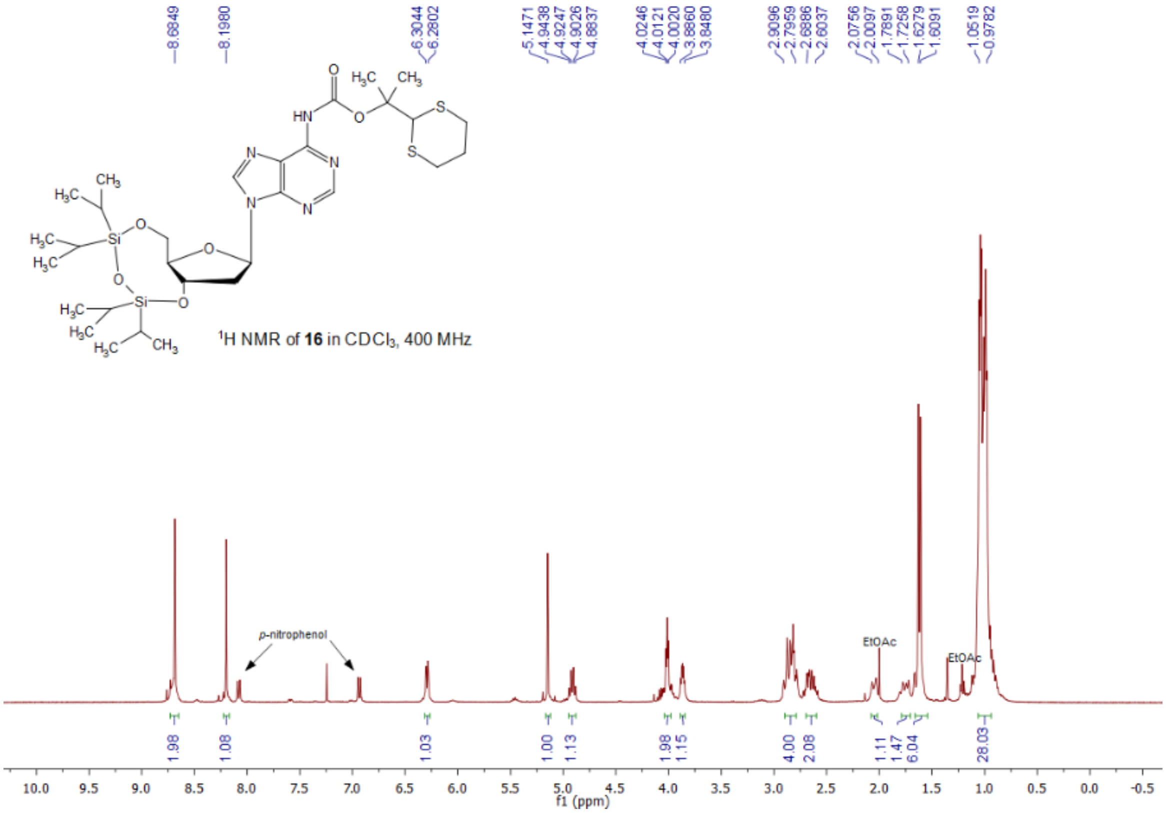

^{1}H NMR of **16** in $CDCl_3$, 400 MHz

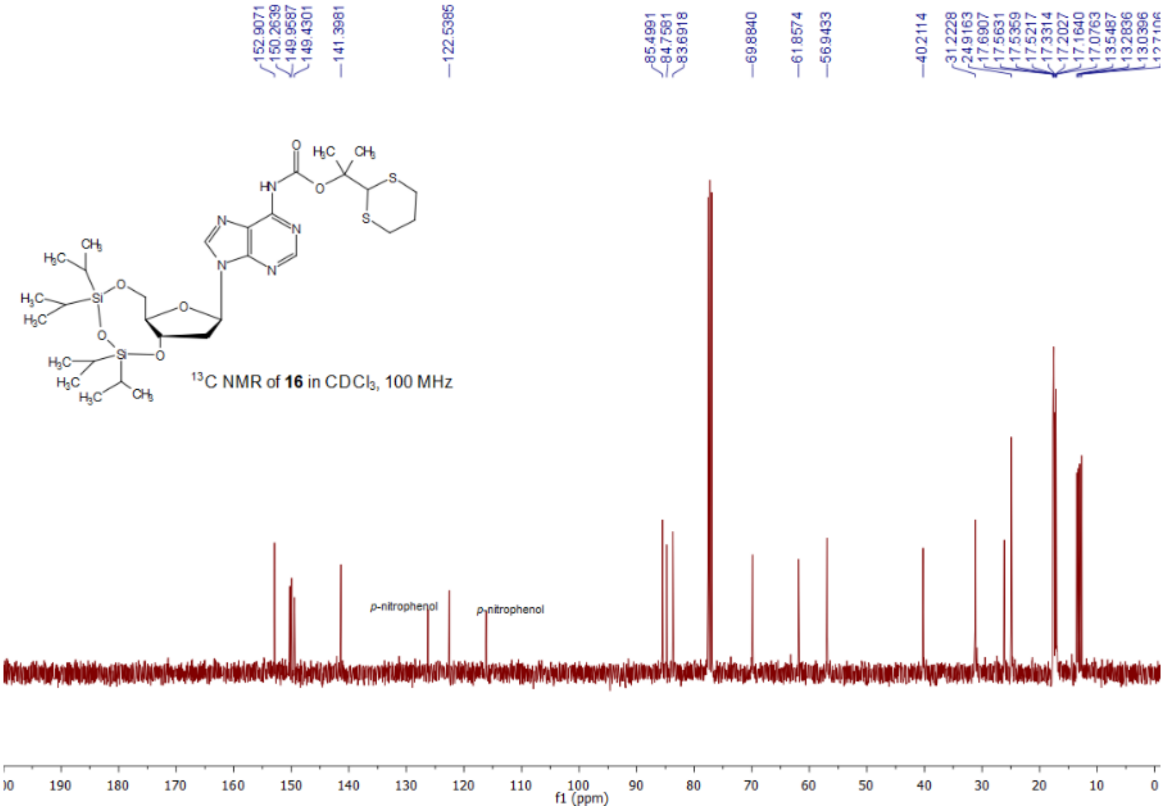

^{13}C NMR of **16** in $CDCl_3$, 100 MHz

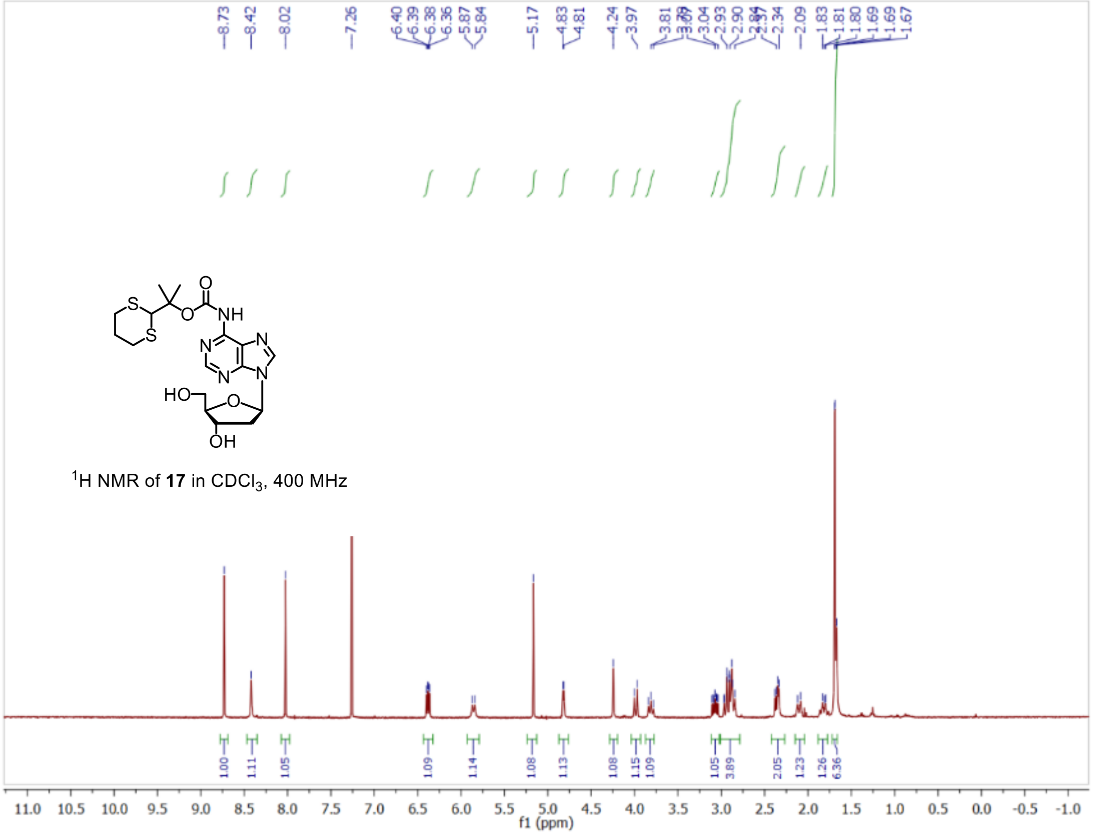

^{1}H NMR of **17** in $CDCl_3$, 400 MHz

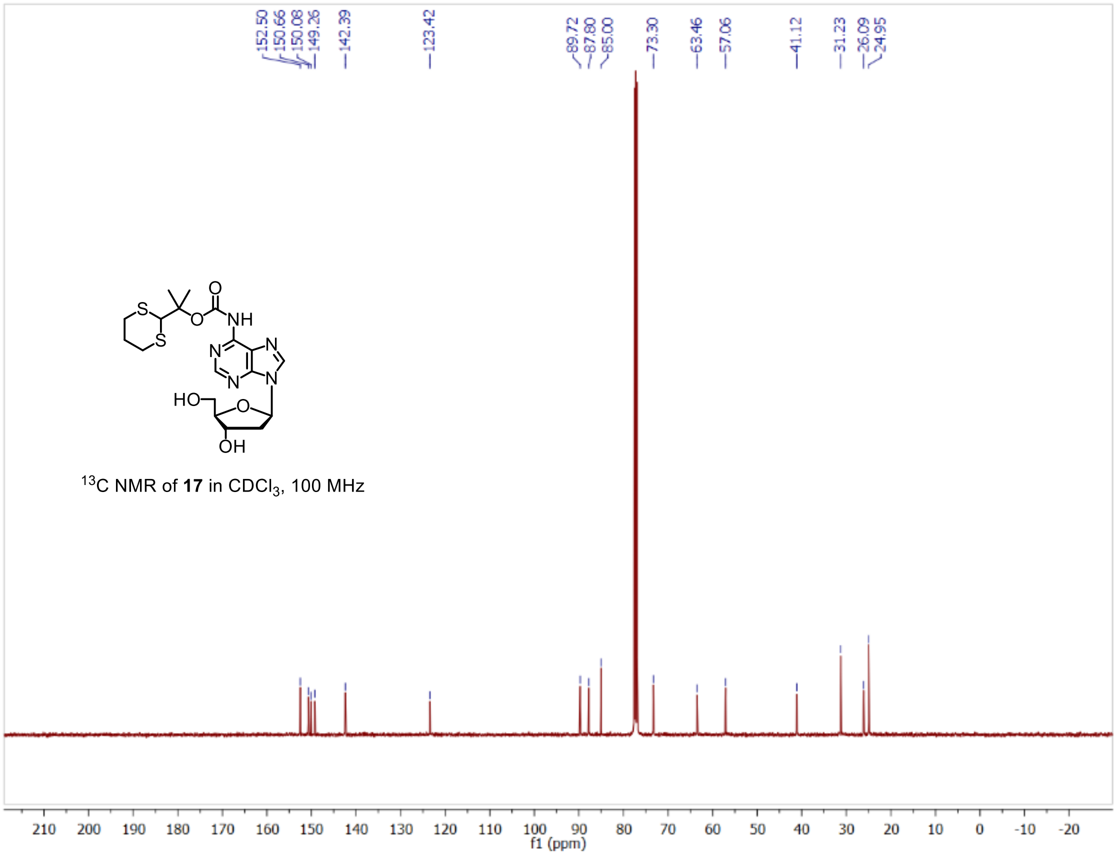

^{13}C NMR of **17** in $CDCl_3$, 100 MHz

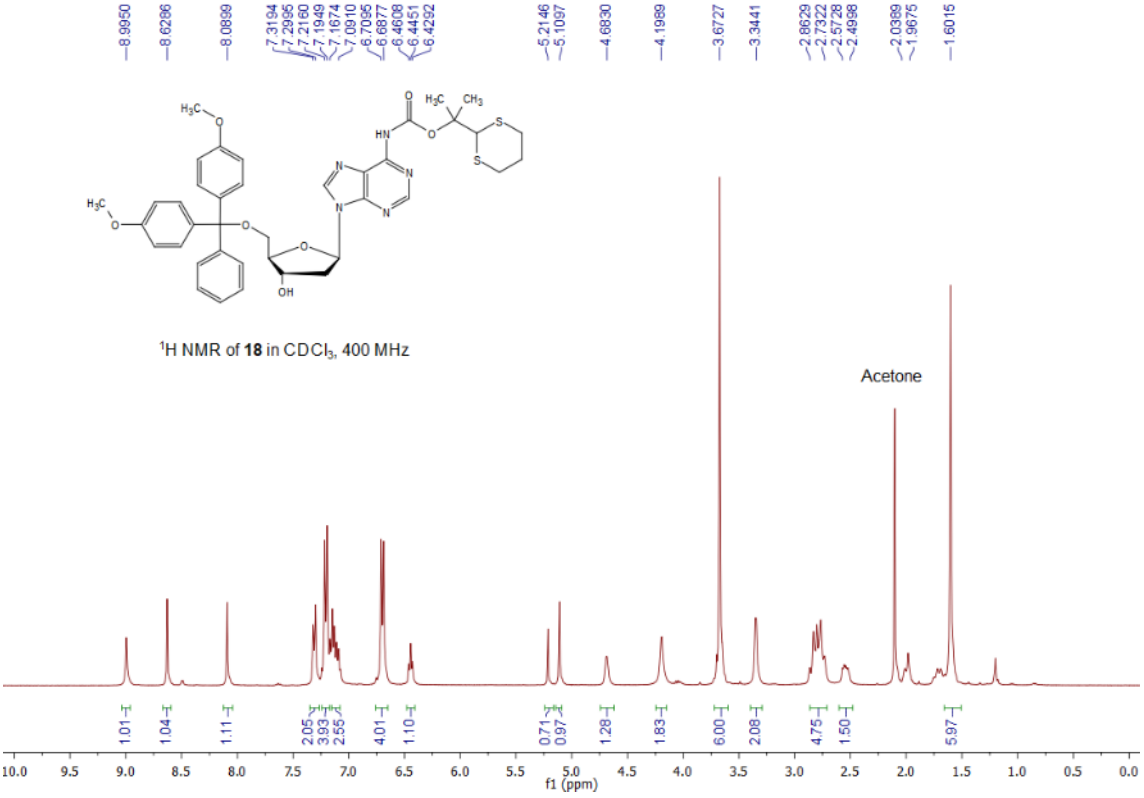

^{1}H NMR of **18** in $CDCl_3$, 400 MHz

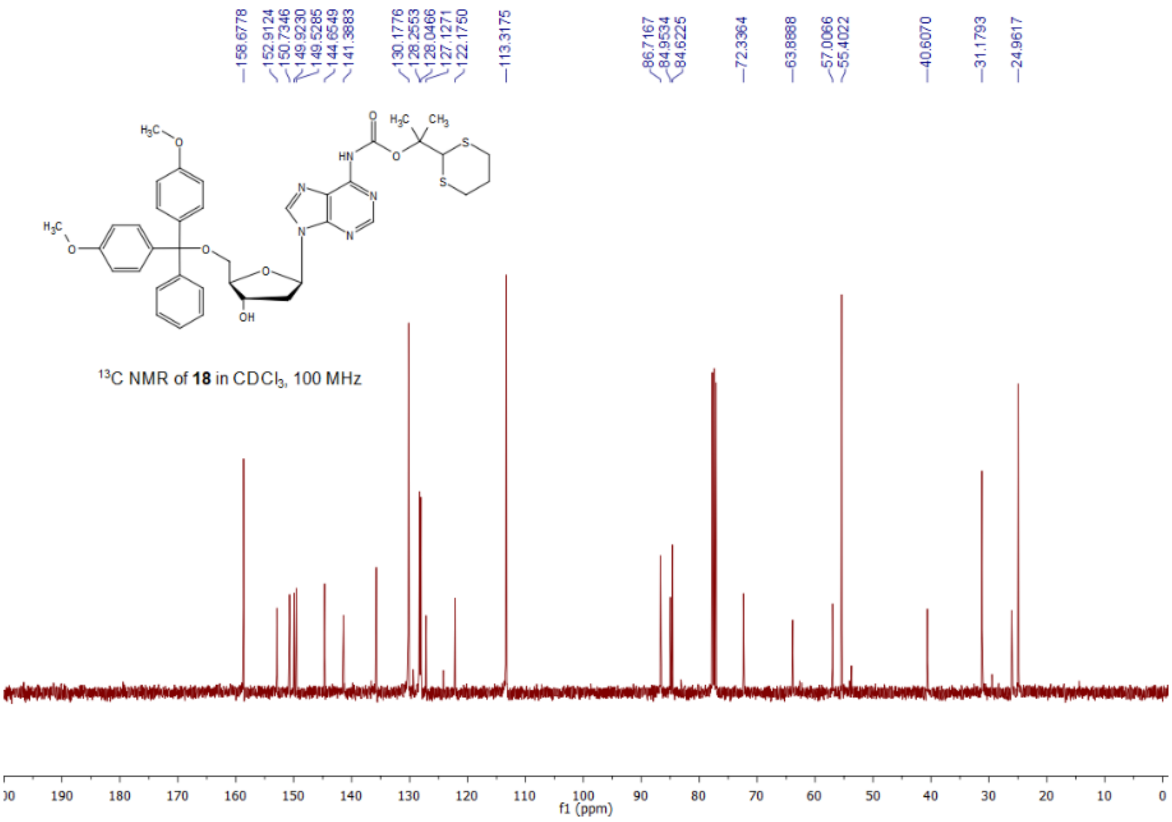

^{13}C NMR of **18** in $CDCl_3$, 100 MHz

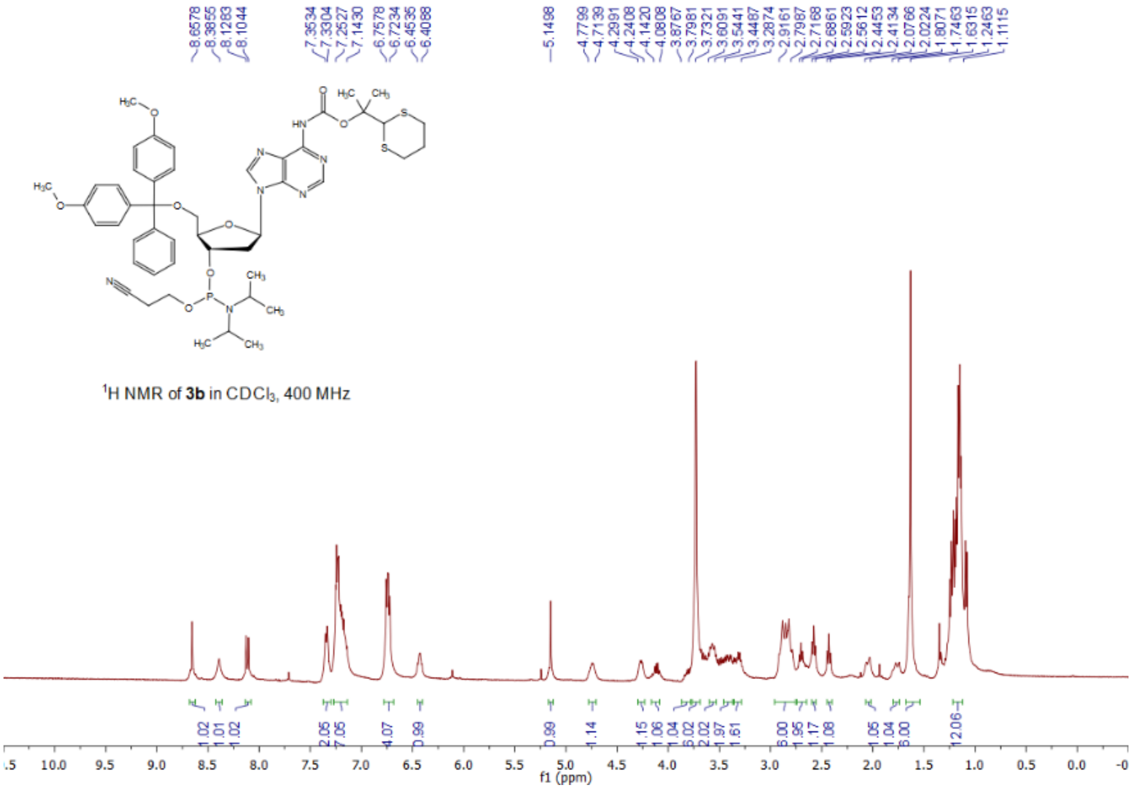

^{1}H NMR of **3b** in $CDCl_3$, 400 MHz

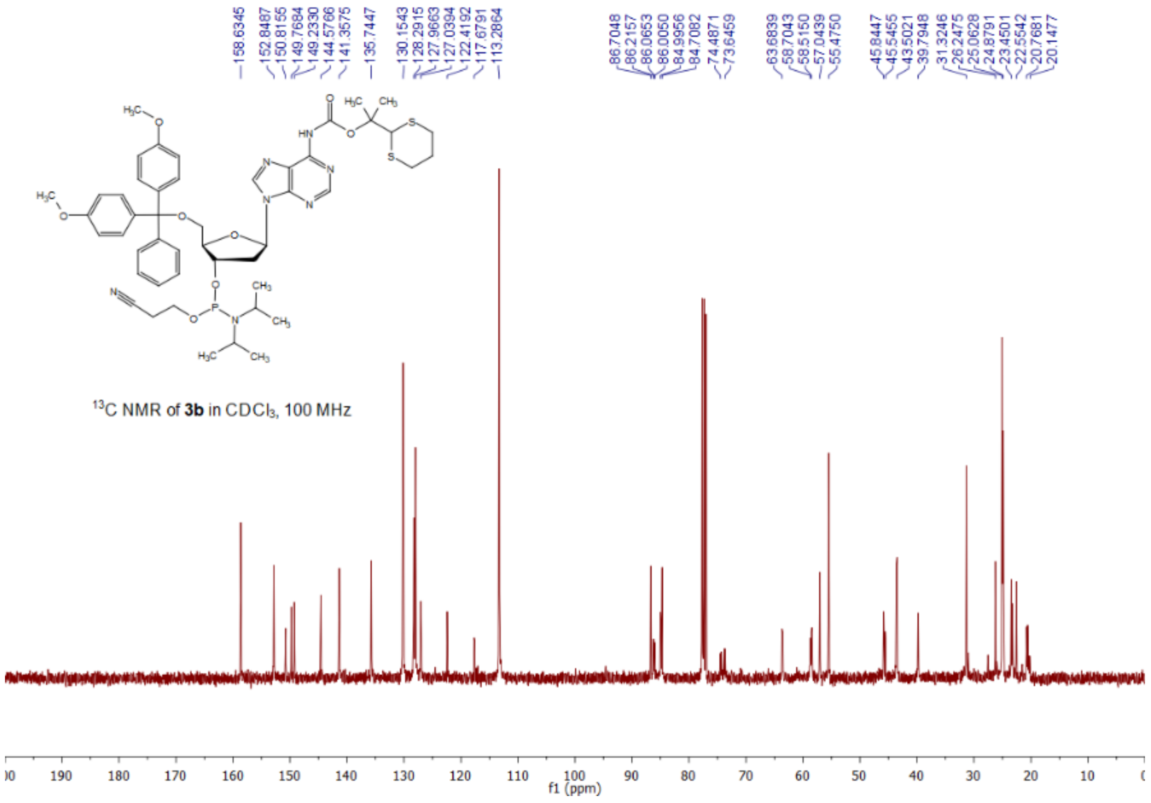

^{13}C NMR of **3b** in $CDCl_3$, 100 MHz

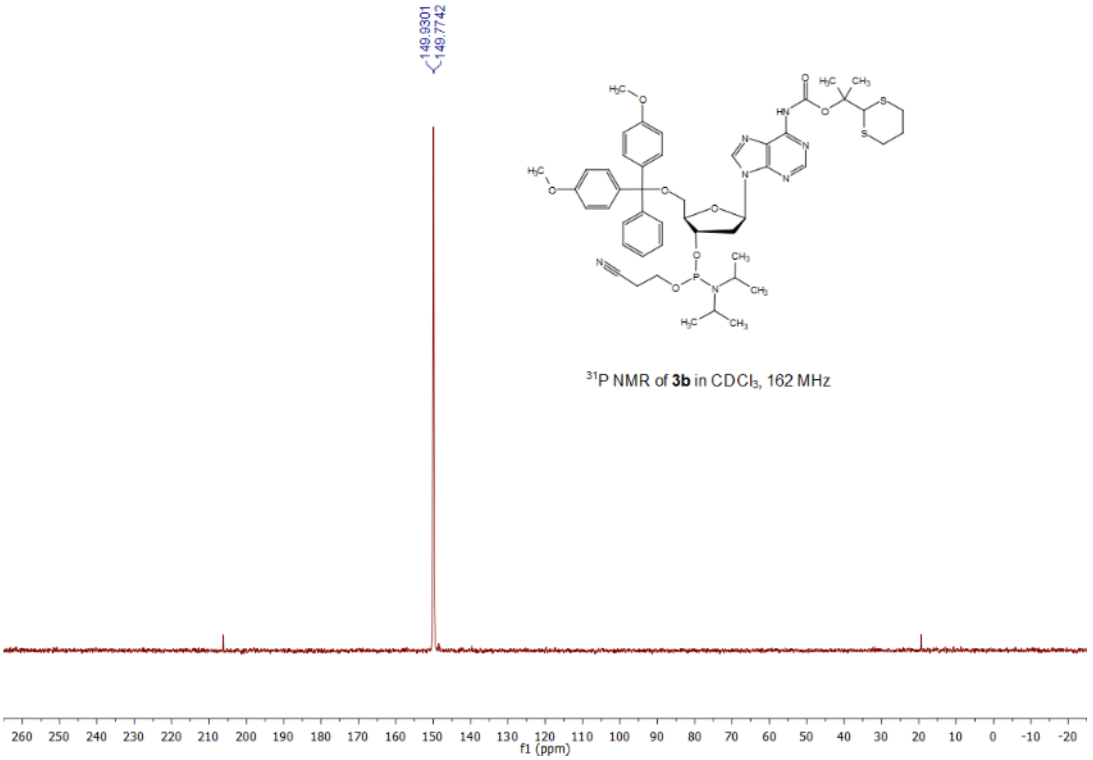

^{31}P NMR of **3b** in $CDCl_3$, 162 MHz

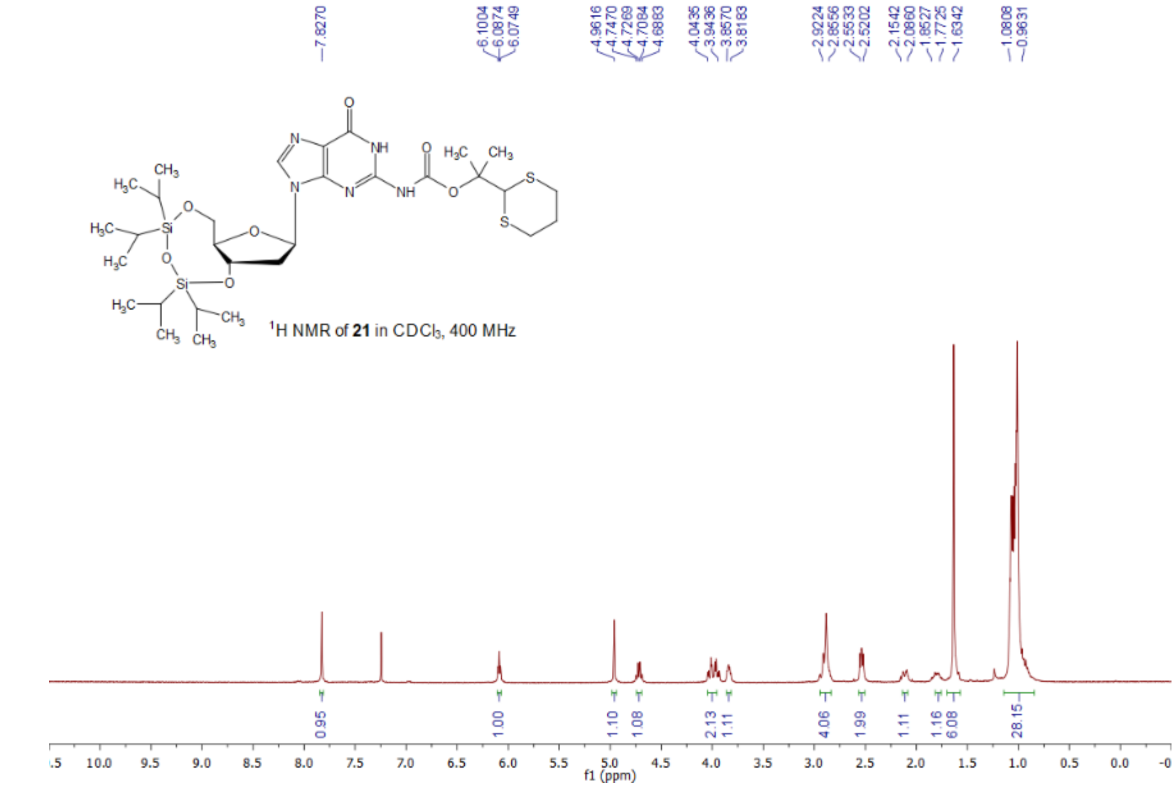
7.8270
6.1004
6.0874
6.0749
4.9616
4.7470
4.7269
4.7084
4.6883
4.0435
3.9436
3.8570
3.8183
2.9224
2.8556
2.5533
2.5202
2.1542
2.0860
1.8527
1.7725
1.6342
1.0808
0.9631
0.95
1.00
1.10
1.08
2.13
1.11
4.06
1.99
1.11
1.16
6.08
28.15
f1 (ppm)
^{1}H NMR of 21 in CDCl3, 400 MHz

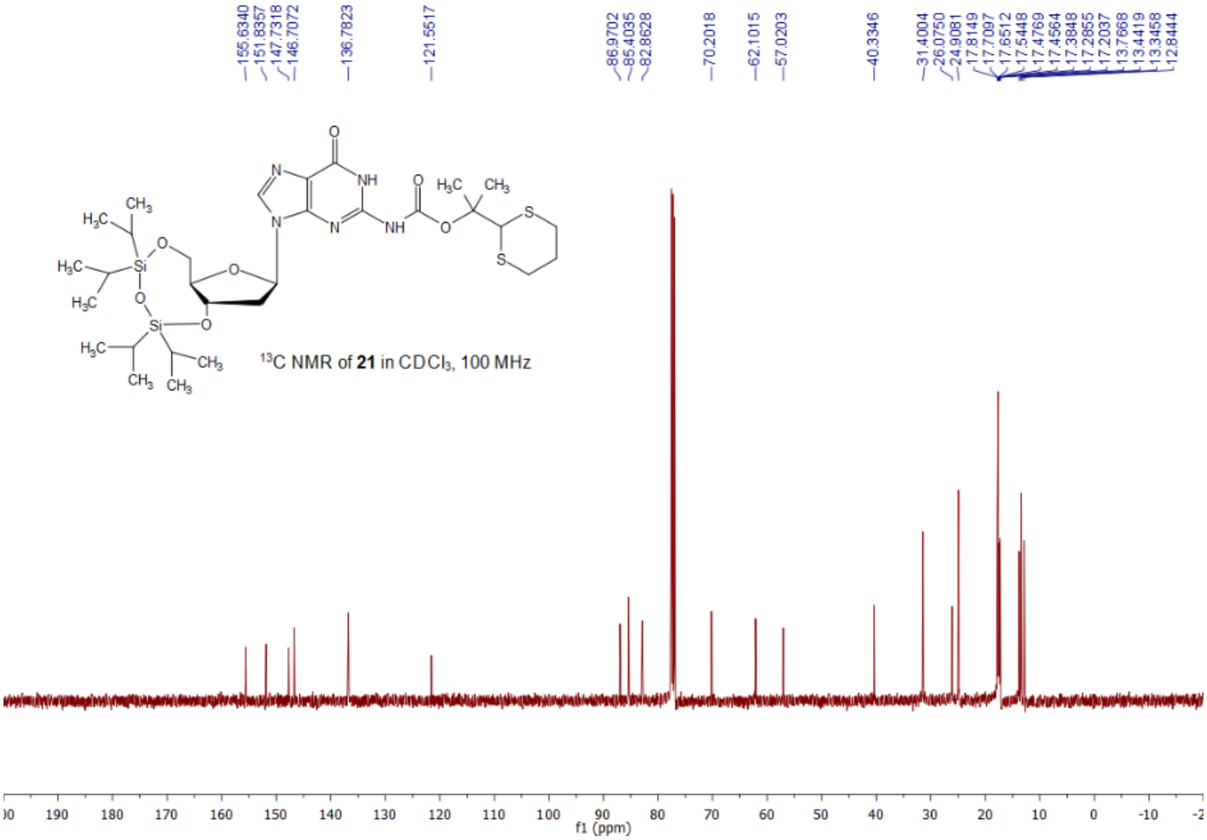
155.6340
151.8357
147.7318
146.7072
136.7823
121.5517
86.9702
85.4035
82.8628
70.2018
62.1015
57.0203
40.3346
31.4004
26.0750
24.9081
17.8149
17.7097
17.6512
17.5448
17.4769
17.4564
17.3848
17.2855
17.2037
13.7668
13.4419
13.3458
12.8444
f1 (ppm)
^{13}C NMR of 21 in CDCl3, 100 MHz

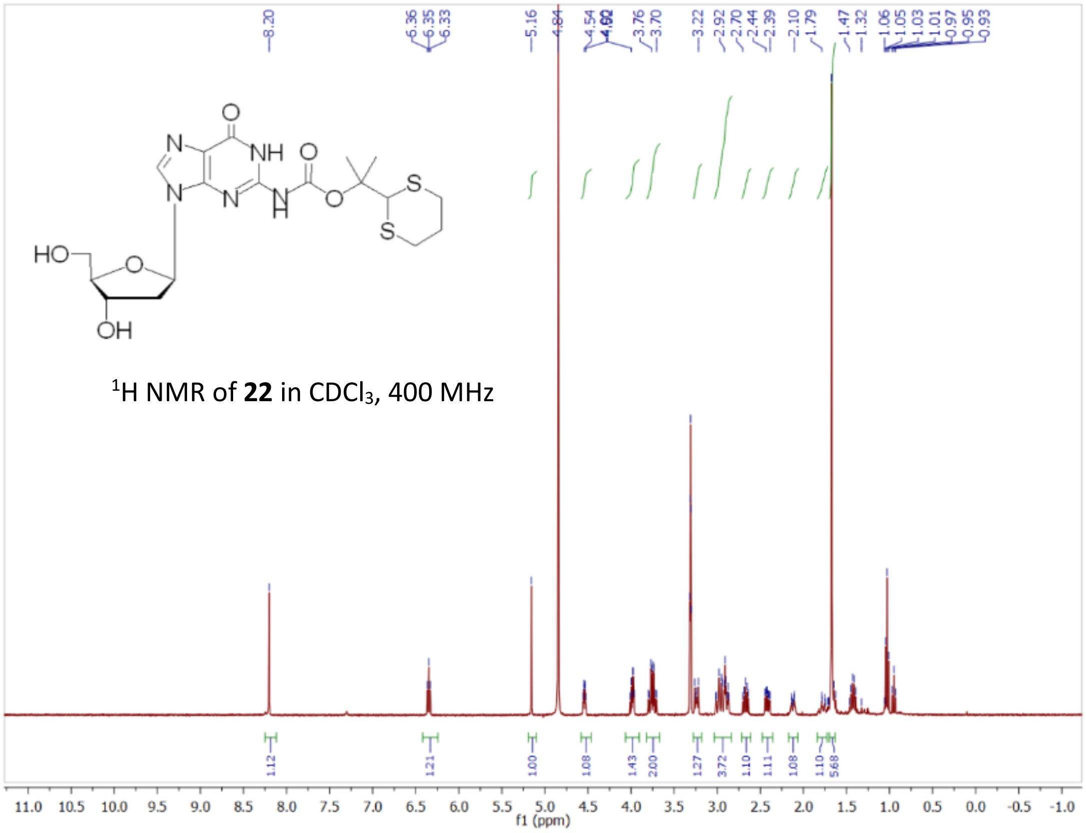

^{1}H NMR of **22** in $CDCl_3$, 400 MHz

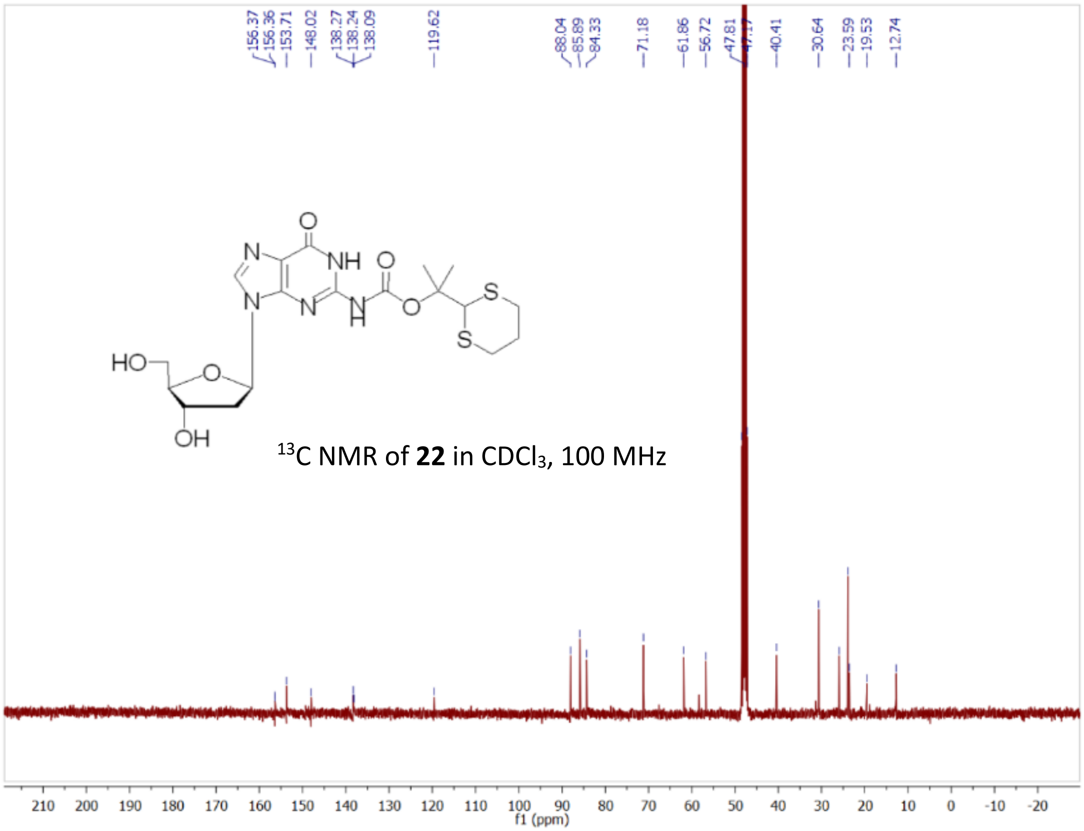

^{13}C NMR of **22** in $CDCl_3$, 100 MHz

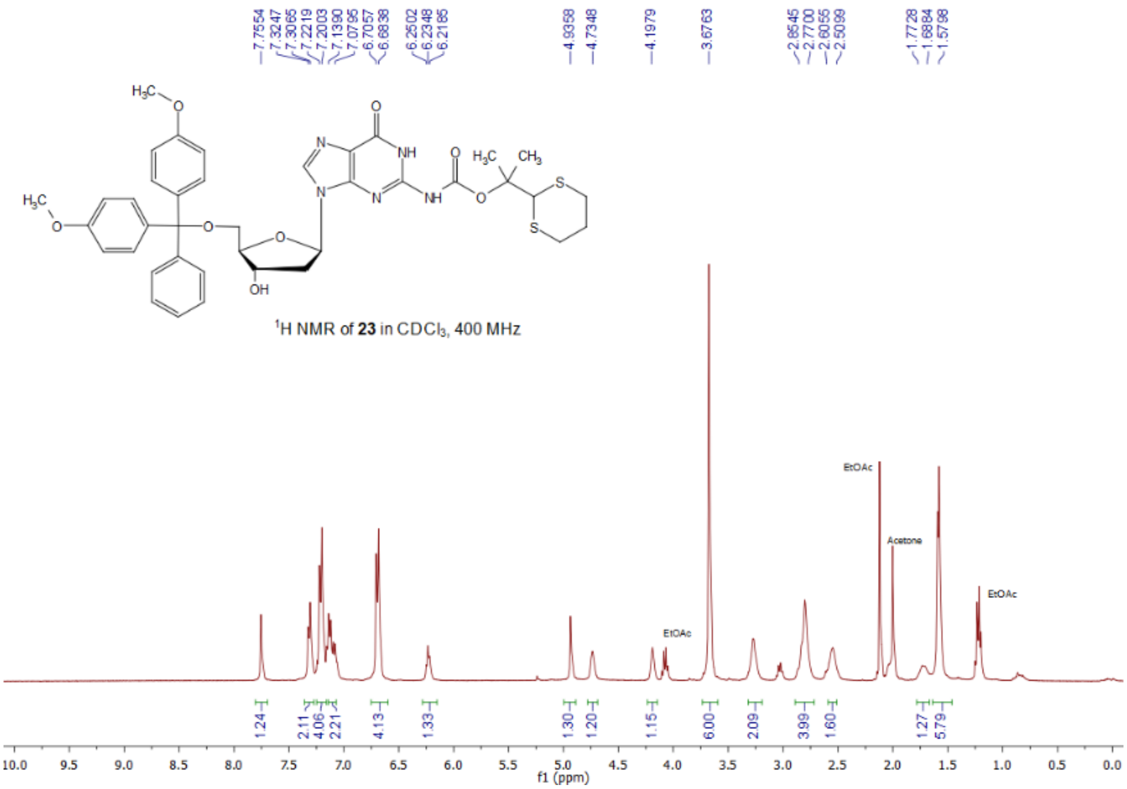

^{1}H NMR of **23** in $CDCl_3$, 400 MHz

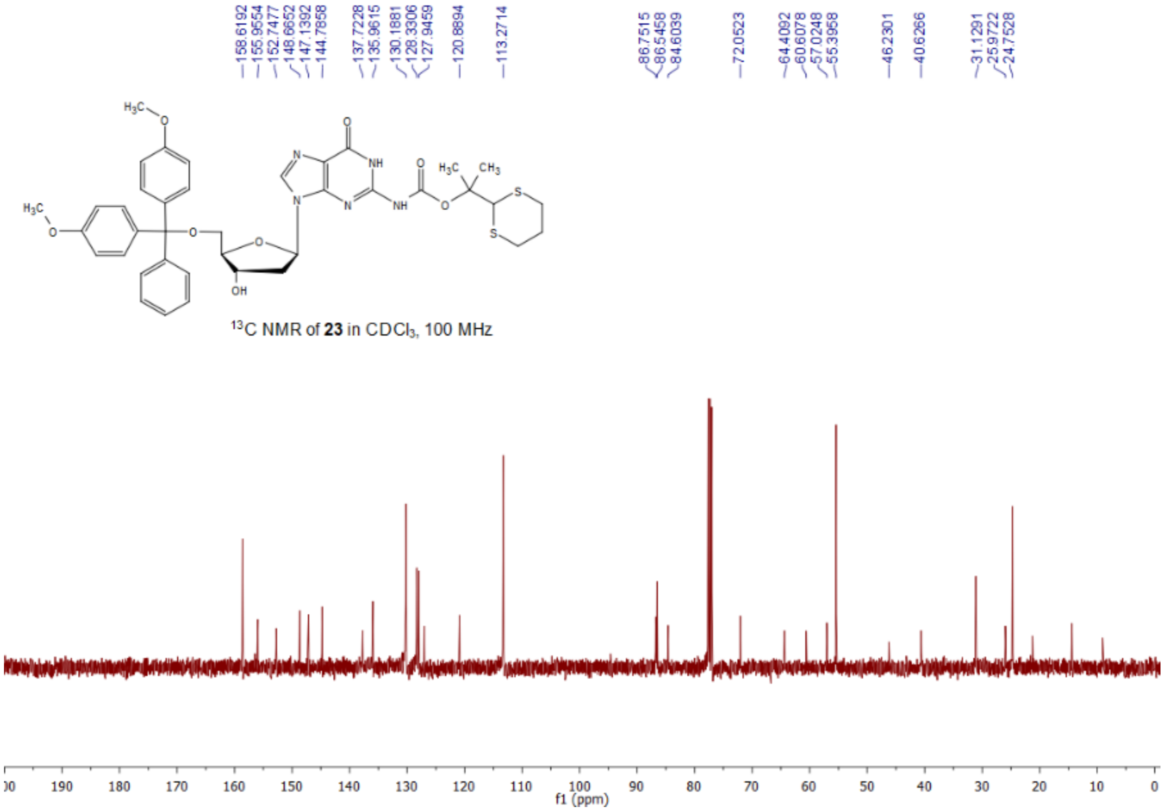

^{13}C NMR of **23** in $CDCl_3$, 100 MHz

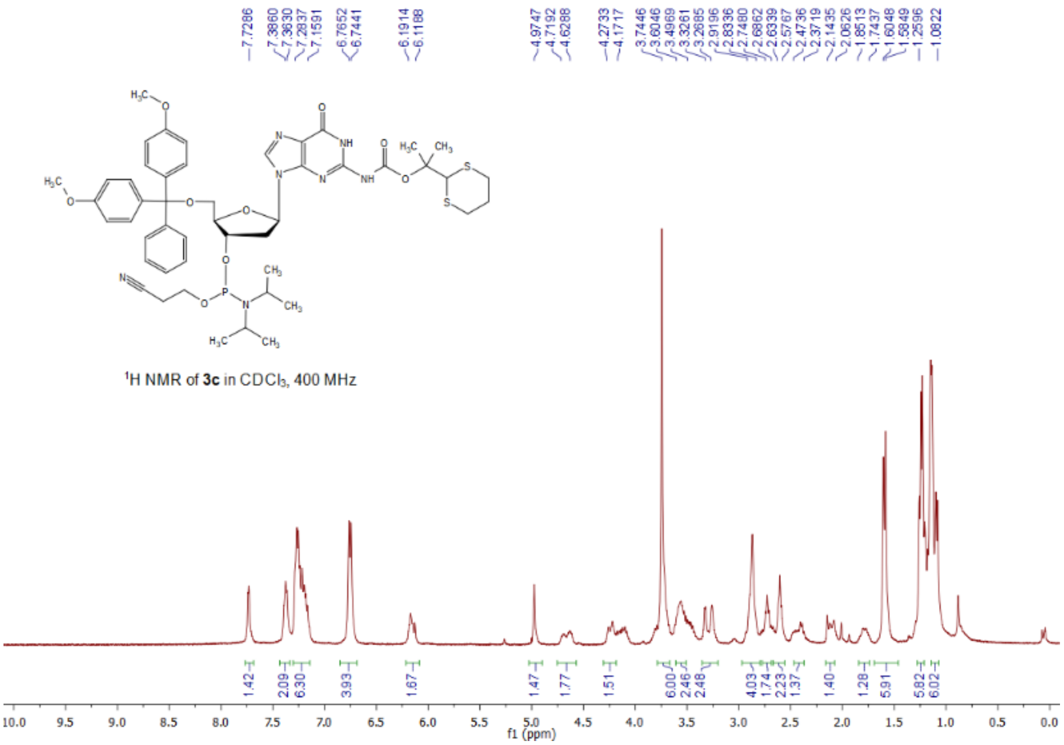

^{1}H NMR of **3c** in $CDCl_3$, 400 MHz

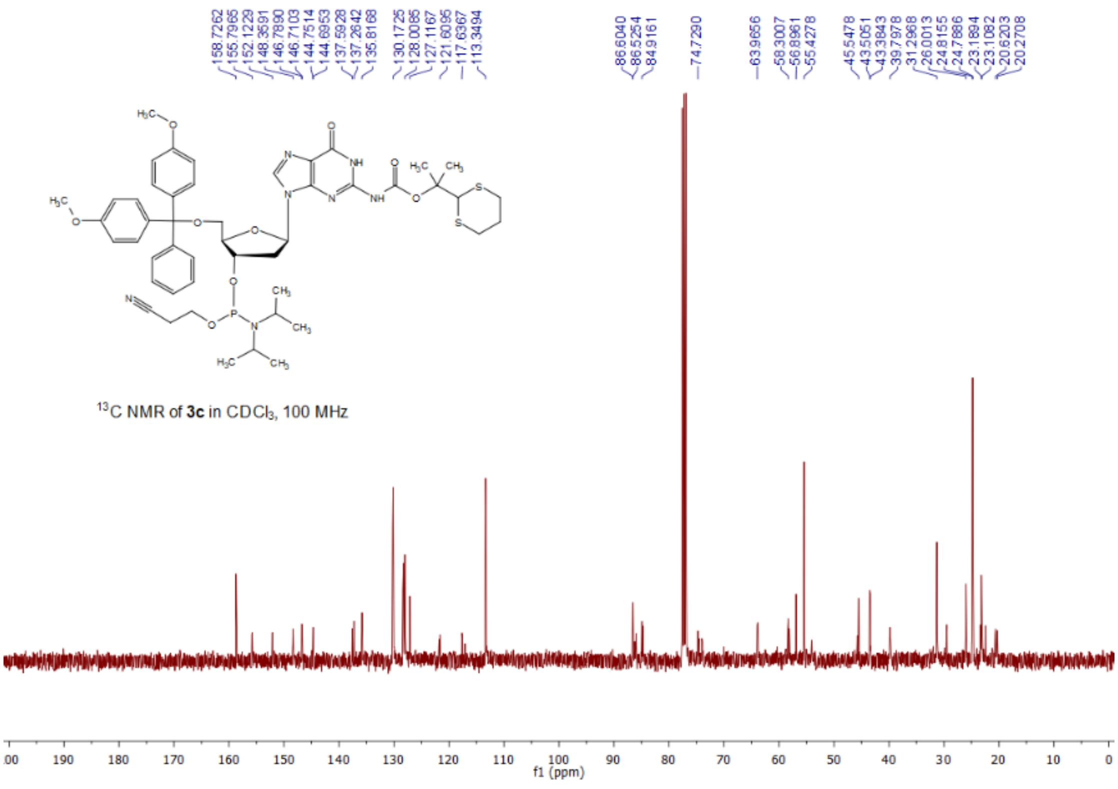

^{13}C NMR of **3c** in $CDCl_3$, 100 MHz

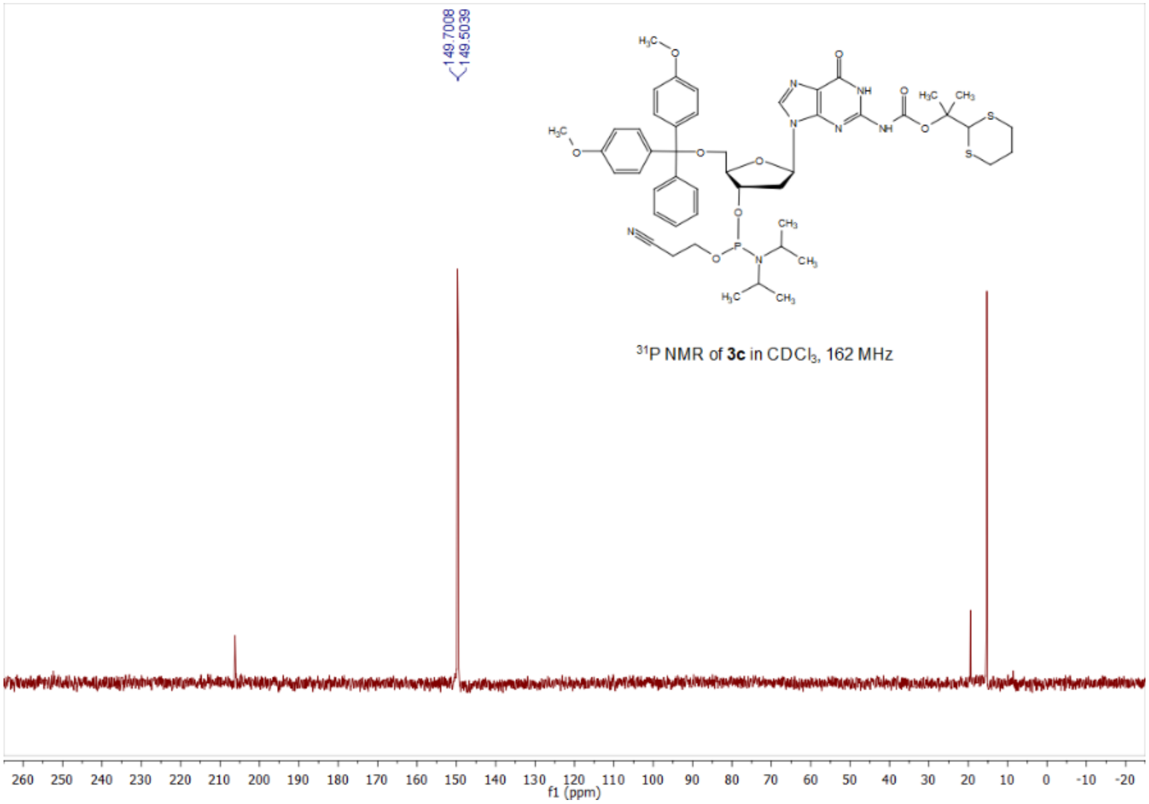
149.7008
149.5039
³¹P NMR of 3c in CDCl₃, 162 MHz
f1 (ppm)

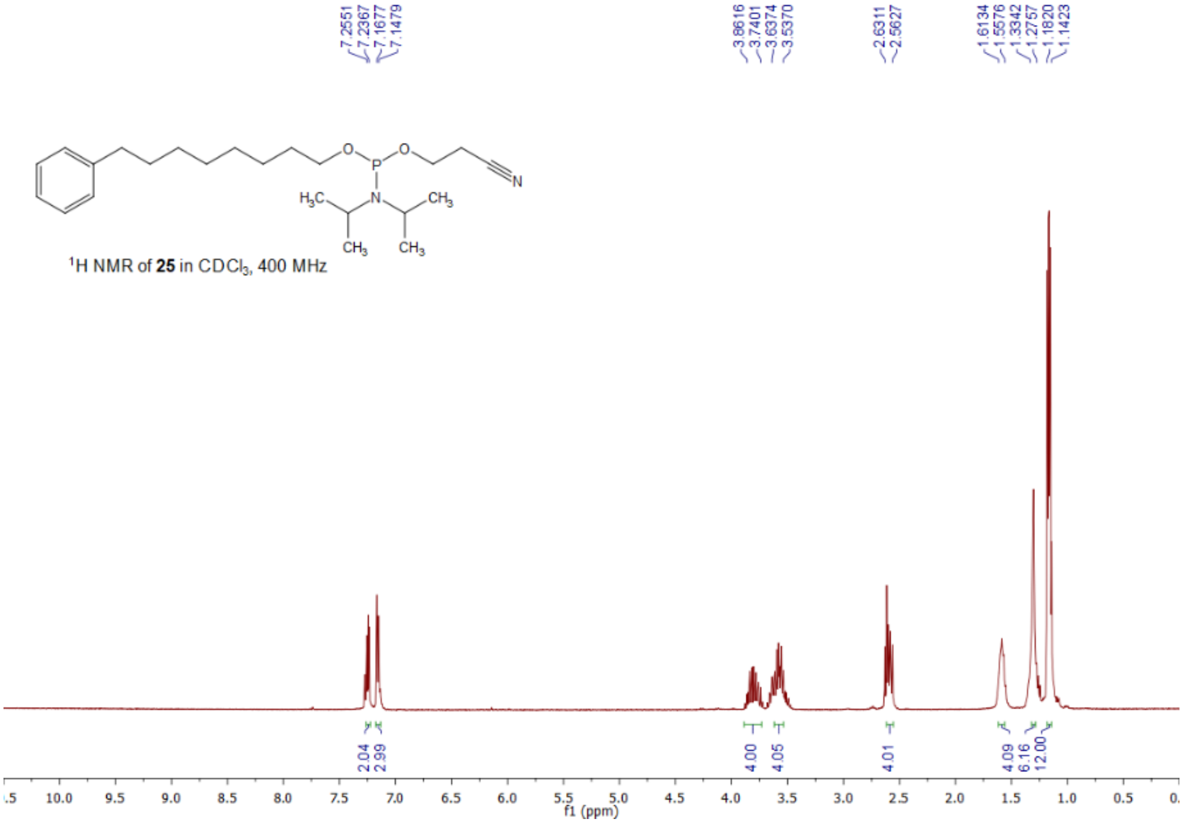
7.2551
7.2367
7.1677
7.1479
3.8616
3.7401
3.6374
3.5370
2.6311
2.5627
1.6134
1.5576
1.3342
1.2757
1.1820
1.1423
¹H NMR of 25 in CDCl₃, 400 MHz
2.04
2.99
4.00
4.05
4.01
4.09
6.16
12.00
f1 (ppm)

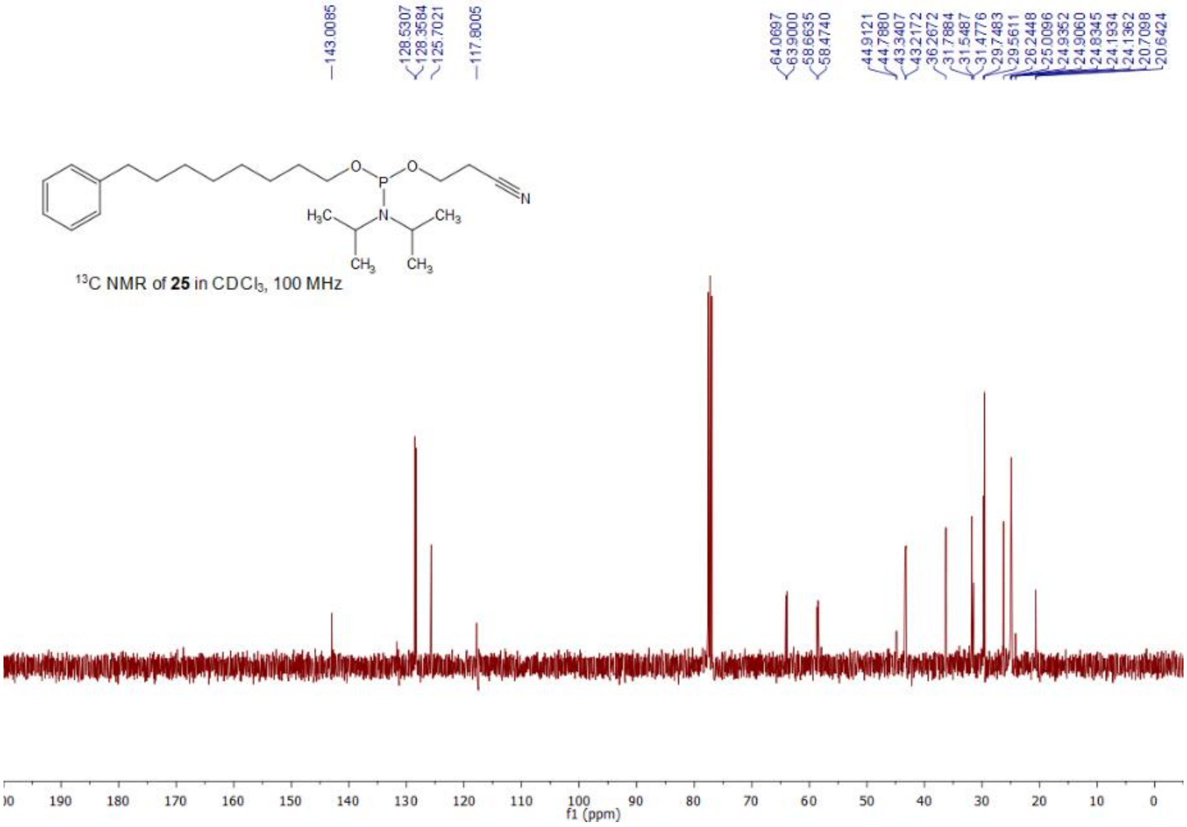
143.0085
128.5307
128.3584
125.7021
117.8005
64.0697
63.9000
58.6635
58.4740
44.9121
44.7880
43.3407
43.2172
36.2672
31.7884
31.5487
31.4776
29.7483
29.5611
26.2448
25.0096
24.9352
24.9060
24.8345
24.1934
24.1362
20.7098
20.6424
¹³C NMR of 25 in CDCl3, 100 MHz
f1 (ppm)

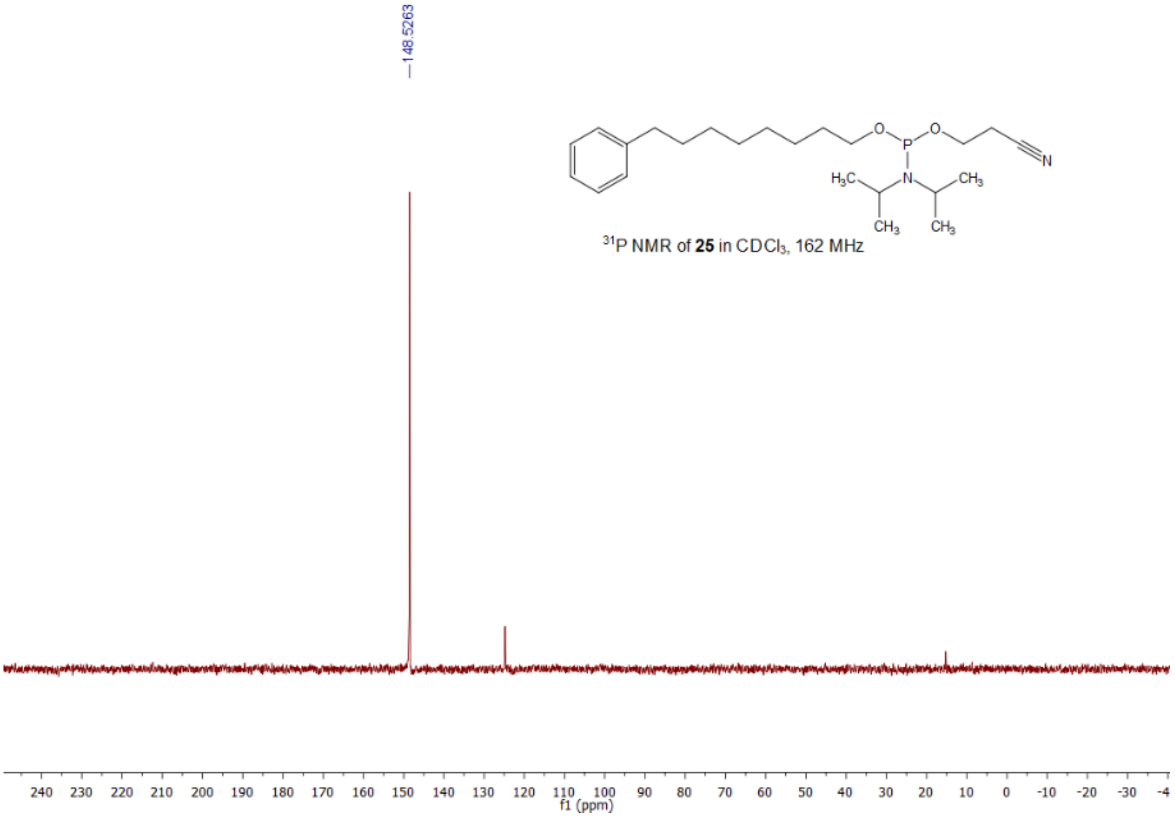
148.5263
³¹P NMR of 25 in CDCl3, 162 MHz
f1 (ppm)

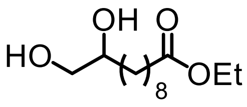

^{1}H NMR of **28** in CD_3OD, 400 MHz

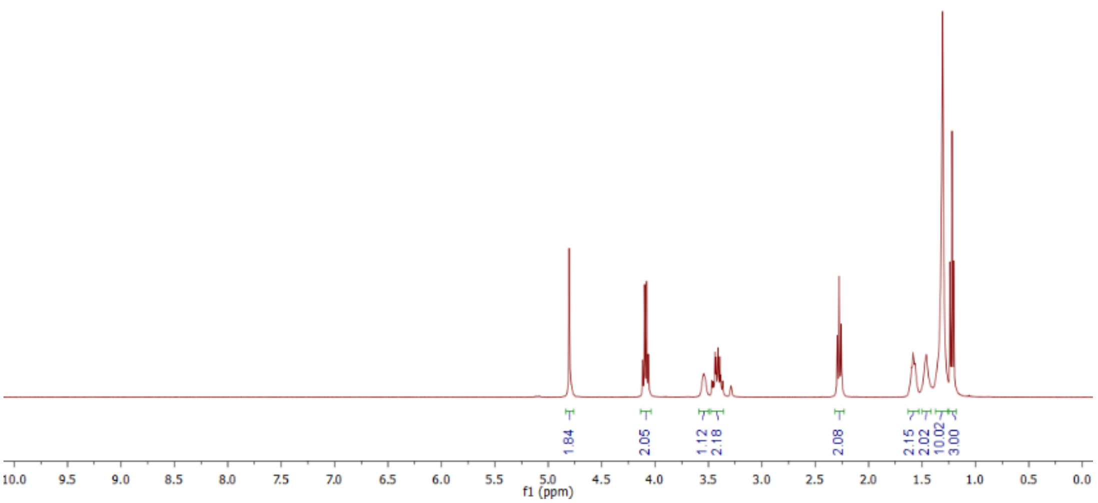

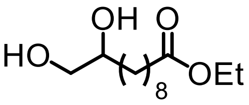

^{13}C NMR of **28** in CD_3OD, 100 MHz

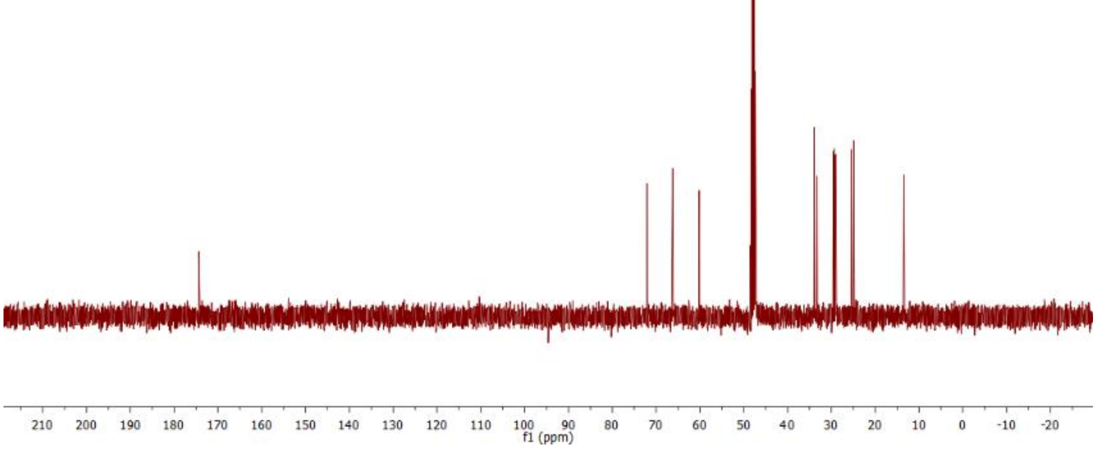

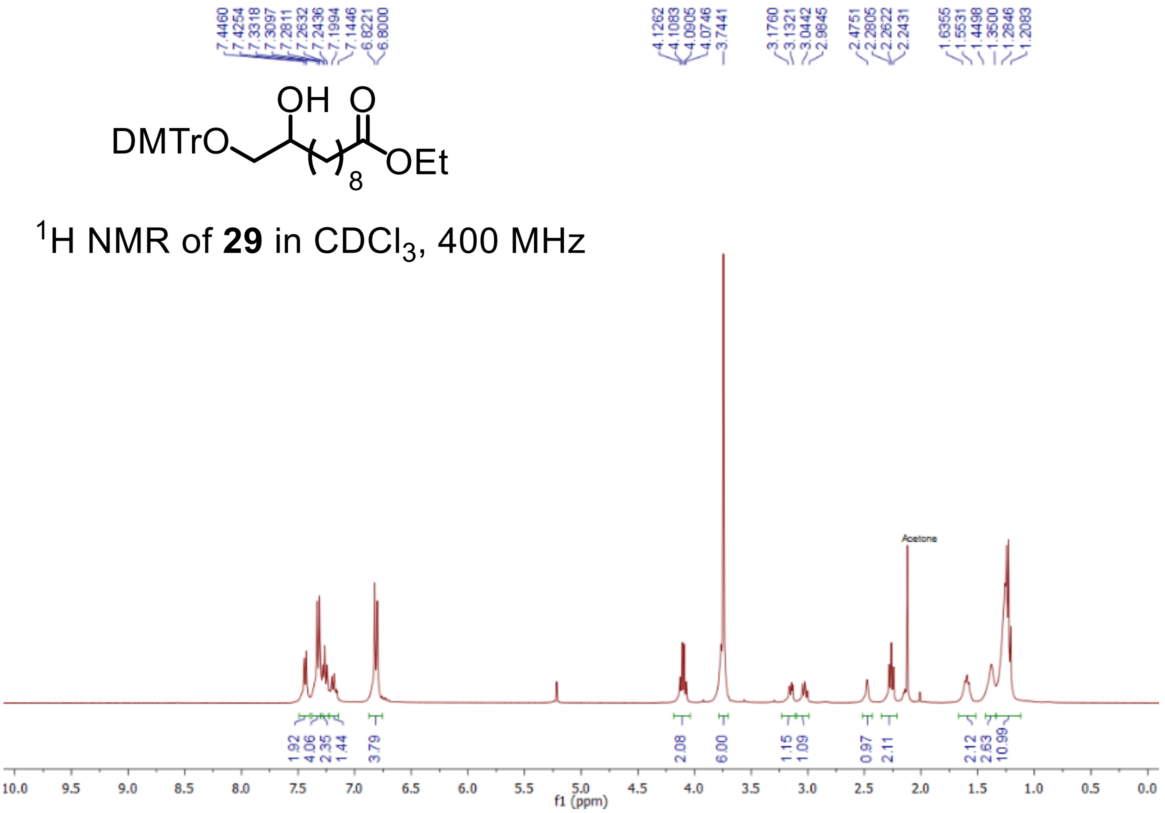

^{1}H NMR of **29** in $CDCl_3$, 400 MHz

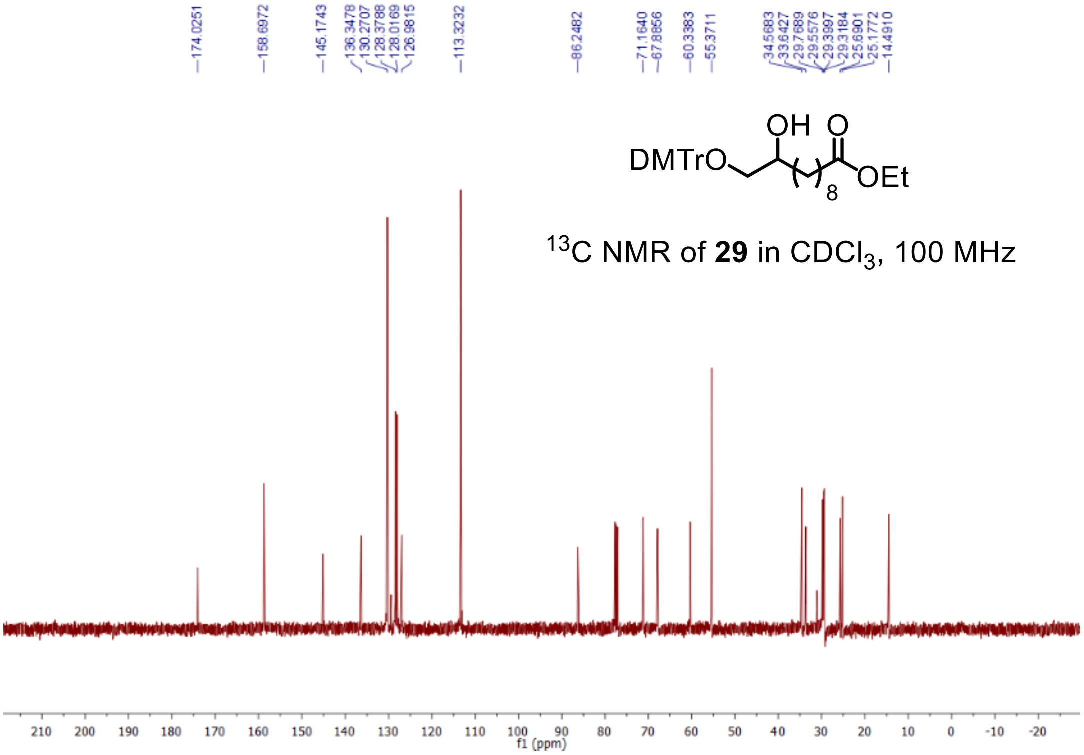

^{13}C NMR of **29** in $CDCl_3$, 100 MHz

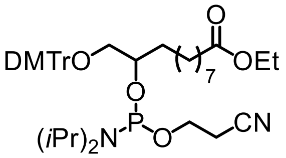

^{1}H NMR of **26a** in $CDCl_3$, 400 MHz

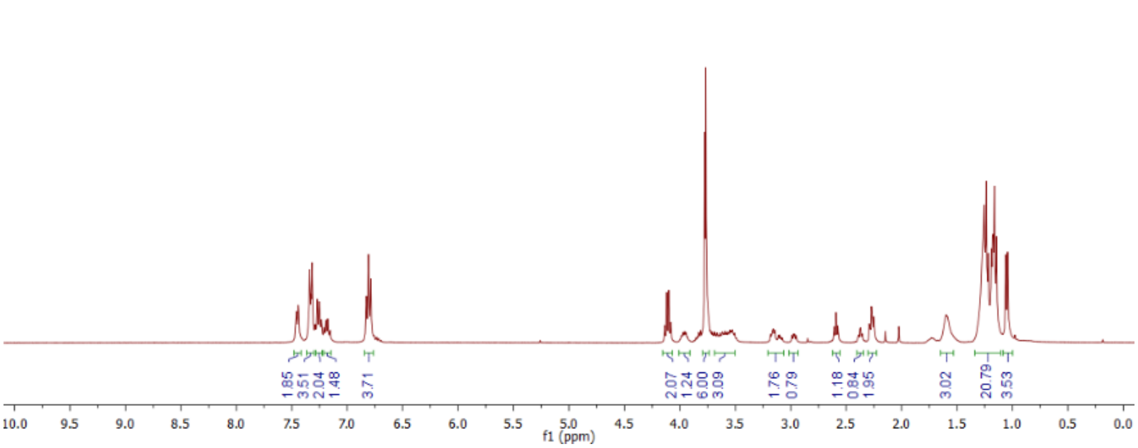

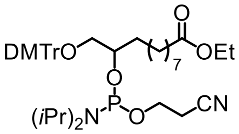

^{13}C NMR of **26a** in $CDCl_3$, 100 MHz

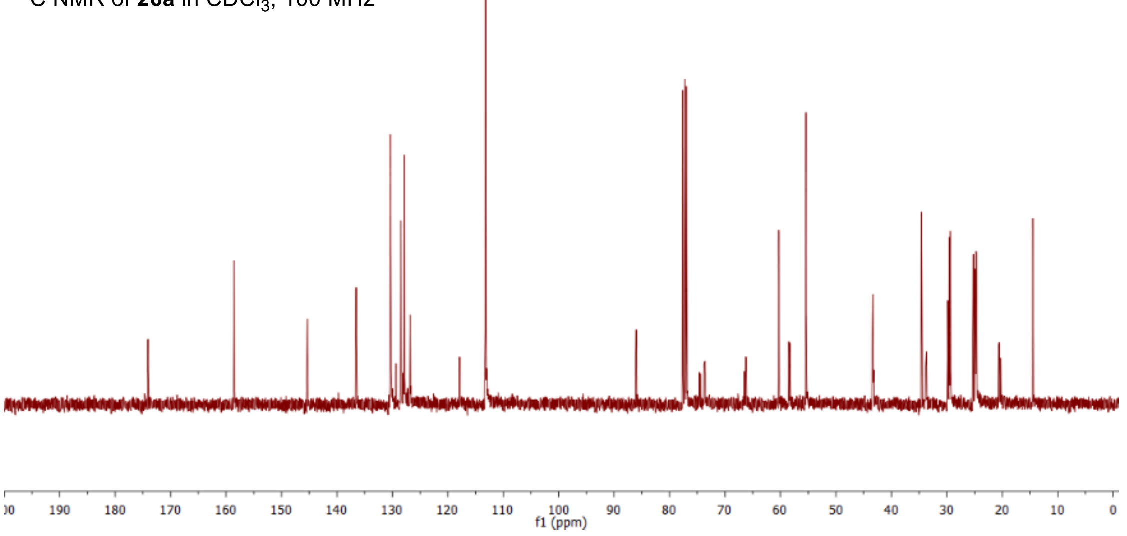

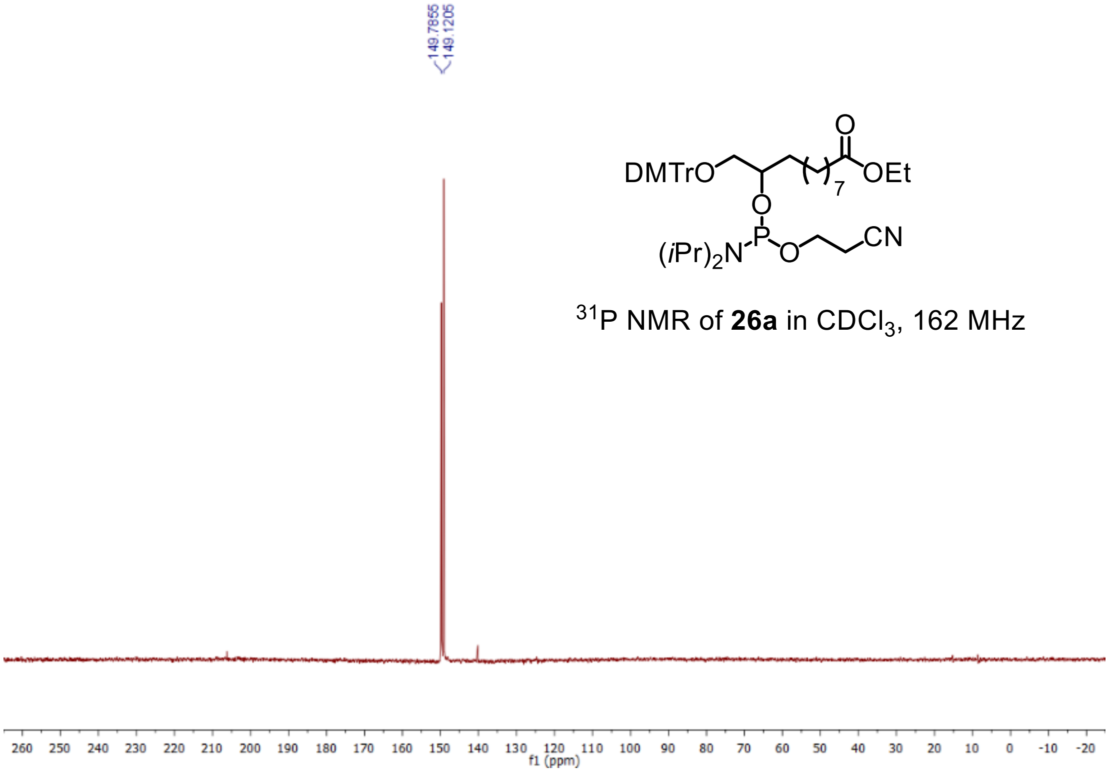

^{31}P NMR of **26a** in $CDCl_3$, 162 MHz
